# Supplementary material for: A Humanized Yeast Phenomic Model of Deoxycytidine Kinase to Predict Genetic Buffering of Nucleoside Analog Cytotoxicity
Source: Genes (Basel). 2019 Sep 30;10(10):770. doi: 10.3390/genes10100770 (PMC6826991; doi:10.3390/genes10100770)
Supplement: Supplementary file 1 [file genes-10-00770-s001.zip › Additional_Files/Additional_File7_GOTermHeatmaps.zip!/Additional_File7_GOTermHeatmaps/regulation_of_transcription_from_RNA_polymerase_II_promoter.pdf]

regulation of transcription from RNA polymerase II promoter

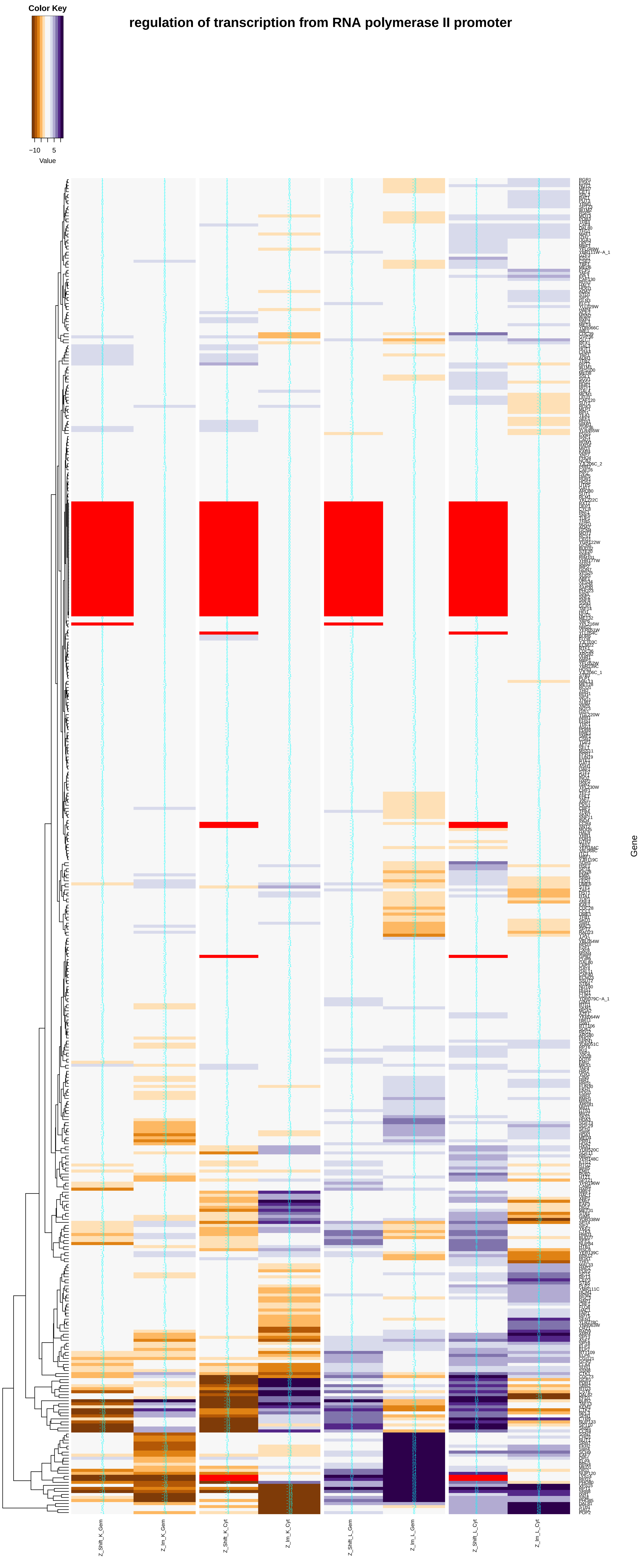

negative regulation of transcription from RNA polymerase II promoter

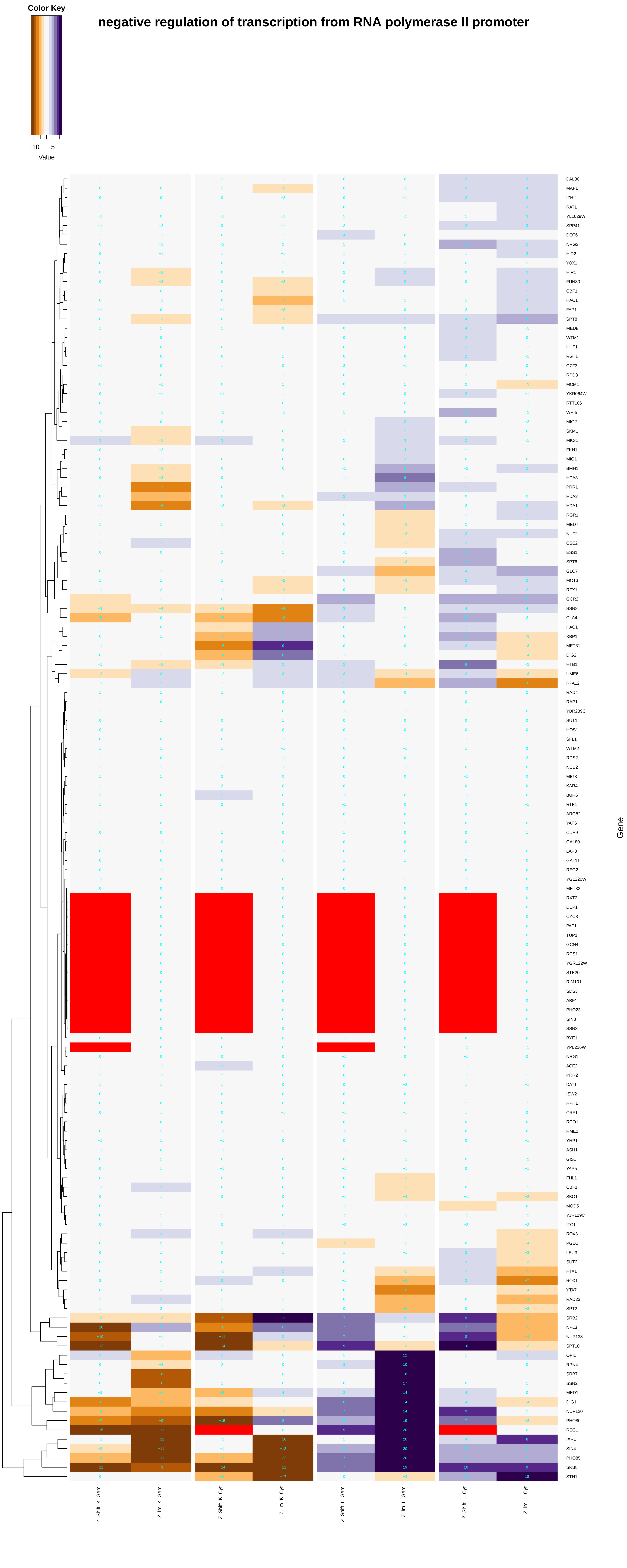

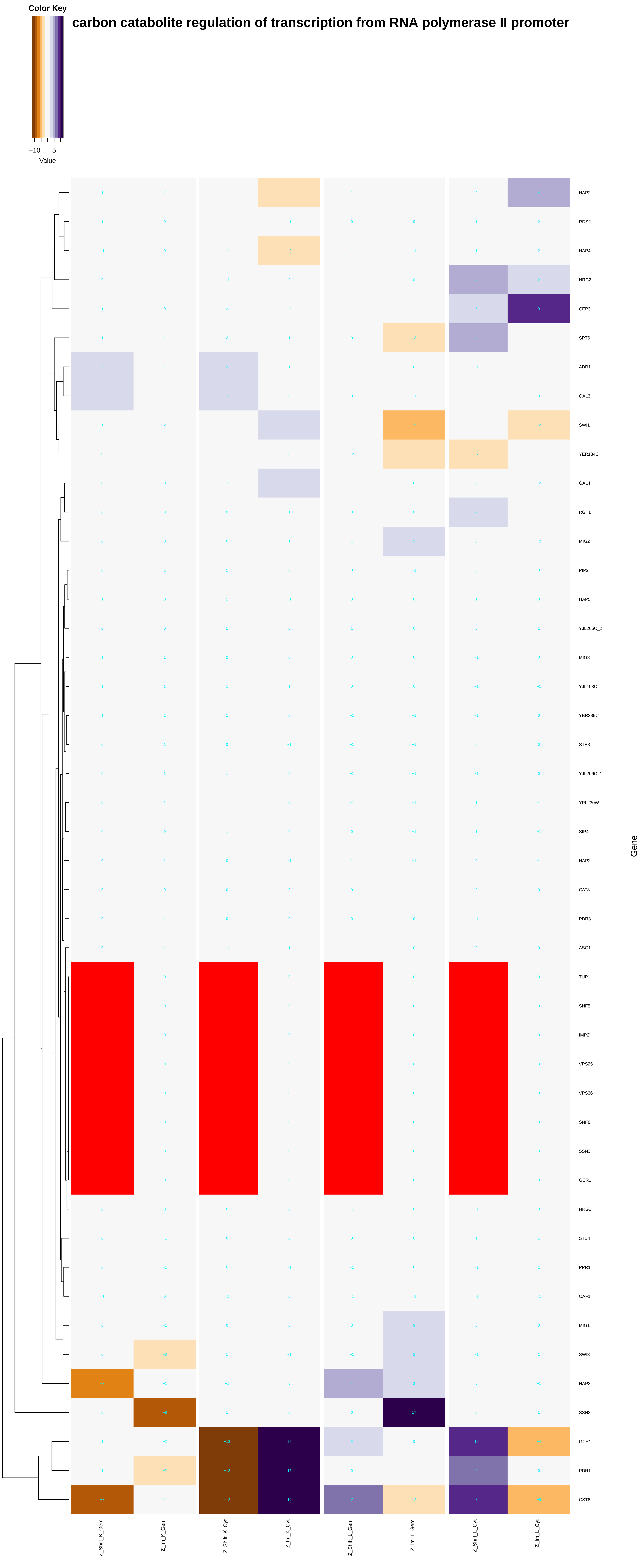

Color Key
−10 5
Value
carbon catabolite regulation of transcription from RNA polymerase II promoter
HAP2
RDS2
HAP4
NRG2
CEP3
SPT6
ADR1
GAL3
SWI1
YER184C
GAL4
RGT1
MIG2
PIP2
HAP5
YJL206C_2
MIG3
YJL103C
YBR239C
STB3
YJL206C_1
YPL230W
SIP4
HAP2
CAT8
PDR3
ASG1
TUP1
SNF5
IMP2'
VPS25
VPS36
SNF8
SSN3
GCR1
NRG1
STB4
PPR1
OAF1
MIG1
SWI3
HAP3
SSN2
GCR1
PDR1
CST6
Gene
Z_Shift_K_Gem
Z_Im_K_Gem
Z_Shift_K_Cyt
Z_Im_K_Cyt
Z_Shift_L_Gem
Z_Im_L_Gem
Z_Shift_L_Cyt
Z_Im_L_Cyt

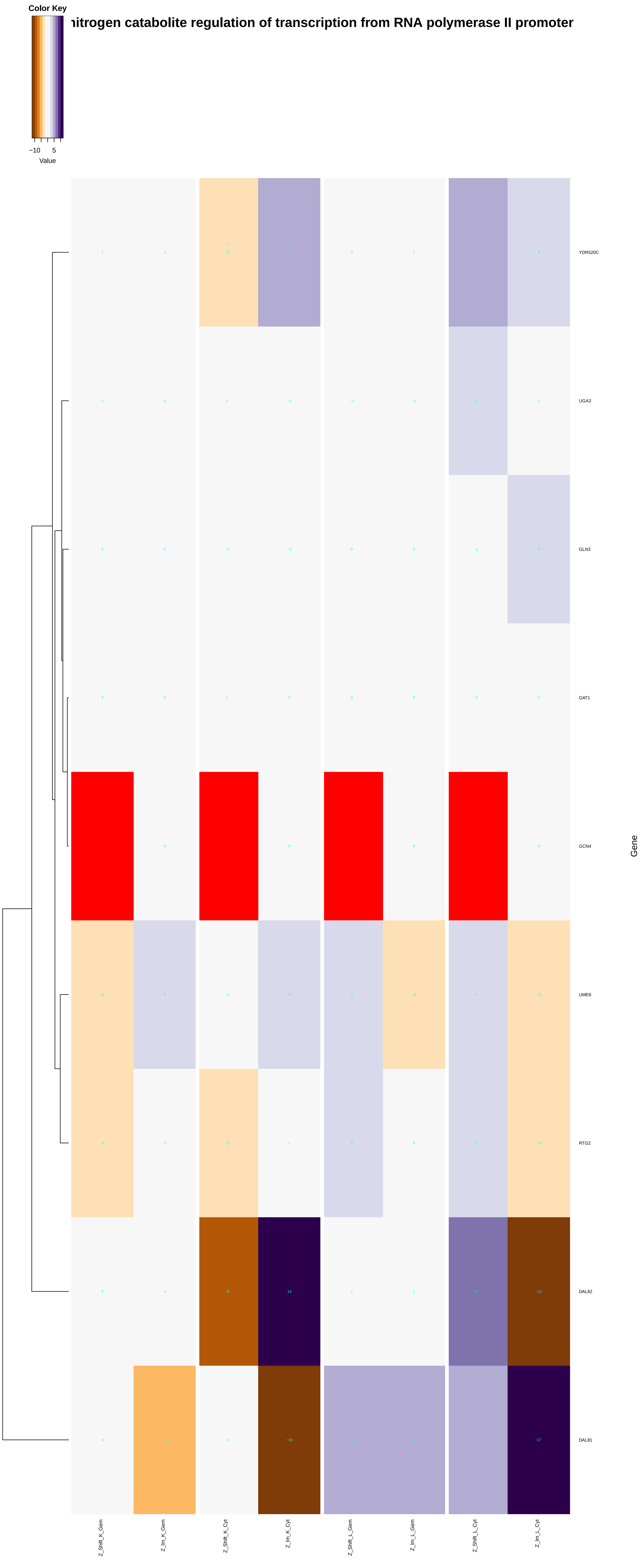

Color Key
−10
5
Value
nitrogen catabolite regulation of transcription from RNA polymerase II promoter
YDR520C
UGA3
GLN3
GAT1
GCN4
UME6
RTG2
DAL82
DAL81
Gene
Z_Shift_K_Gem
Z_Im_K_Gem
Z_Shift_K_Cyt
Z_Im_K_Cyt
Z_Shift_L_Gem
Z_Im_L_Gem
Z_Shift_L_Cyt
Z_Im_L_Cyt

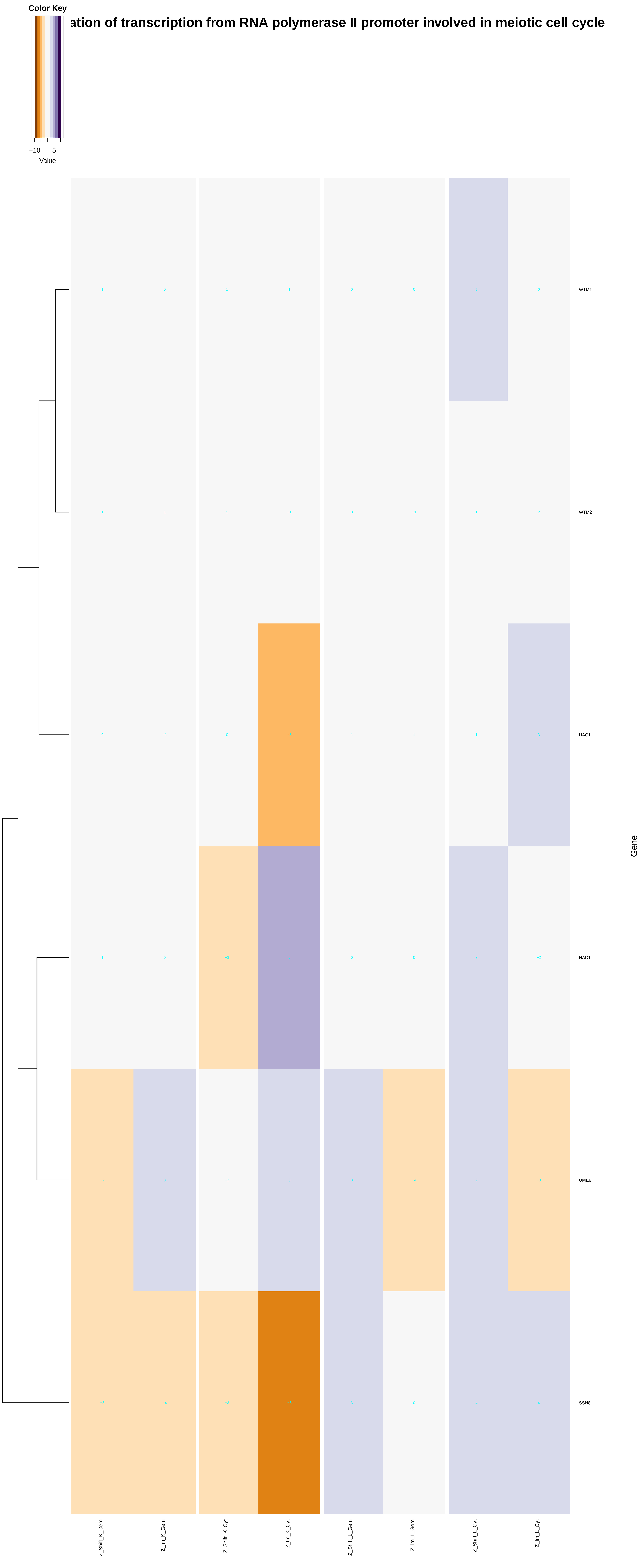
ation of transcription from RNA polymerase II promoter involved in meiotic cell cycle
Color Key
−10
5
Value
WTM1
WTM2
HAC1
HAC1
UME6
SSN8
Gene
Z_Shift_K_Gem
Z_Im_K_Gem
Z_Shift_K_Cyt
Z_Im_K_Cyt
Z_Shift_L_Gem
Z_Im_L_Gem
Z_Shift_L_Cyt
Z_Im_L_Cyt

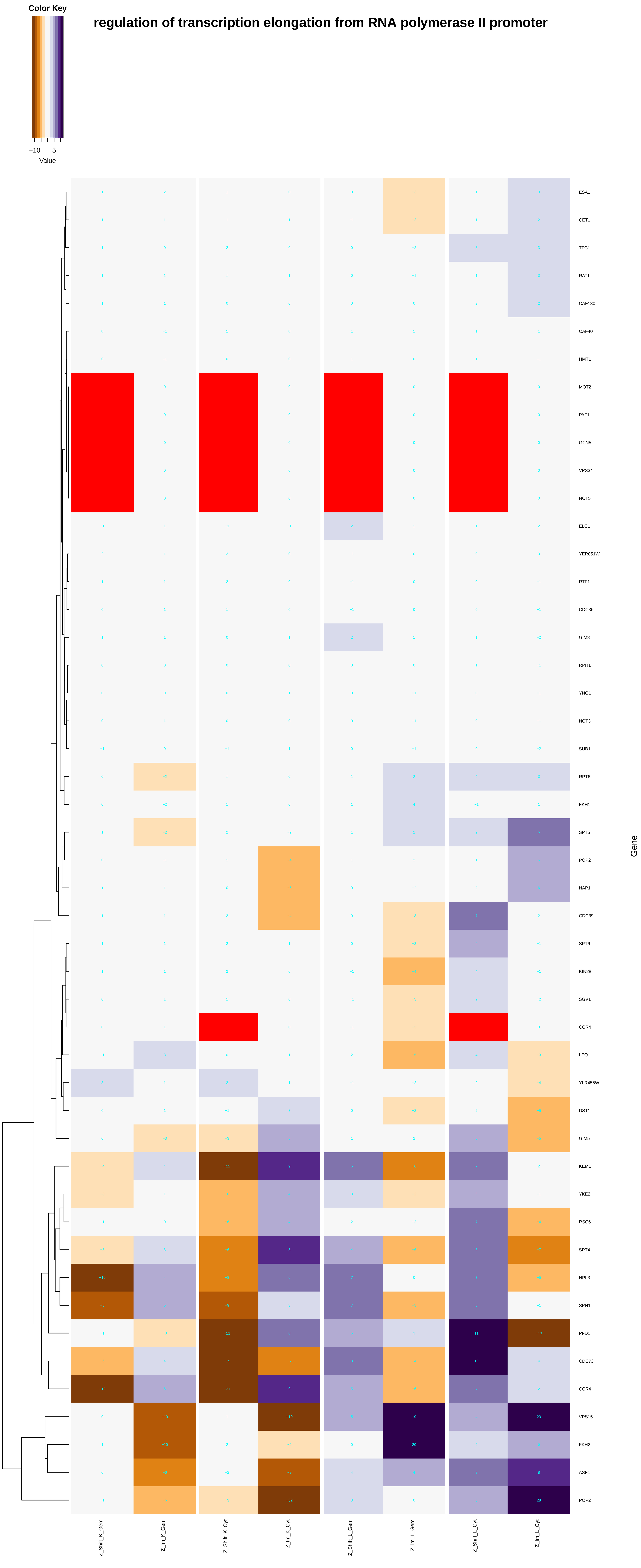

regulation of transcription elongation from RNA polymerase II promoter
Color Key
−10 5
Value
ESA1
CET1
TFG1
RAT1
CAF130
CAF40
HMT1
MOT2
PAF1
GCN5
VPS34
NOT5
ELC1
YER051W
RTF1
CDC36
GIM3
RPH1
YNG1
NOT3
SUB1
RPT6
FKH1
SPT5
POP2
NAP1
CDC39
SPT6
KIN28
SGV1
CCR4
LEO1
YLR455W
DST1
GIM5
KEM1
YKE2
RSC6
SPT4
NPL3
SPN1
PFD1
CDC73
CCR4
VPS15
FKH2
ASF1
POP2
Gene
Z_Shift_K_Gem
Z_Im_K_Gem
Z_Shift_K_Cyt
Z_Im_K_Cyt
Z_Shift_L_Gem
Z_Im_L_Gem
Z_Shift_L_Cyt
Z_Im_L_Cyt

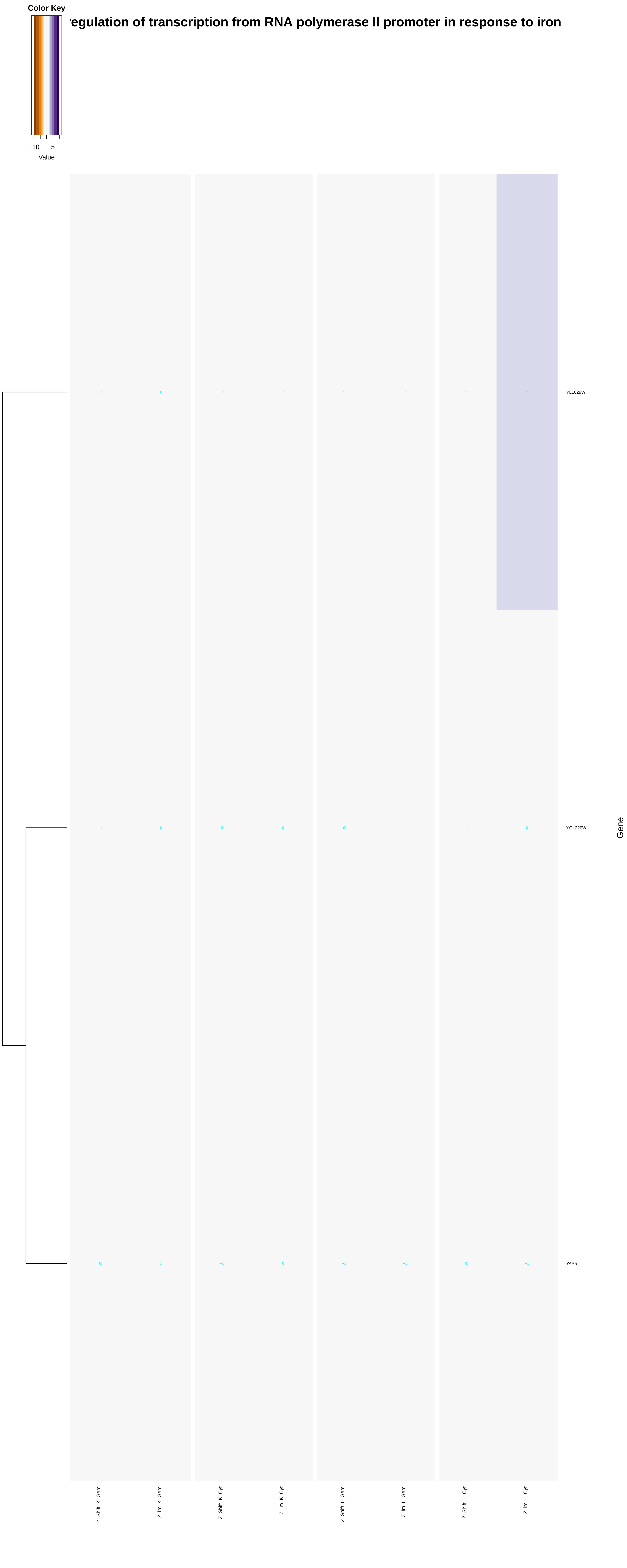

Color Key
−10
5
Value
regulation of transcription from RNA polymerase II promoter in response to iron
−1 0 −1 −1 1 −1 1 3
YLL029W
−1 0 0 0 0 1 −1 0
YGL220W
0 1 −1 0 −1 −1 0 −1
YAP5
Gene
Z_Shift_K_Gem
Z_Im_K_Gem
Z_Shift_K_Cyt
Z_Im_K_Cyt
Z_Shift_L_Gem
Z_Im_L_Gem
Z_Shift_L_Cyt
Z_Im_L_Cyt

# of oligopeptide transport by regulation of transcription from RNA polymerase II promoter

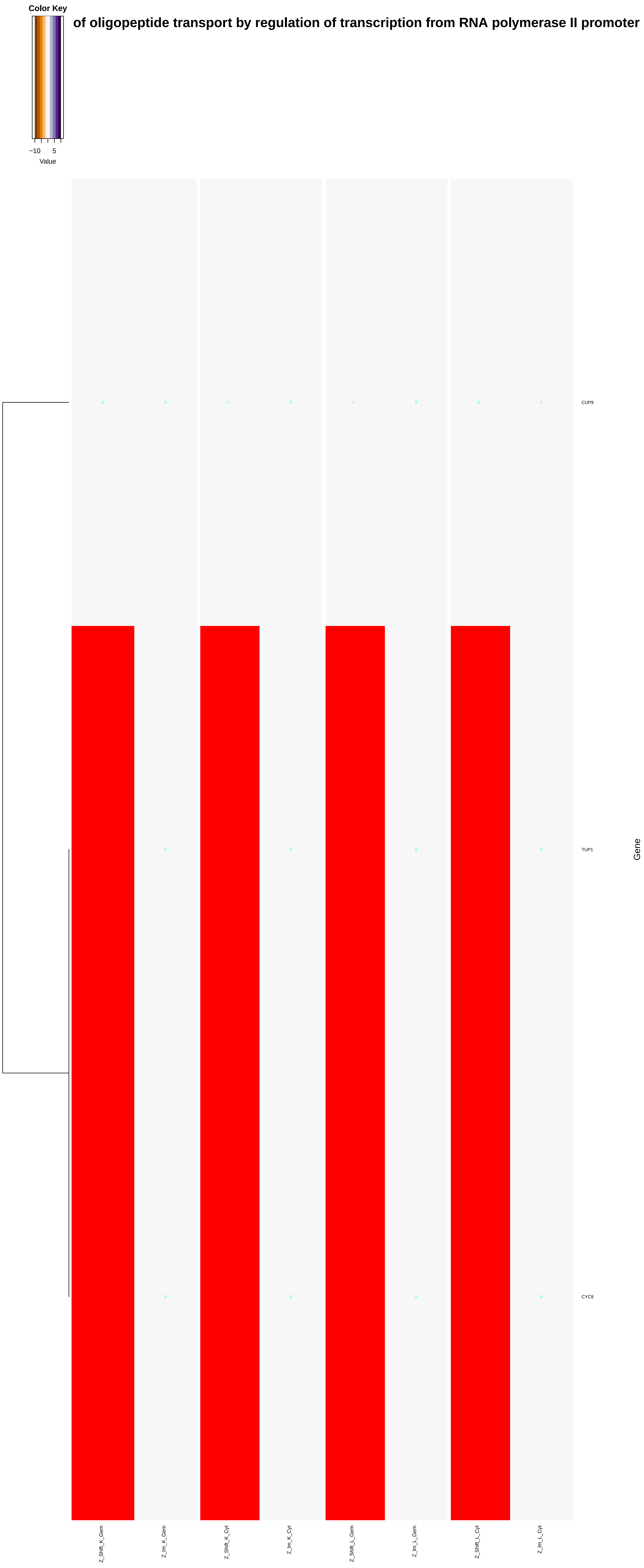

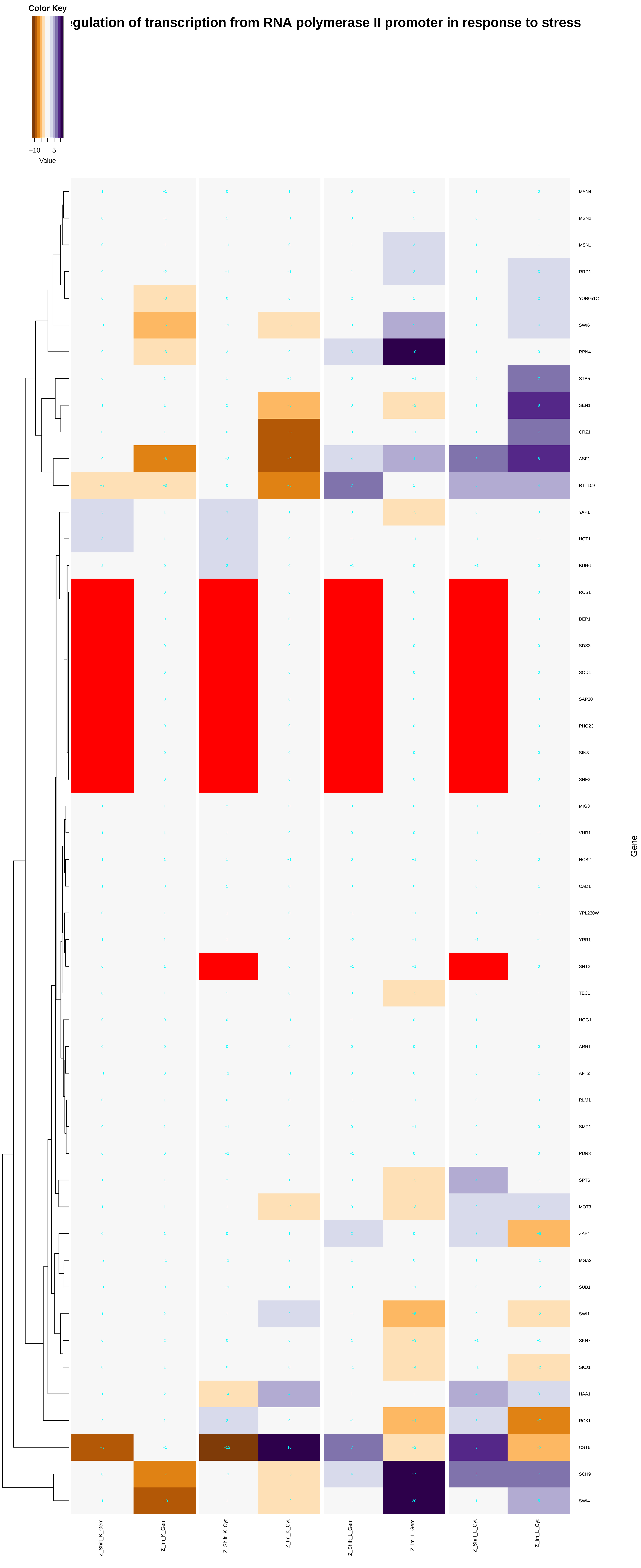
Color Key
−10 5
Value
egulation of transcription from RNA polymerase II promoter in response to stress
MSN4
MSN2
MSN1
RRD1
YOR051C
SWI6
RPN4
STB5
SEN1
CRZ1
ASF1
RTT109
YAP1
HOT1
BUR6
RCS1
DEP1
SDS3
SOD1
SAP30
PHO23
SIN3
SNF2
MIG3
VHR1
NCB2
CAD1
YPL230W
YRR1
SNT2
TEC1
HOG1
ARR1
AFT2
RLM1
SMP1
PDR8
SPT6
MOT3
ZAP1
MGA2
SUB1
SWI1
SKN7
SKO1
HAA1
ROX1
CST6
SCH9
SWI4
Gene
Z_Shift_K_Gem
Z_Im_K_Gem
Z_Shift_K_Cyt
Z_Im_K_Cyt
Z_Shift_L_Gem
Z_Im_L_Gem
Z_Shift_L_Cyt
Z_Im_L_Cyt

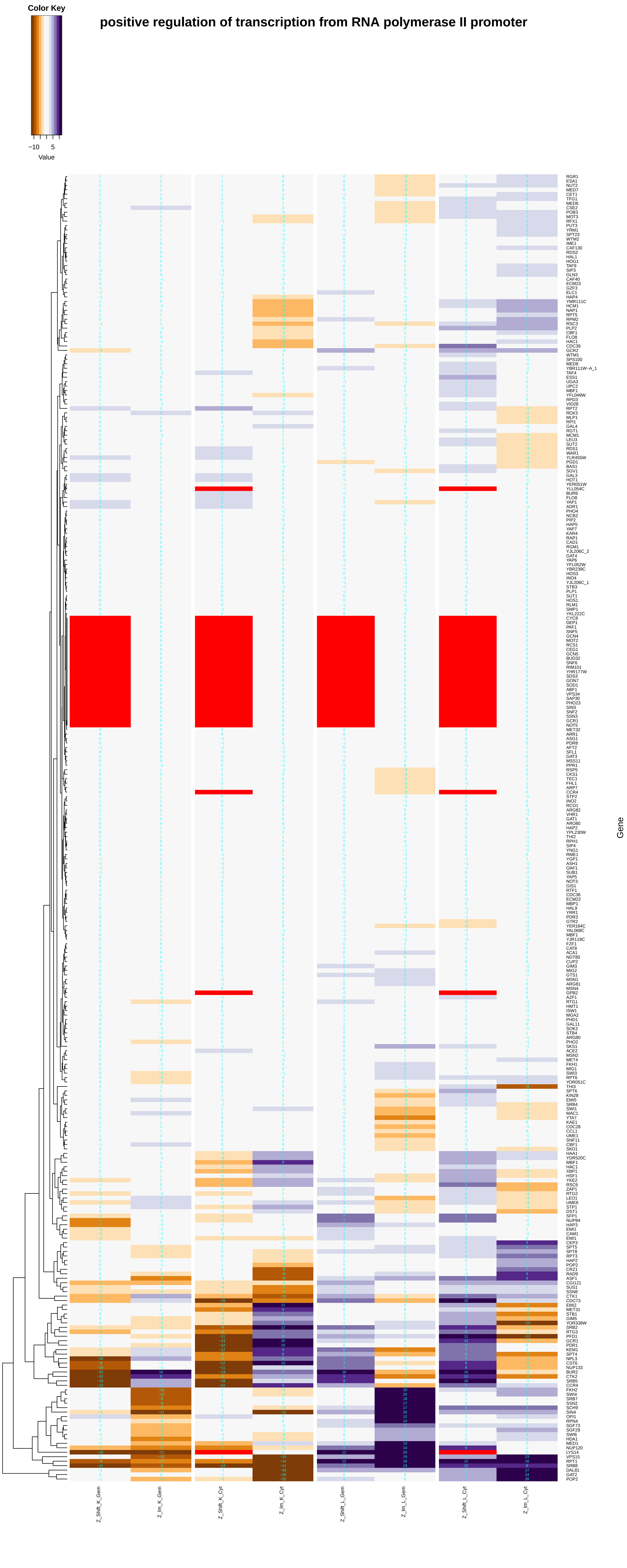
positive regulation of transcription from RNA polymerase II promoter
Color Key
−10
5
Value
Z_Shift_K_Gem
Z_Im_K_Gem
Z_Shift_K_Cyt
Z_Im_K_Cyt
Z_Shift_L_Gem
Z_Im_L_Gem
Z_Shift_L_Cyt
Z_Im_L_Cyt
Gene

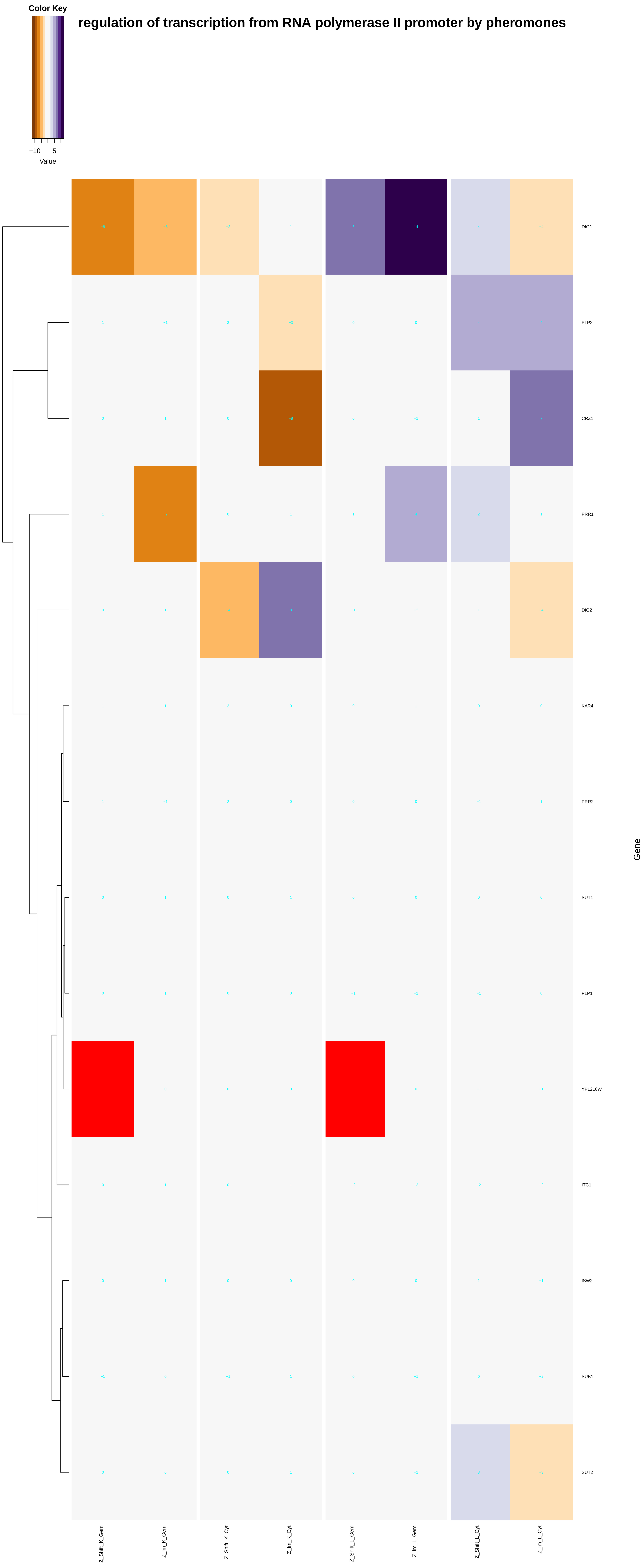
Color Key
−10
5
Value
regulation of transcription from RNA polymerase II promoter by pheromones
DIG1
PLP2
CRZ1
PRR1
DIG2
KAR4
PRR2
SUT1
PLP1
YPL216W
ITC1
ISW2
SUB1
SUT2
Gene
Z_Shift_K_Gem
Z_Im_K_Gem
Z_Shift_K_Cyt
Z_Im_K_Cyt
Z_Shift_L_Gem
Z_Im_L_Gem
Z_Shift_L_Cyt
Z_Im_L_Cyt

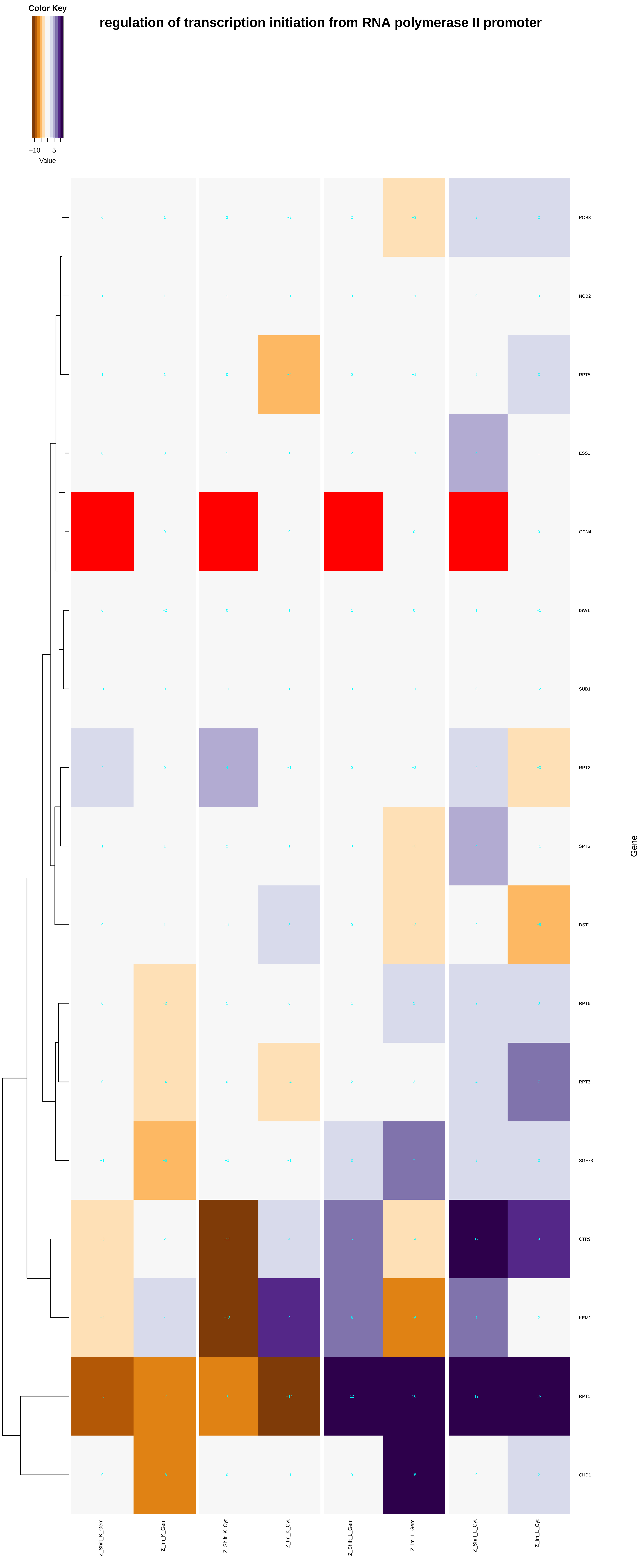
Color Key
−10
5
Value
regulation of transcription initiation from RNA polymerase II promoter
POB3
NCB2
RPT5
ESS1
GCN4
ISW1
SUB1
RPT2
SPT6
DST1
RPT6
RPT3
SGF73
CTR9
KEM1
RPT1
CHD1
Gene
Z_Shift_K_Gem
Z_Im_K_Gem
Z_Shift_K_Cyt
Z_Im_K_Cyt
Z_Shift_L_Gem
Z_Im_L_Gem
Z_Shift_L_Cyt
Z_Im_L_Cyt

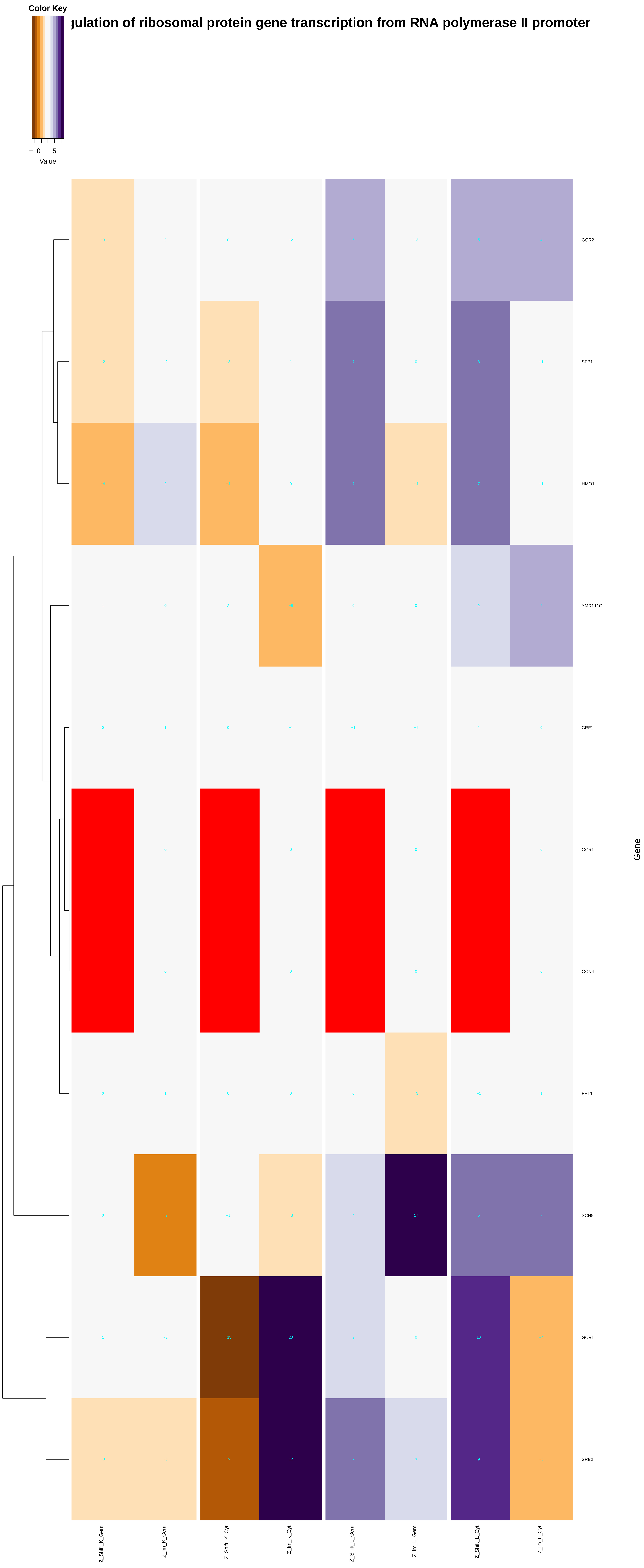

Color Key
−10
5
Value
ulation of ribosomal protein gene transcription from RNA polymerase II promoter
GCR2
SFP1
HMO1
YMR111C
CRF1
GCR1
GCN4
FHL1
SCH9
GCR1
SRB2
Gene
Z_Shift_K_Gem
Z_Im_K_Gem
Z_Shift_K_Cyt
Z_Im_K_Cyt
Z_Shift_L_Gem
Z_Im_L_Gem
Z_Shift_L_Cyt
Z_Im_L_Cyt

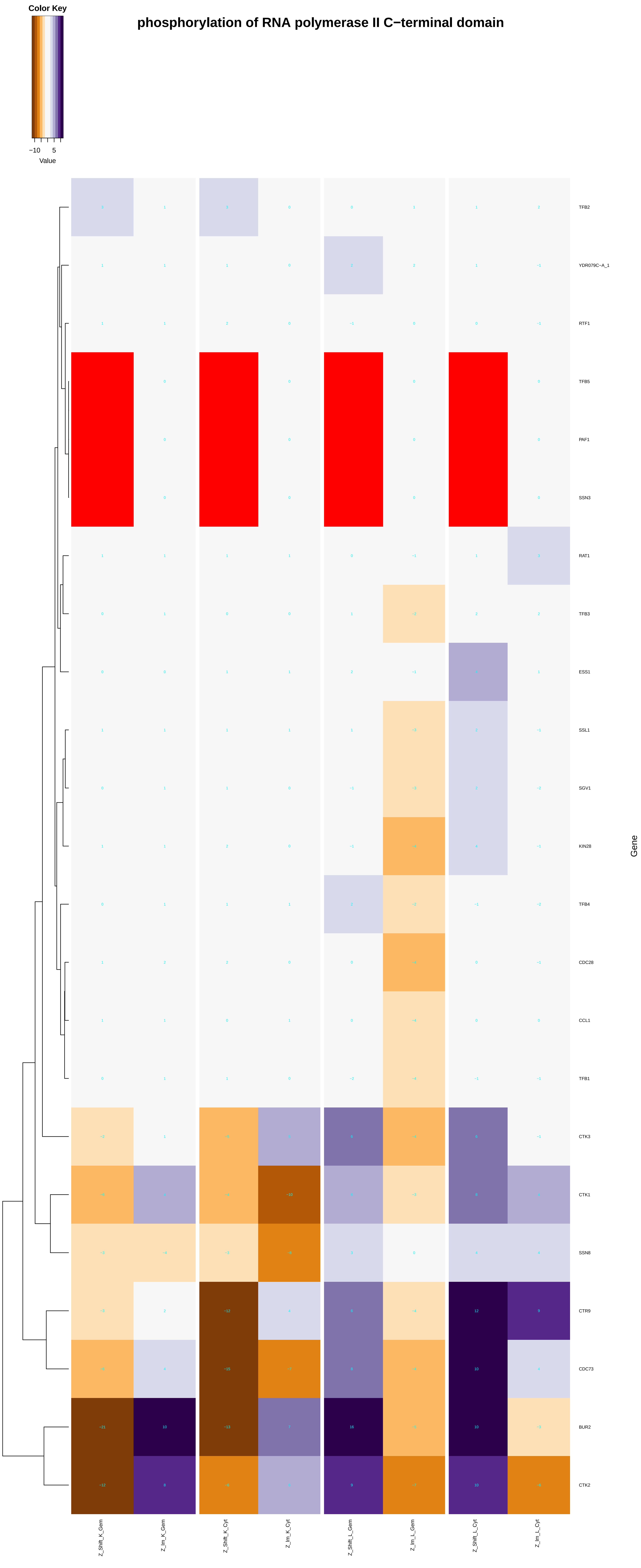
phosphorylation of RNA polymerase II C−terminal domain
Color Key
−10
5
Value
Gene
TFB2
YDR079C−A_1
RTF1
TFB5
PAF1
SSN3
RAT1
TFB3
ESS1
SSL1
SGV1
KIN28
TFB4
CDC28
CCL1
TFB1
CTK3
CTK1
SSN8
CTR9
CDC73
BUR2
CTK2
Z_Shift_K_Gem
Z_Im_K_Gem
Z_Shift_K_Cyt
Z_Im_K_Cyt
Z_Shift_L_Gem
Z_Im_L_Gem
Z_Shift_L_Cyt
Z_Im_L_Cyt

dephosphorylation of RNA polymerase II C−terminal domain

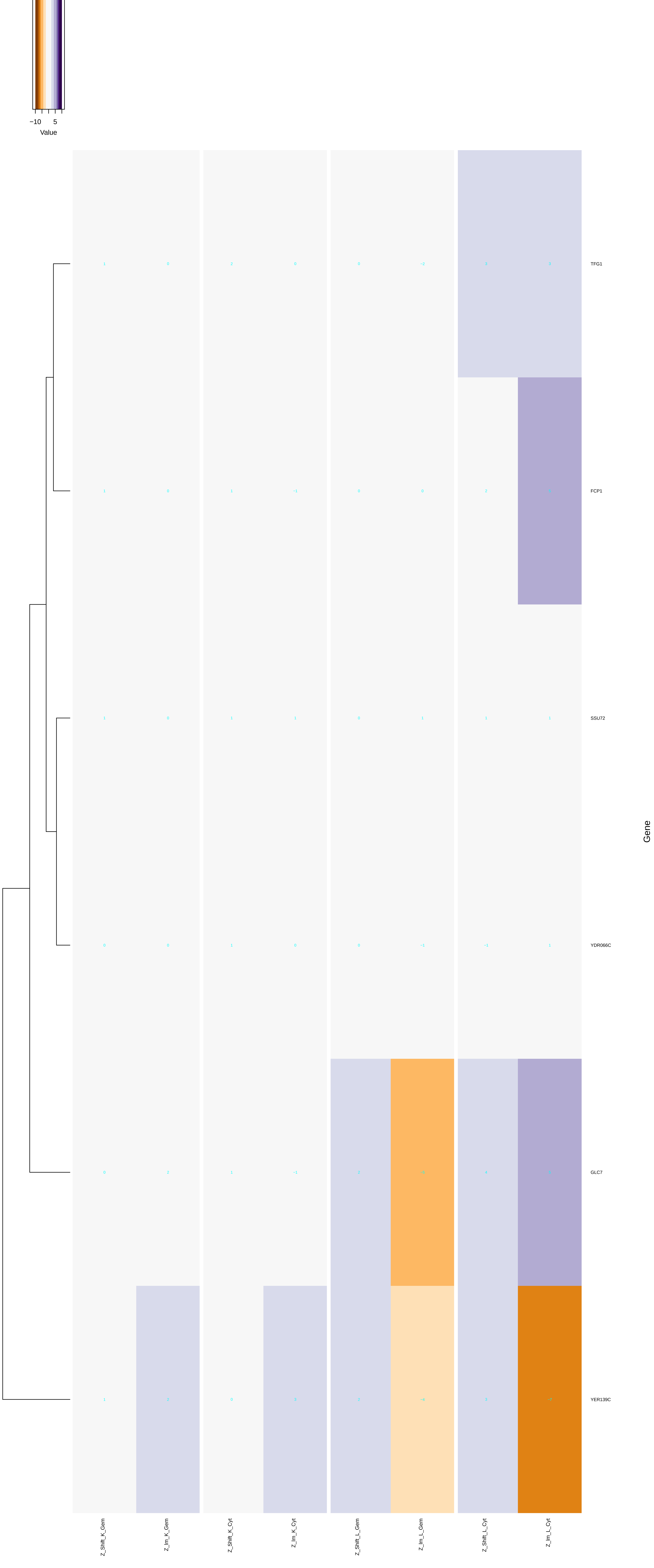

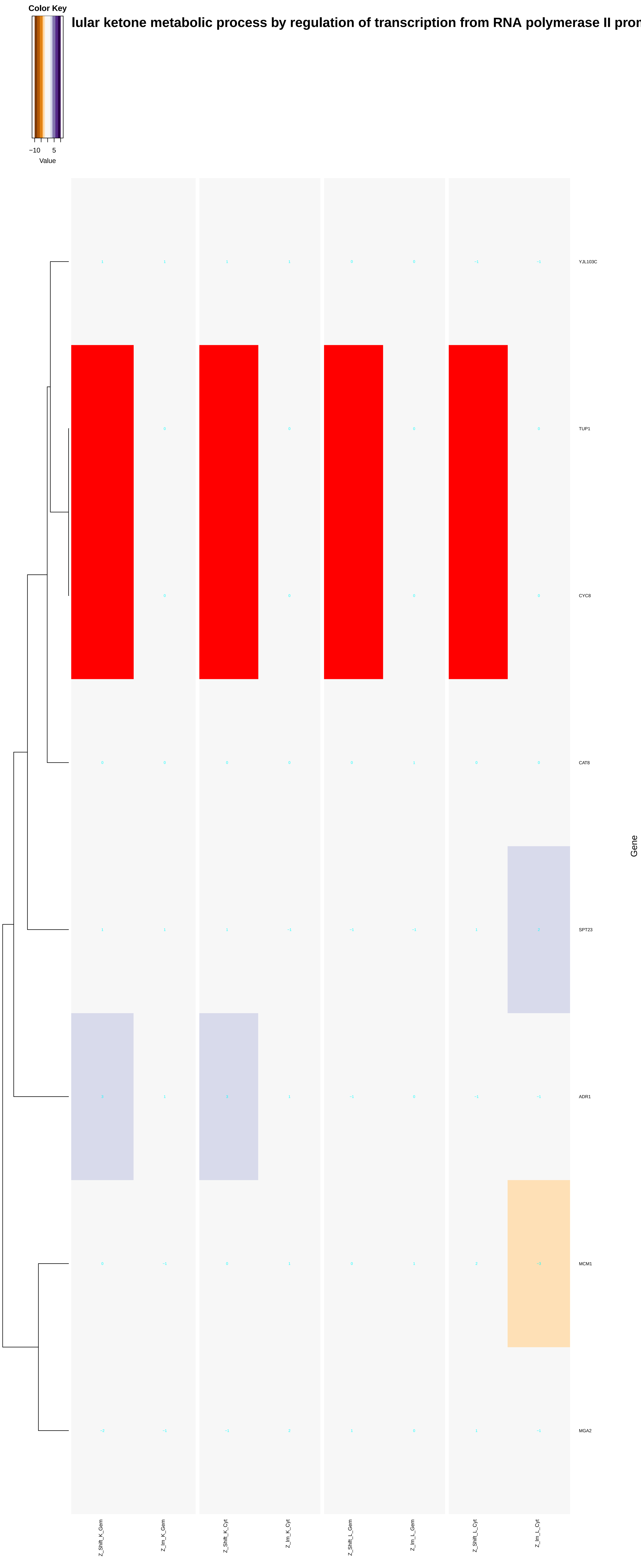

Color Key
−10
5
Value
lular ketone metabolic process by regulation of transcription from RNA polymerase II pror
YJL103C
TUP1
CYC8
CAT8
SPT23
ADR1
MCM1
MGA2
Gene
Z_Shift_K_Gem
Z_Im_K_Gem
Z_Shift_K_Cyt
Z_Im_K_Cyt
Z_Shift_L_Gem
Z_Im_L_Gem
Z_Shift_L_Cyt
Z_Im_L_Cyt

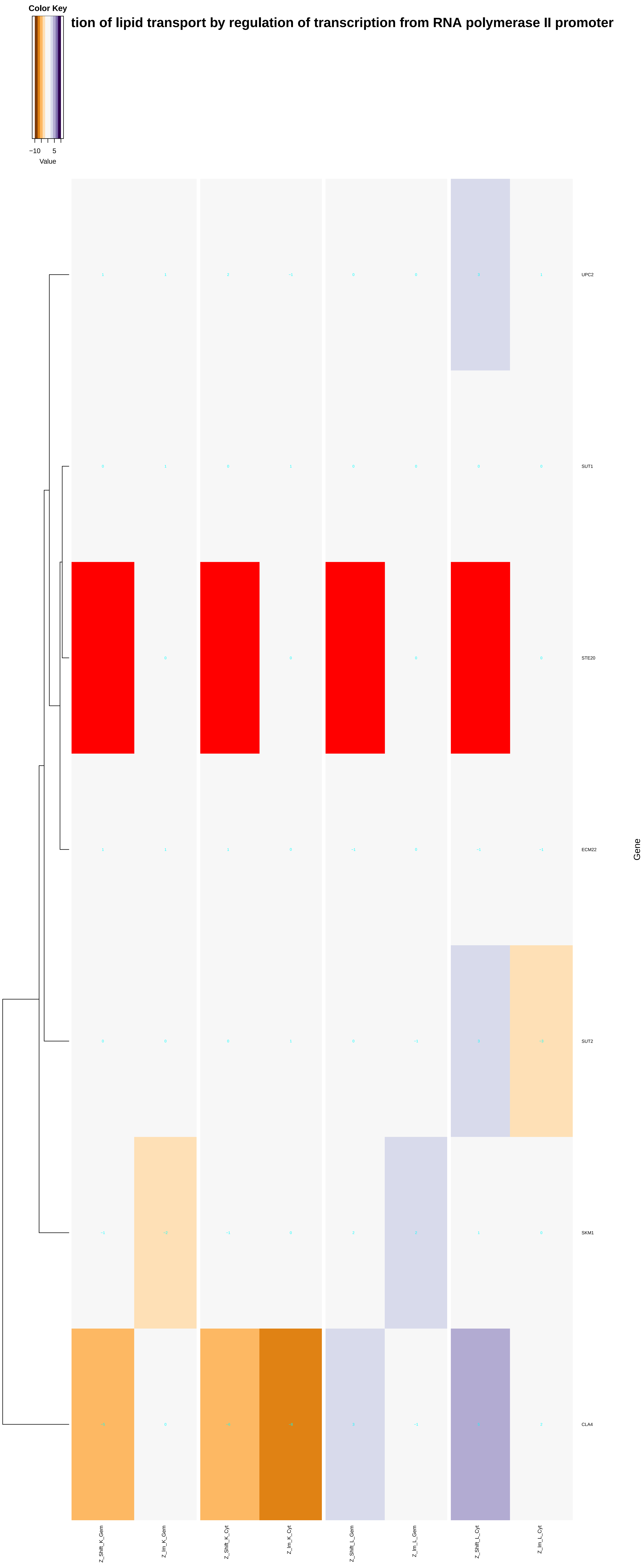

Color Key
−10
5
Value
tion of lipid transport by regulation of transcription from RNA polymerase II promoter
UPC2
SUT1
STE20
ECM22
SUT2
SKM1
CLA4
Gene
Z_Shift_K_Gem
Z_Im_K_Gem
Z_Shift_K_Cyt
Z_Im_K_Cyt
Z_Shift_L_Gem
Z_Im_L_Gem
Z_Shift_L_Cyt
Z_Im_L_Cyt

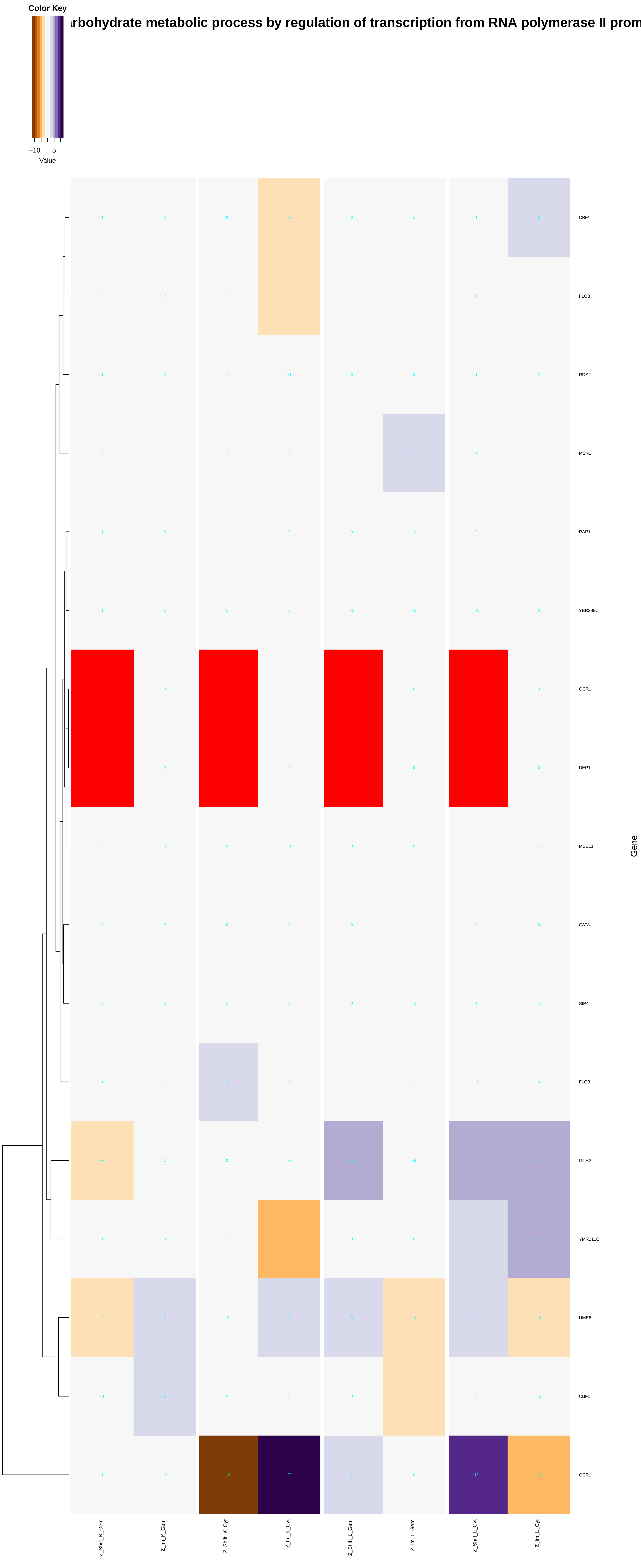

Color Key
−10
5
Value
rbohydrate metabolic process by regulation of transcription from RNA polymerase II prom
CBF1
FLO8
RDS2
MSN1
RAP1
YBR239C
GCR1
DEP1
MSS11
CAT8
SIP4
FLO8
GCR2
YMR111C
UME6
CBF1
GCR1
Gene
Z_Shift_K_Gem
Z_Im_K_Gem
Z_Shift_K_Cyt
Z_Im_K_Cyt
Z_Shift_L_Gem
Z_Im_L_Gem
Z_Shift_L_Cyt
Z_Im_L_Cyt

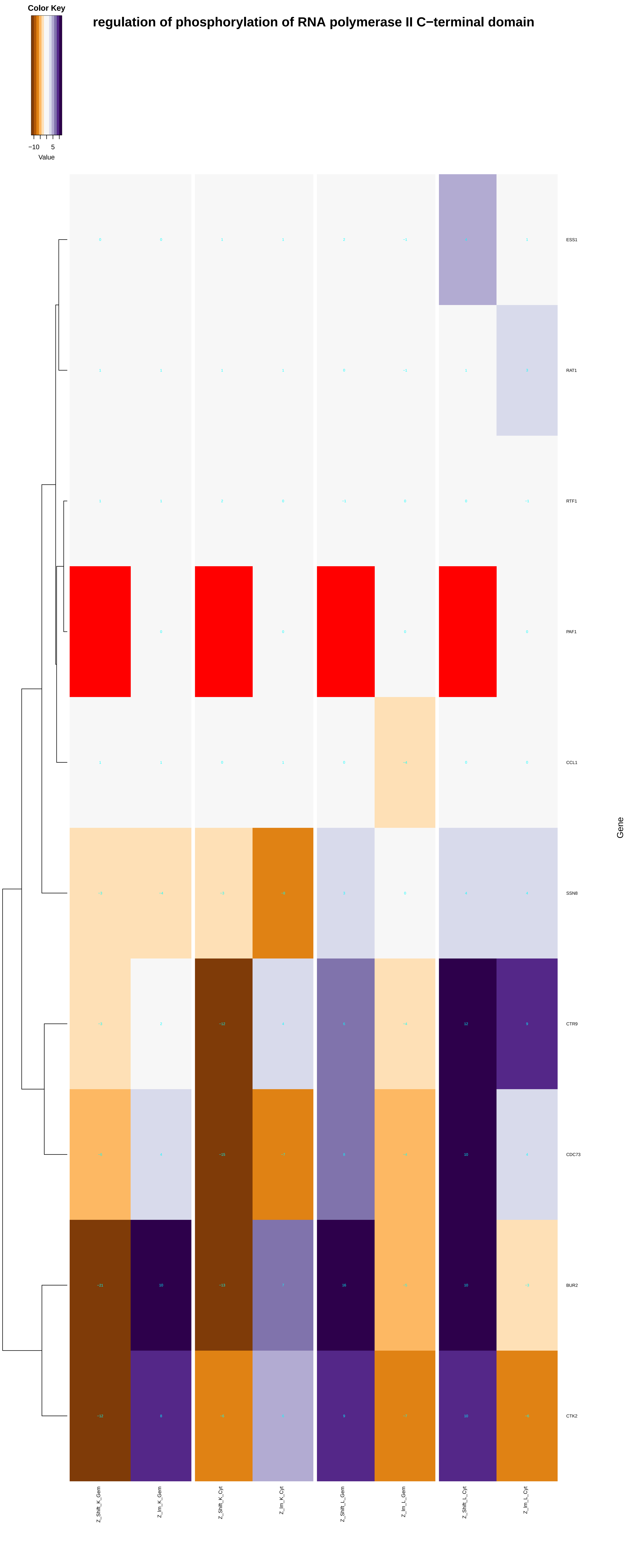

Color Key
−10 5
Value
regulation of phosphorylation of RNA polymerase II C−terminal domain
ESS1
RAT1
RTF1
PAF1
CCL1
SSN8
CTR9
CDC73
BUR2
CTK2
Gene
Z_Shift_K_Gem
Z_Im_K_Gem
Z_Shift_K_Cyt
Z_Im_K_Cyt
Z_Shift_L_Gem
Z_Im_L_Gem
Z_Shift_L_Cyt
Z_Im_L_Cyt

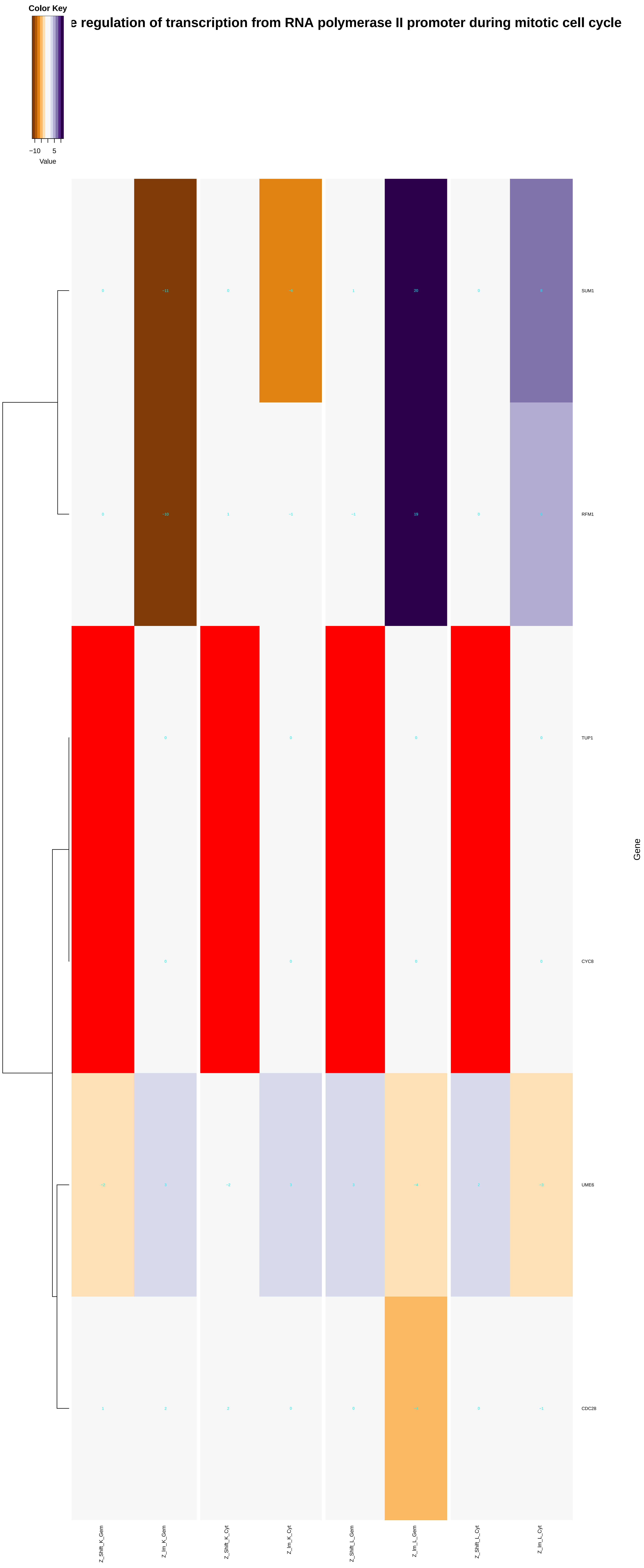

Color Key
−10 5
Value
e regulation of transcription from RNA polymerase II promoter during mitotic cell cycle
SUM1
RFM1
TUP1
CYC8
UME6
CDC28
Gene
Z_Shift_K_Gem
Z_Im_K_Gem
Z_Shift_K_Cyt
Z_Im_K_Cyt
Z_Shift_L_Gem
Z_Im_L_Gem
Z_Shift_L_Cyt
Z_Im_L_Cyt

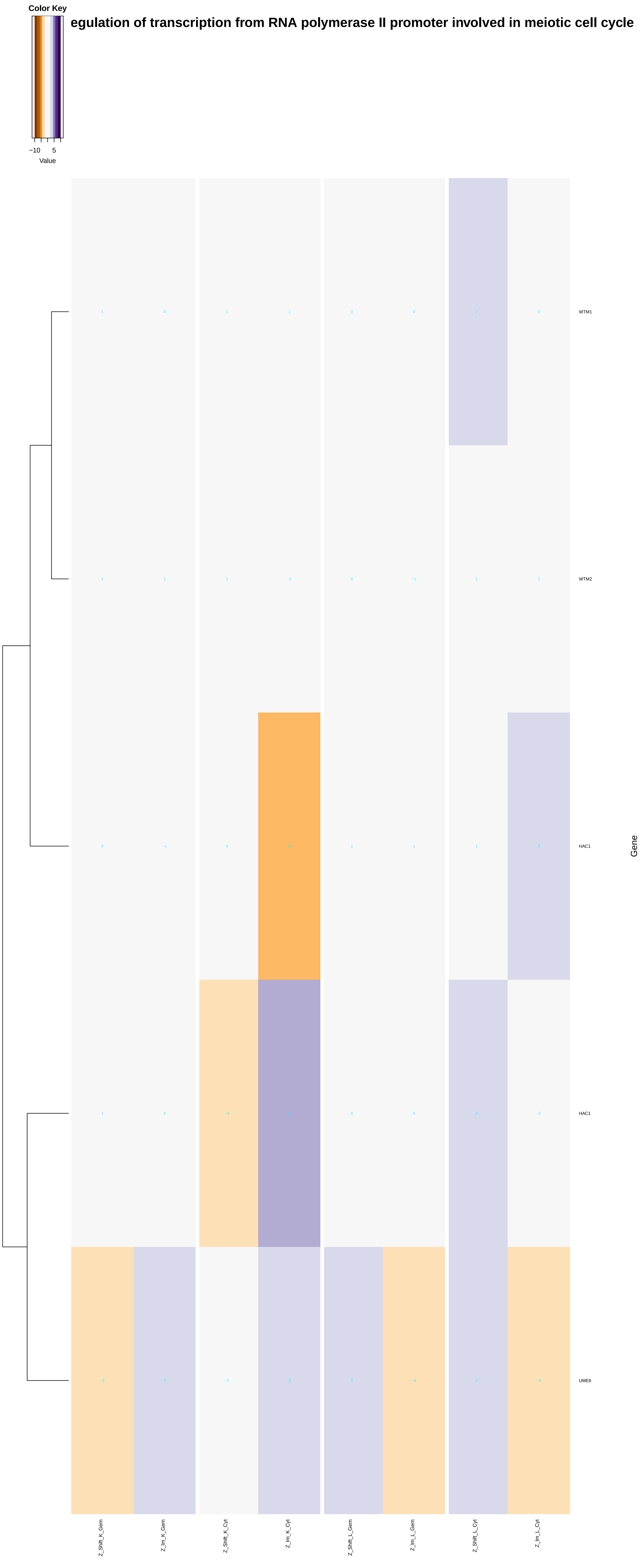
egulation of transcription from RNA polymerase II promoter involved in meiotic cell cycle
Color Key
−10
5
Value
WTM1
WTM2
HAC1
HAC1
UME6
Gene
Z_Shift_K_Gem
Z_Im_K_Gem
Z_Shift_K_Cyt
Z_Im_K_Cyt
Z_Shift_L_Gem
Z_Im_L_Gem
Z_Shift_L_Cyt
Z_Im_L_Cyt

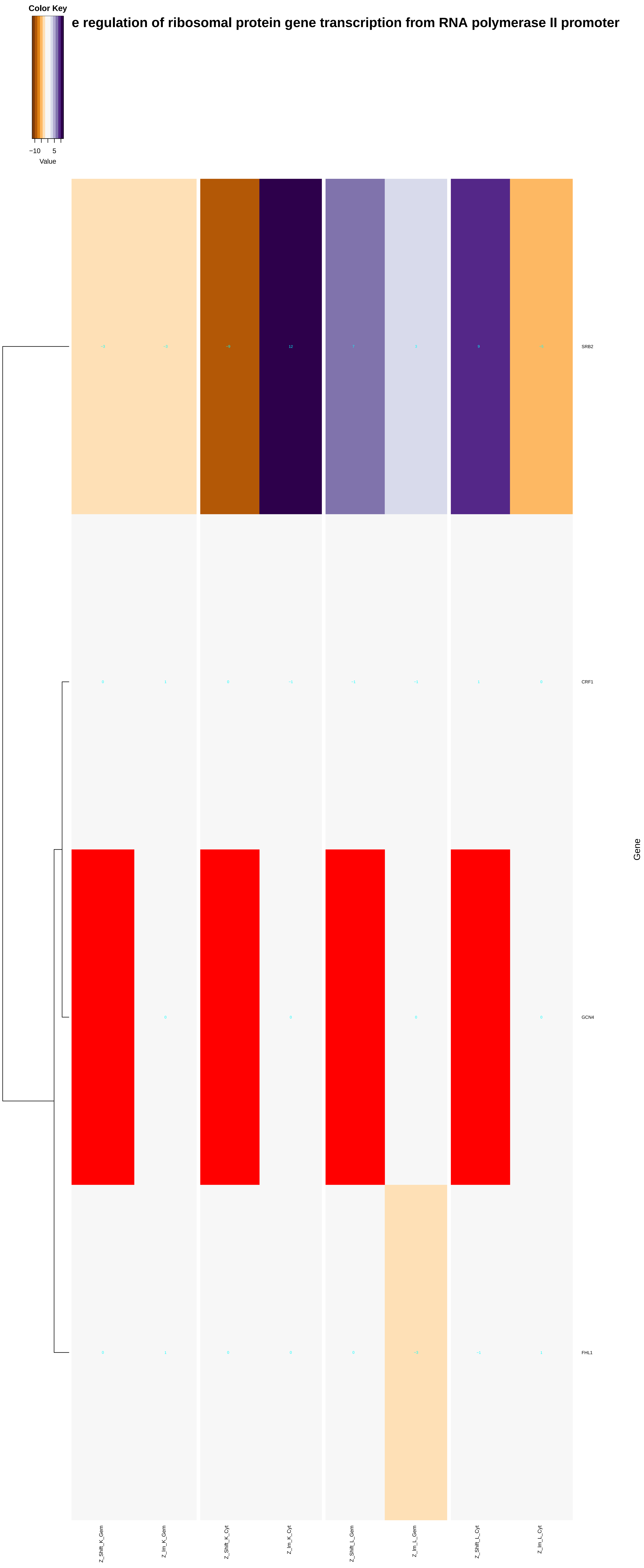
Color Key
−10
5
Value
e regulation of ribosomal protein gene transcription from RNA polymerase II promoter
SRB2
CRF1
GCN4
FHL1
Gene
Z_Shift_K_Gem
Z_Im_K_Gem
Z_Shift_K_Cyt
Z_Im_K_Cyt
Z_Shift_L_Gem
Z_Im_L_Gem
Z_Shift_L_Cyt
Z_Im_L_Cyt

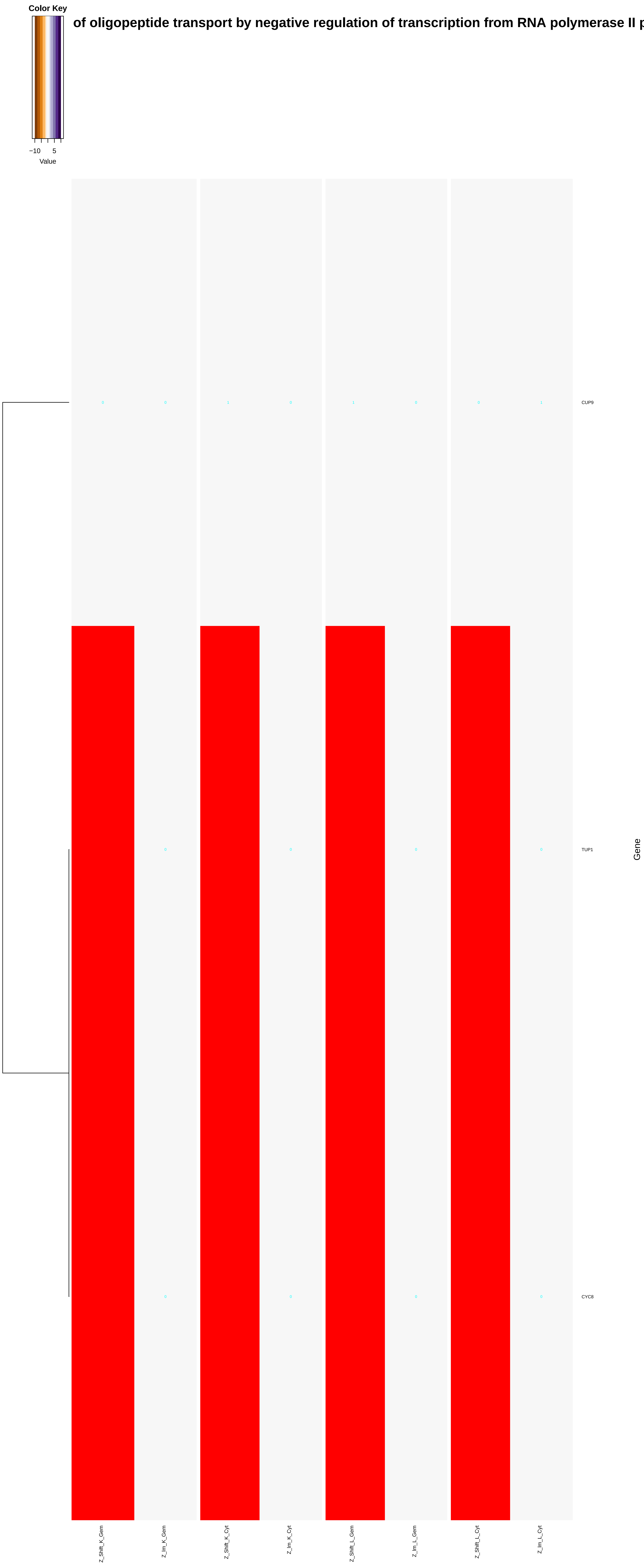
Color Key
−10
5
Value
of oligopeptide transport by negative regulation of transcription from RNA polymerase II p
CUP9
TUP1
CYC8
Gene
Z_Shift_K_Gem
Z_Im_K_Gem
Z_Shift_K_Cyt
Z_Im_K_Cyt
Z_Shift_L_Gem
Z_Im_L_Gem
Z_Shift_L_Cyt
Z_Im_L_Cyt

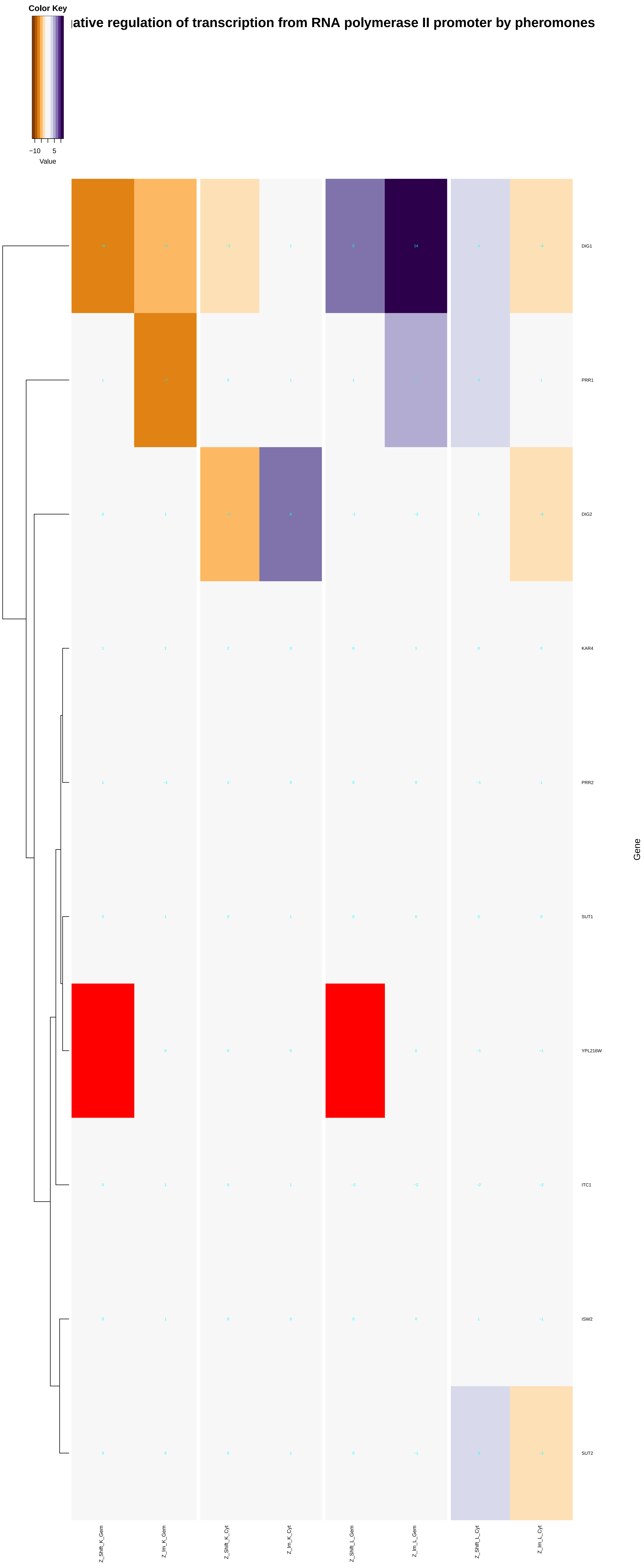
Color Key
−10
5
Value
ative regulation of transcription from RNA polymerase II promoter by pheromones
DIG1
PRR1
DIG2
KAR4
PRR2
SUT1
YPL216W
ITC1
ISW2
SUT2
Gene
Z_Shift_K_Gem
Z_Im_K_Gem
Z_Shift_K_Cyt
Z_Im_K_Cyt
Z_Shift_L_Gem
Z_Im_L_Gem
Z_Shift_L_Cyt
Z_Im_L_Cyt

transfer RNA gene−mediated silencing

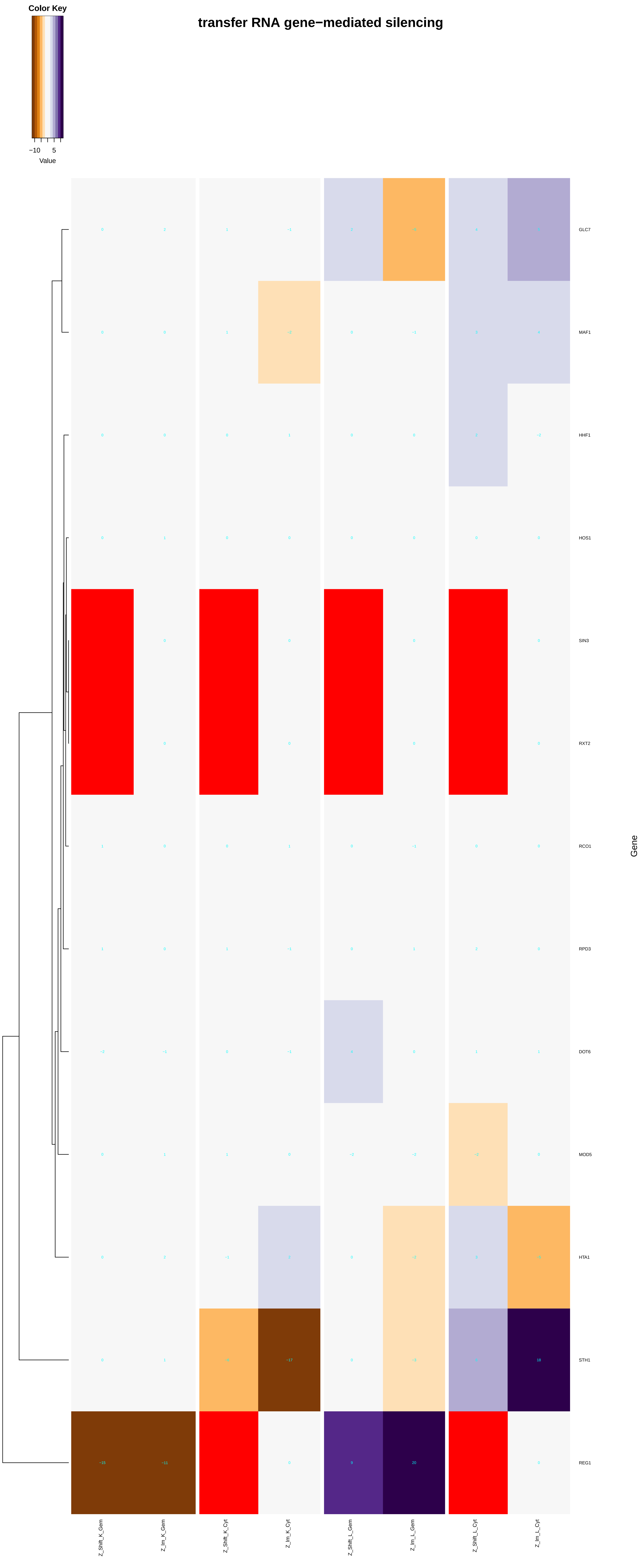

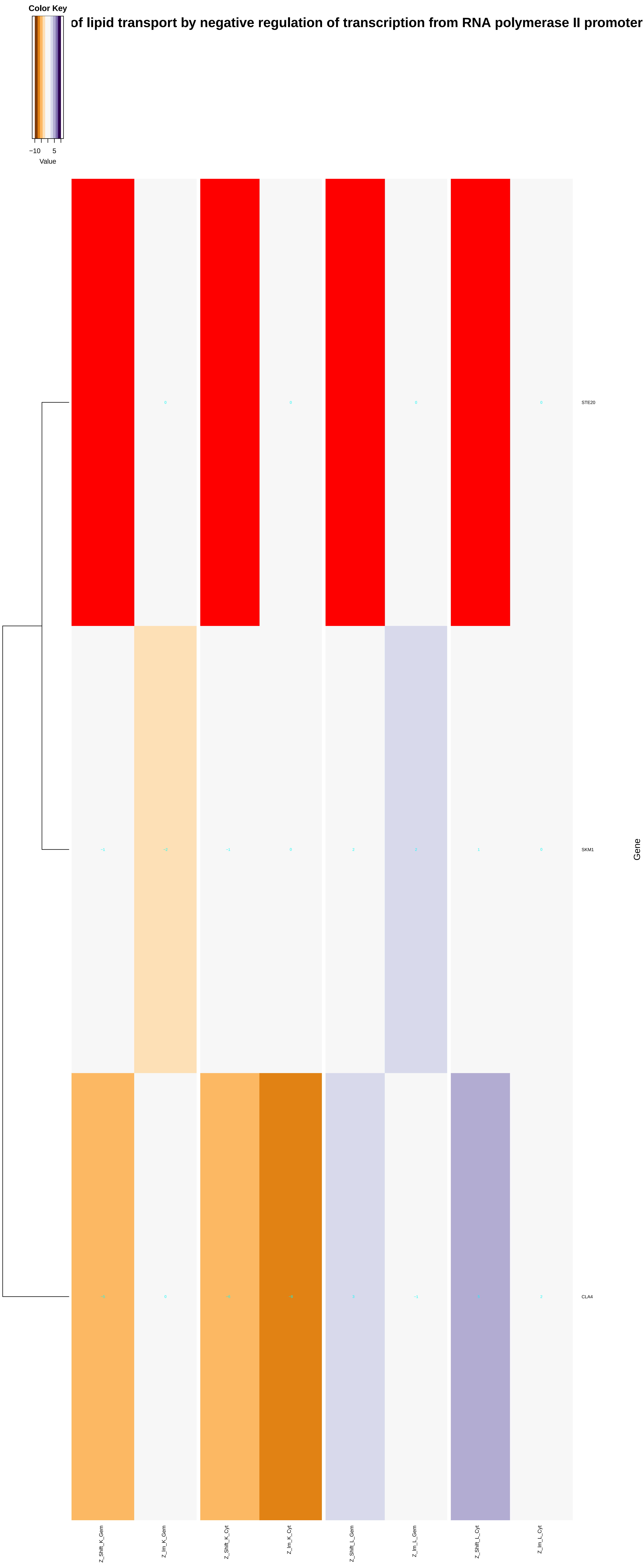
of lipid transport by negative regulation of transcription from RNA polymerase II promoter
Color Key
−10 5
Value
STE20
SKM1
CLA4
Gene
Z_Shift_K_Gem
Z_Im_K_Gem
Z_Shift_K_Cyt
Z_Im_K_Cyt
Z_Shift_L_Gem
Z_Im_L_Gem
Z_Shift_L_Cyt
Z_Im_L_Cyt

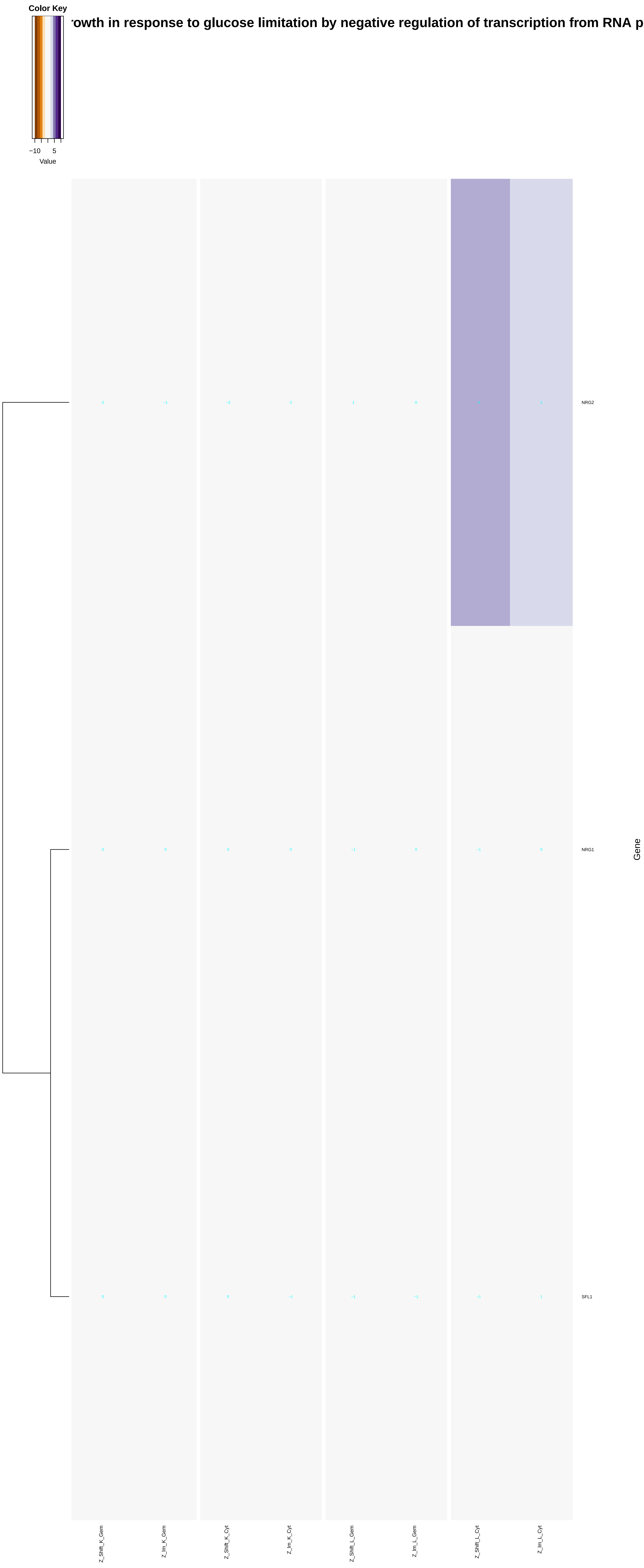
Color Key
−10
5
Value
rowth in response to glucose limitation by negative regulation of transcription from RNA p
NRG2
NRG1
SFL1
Gene
Z_Shift_K_Gem
Z_Im_K_Gem
Z_Shift_K_Cyt
Z_Im_K_Cyt
Z_Shift_L_Gem
Z_Im_L_Gem
Z_Shift_L_Cyt
Z_Im_L_Cyt

regulation of transcription from RNA polymerase II promoter by glucose

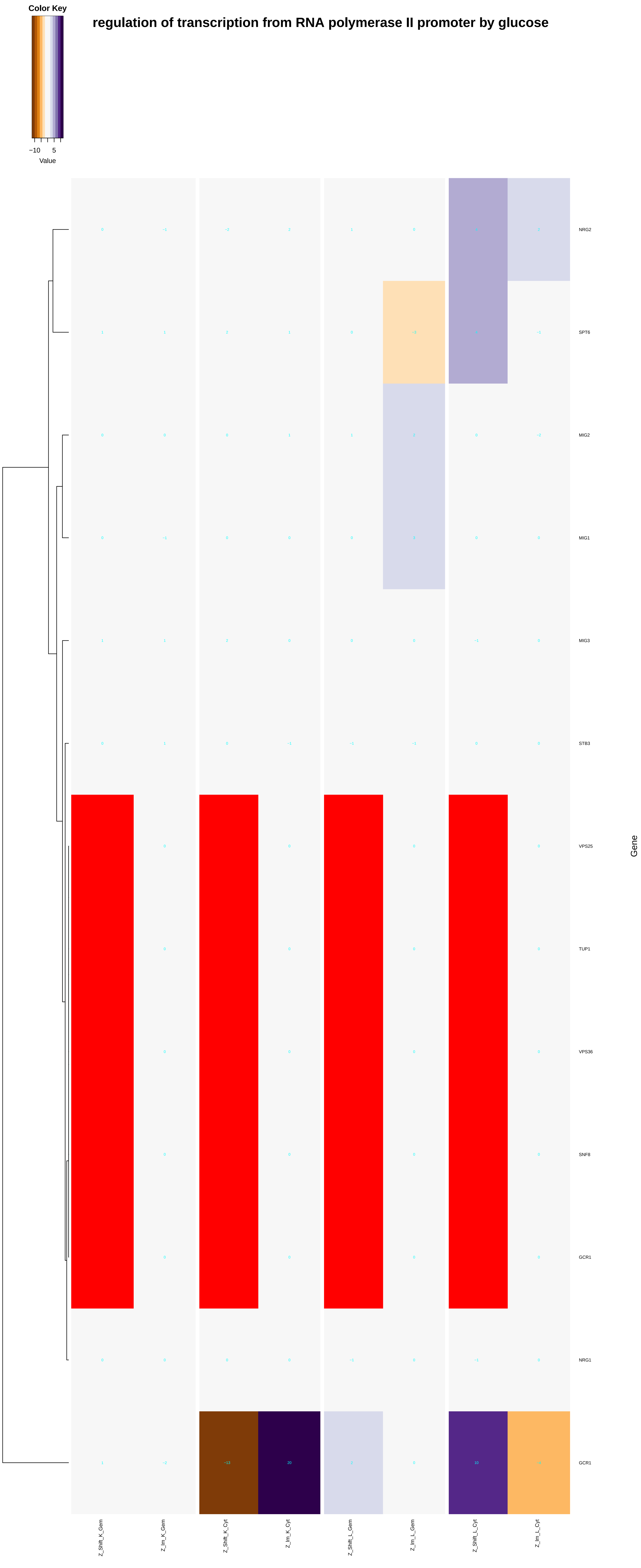

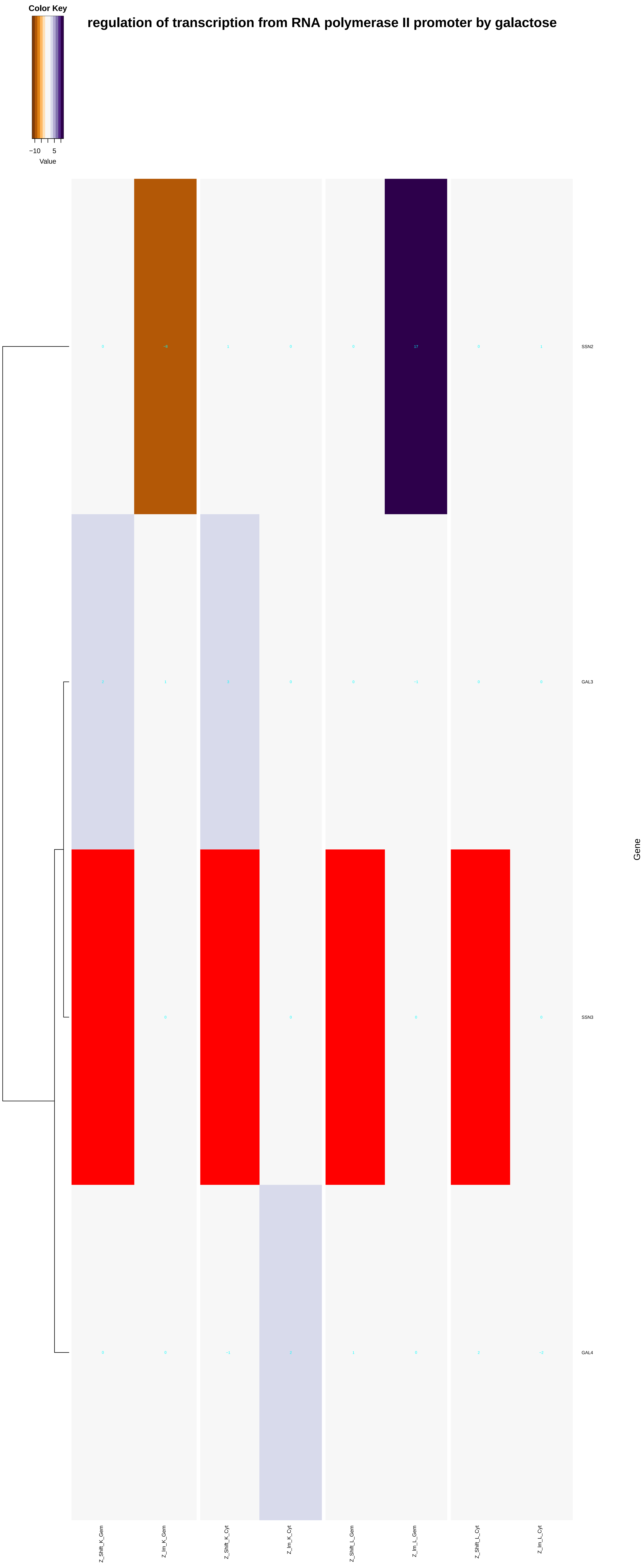
Color Key
−10
5
Value
regulation of transcription from RNA polymerase II promoter by galactose
SSN2
GAL3
SSN3
GAL4
Gene
Z_Shift_K_Gem
Z_Im_K_Gem
Z_Shift_K_Cyt
Z_Im_K_Cyt
Z_Shift_L_Gem
Z_Im_L_Gem
Z_Shift_L_Cyt
Z_Im_L_Cyt

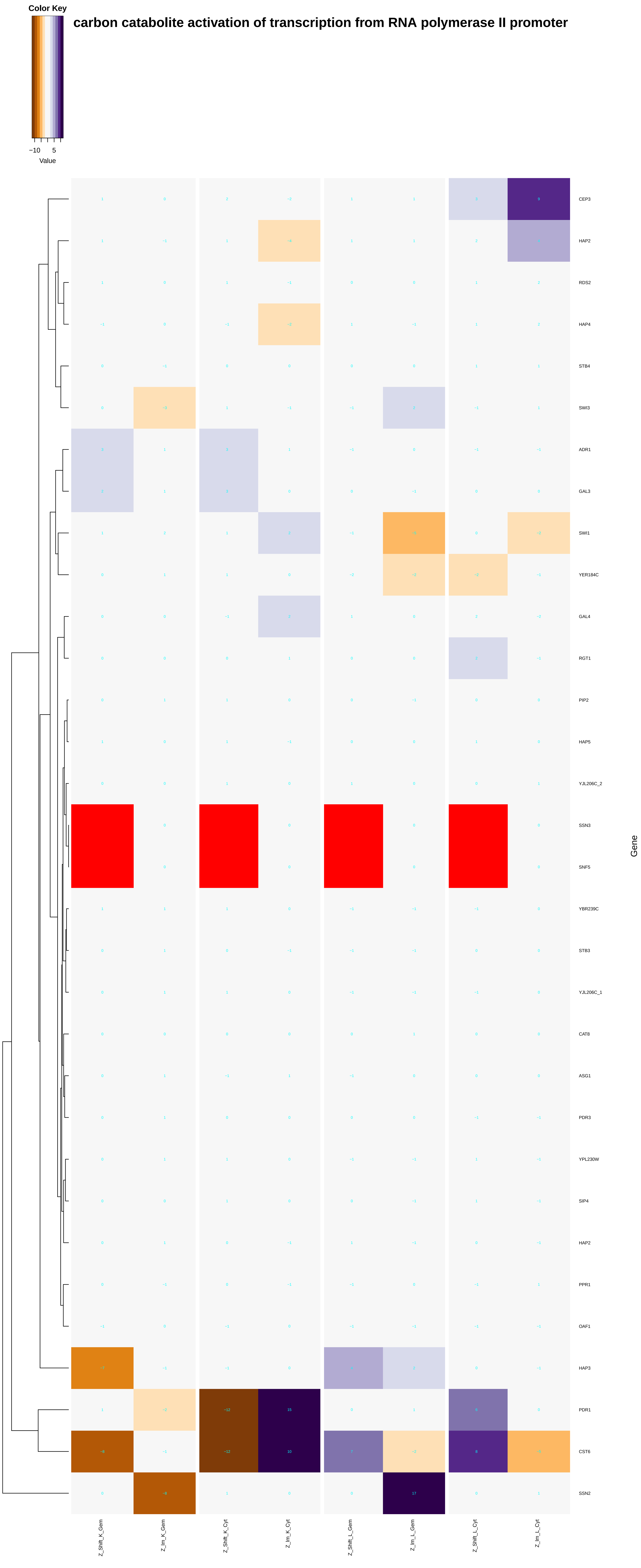

Color Key
−10
5
Value
carbon catabolite activation of transcription from RNA polymerase II promoter
CEP3
HAP2
RDS2
HAP4
STB4
SWI3
ADR1
GAL3
SWI1
YER184C
GAL4
RGT1
PIP2
HAP5
YJL206C_2
SSN3
SNF5
YBR239C
STB3
YJL206C_1
CAT8
ASG1
PDR3
YPL230W
SIP4
HAP2
PPR1
OAF1
HAP3
PDR1
CST6
SSN2
Gene
Z_Shift_K_Gem
Z_Im_K_Gem
Z_Shift_K_Cyt
Z_Im_K_Cyt
Z_Shift_L_Gem
Z_Im_L_Gem
Z_Shift_L_Cyt
Z_Im_L_Cyt

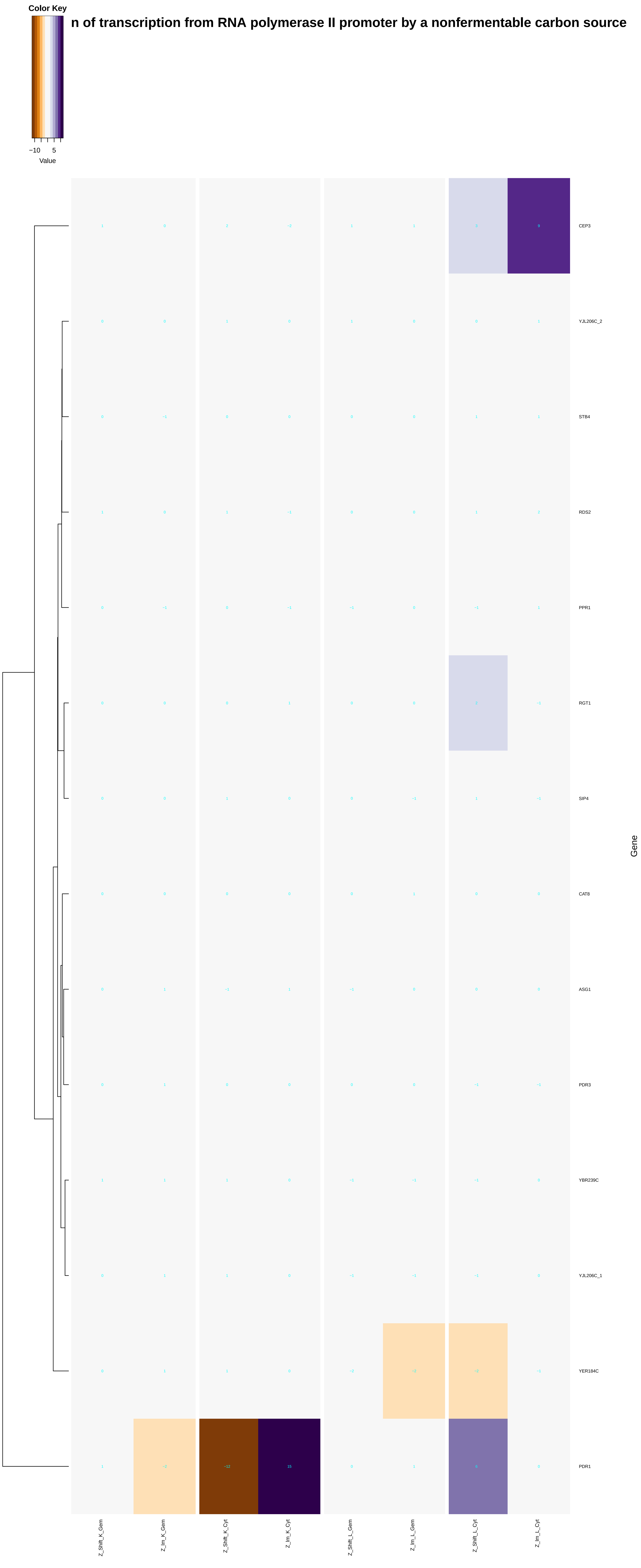
Color Key
−10 5
Value
n of transcription from RNA polymerase II promoter by a nonfermentable carbon source
CEP3
YJL206C_2
STB4
RDS2
PPR1
RGT1
SIP4
CAT8
ASG1
PDR3
YBR239C
YJL206C_1
YER184C
PDR1
Gene
Z_Shift_K_Gem
Z_Im_K_Gem
Z_Shift_K_Cyt
Z_Im_K_Cyt
Z_Shift_L_Gem
Z_Im_L_Gem
Z_Shift_L_Cyt
Z_Im_L_Cyt

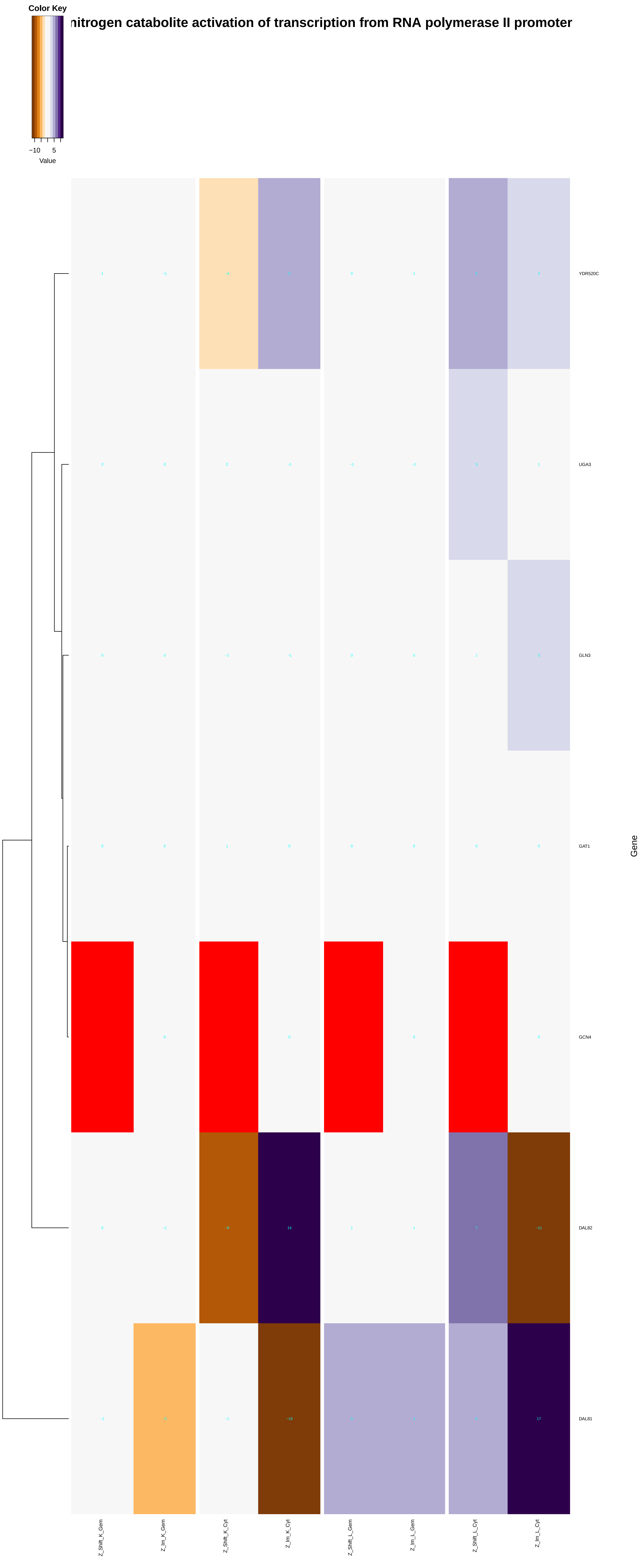
nitrogen catabolite activation of transcription from RNA polymerase II promoter
Color Key
−10
5
Value
YDR520C
UGA3
GLN3
GAT1
GCN4
DAL82
DAL81
Gene
Z_Shift_K_Gem
Z_Im_K_Gem
Z_Shift_K_Cyt
Z_Im_K_Cyt
Z_Shift_L_Gem
Z_Im_L_Gem
Z_Shift_L_Cyt
Z_Im_L_Cyt

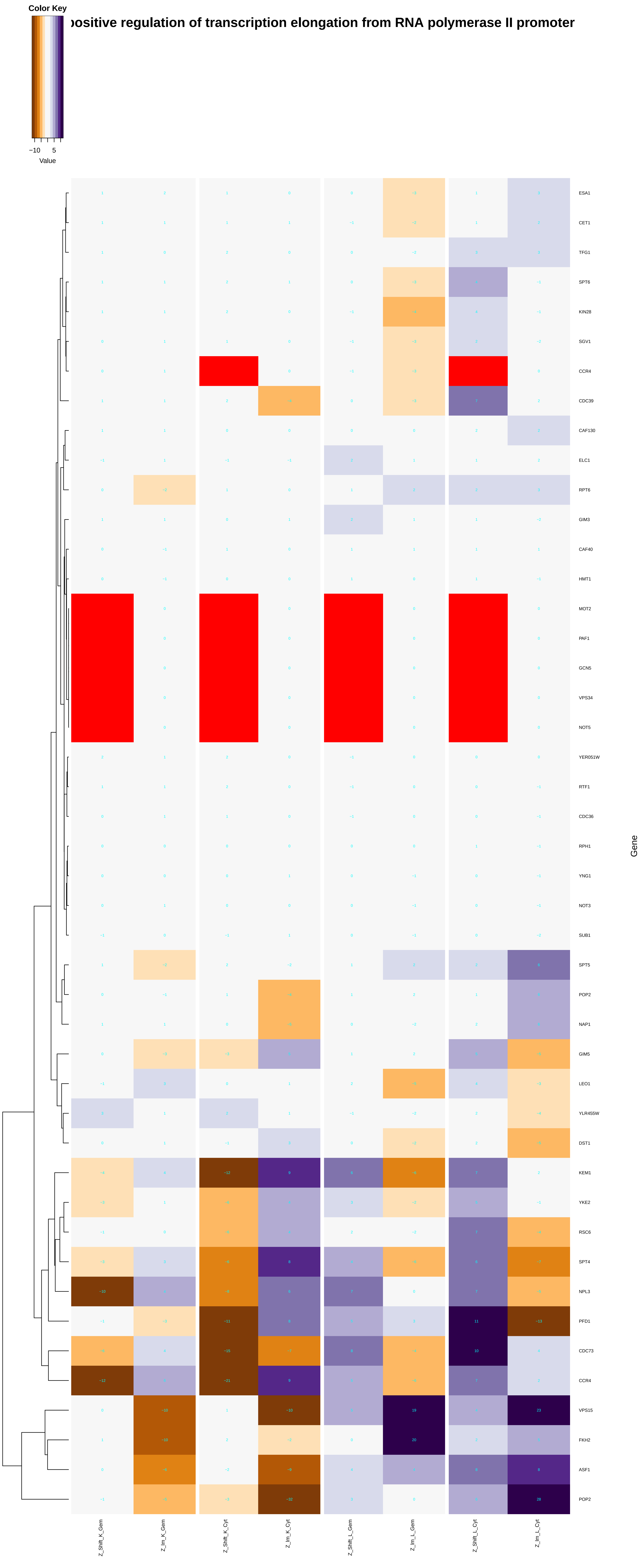

Color Key
−10
5
Value
positive regulation of transcription elongation from RNA polymerase II promoter
ESA1
CET1
TFG1
SPT6
KIN28
SGV1
CCR4
CDC39
CAF130
ELC1
RPT6
GIM3
CAF40
HMT1
MOT2
PAF1
GCN5
VPS34
NOT5
YER051W
RTF1
CDC36
RPH1
YNG1
NOT3
SUB1
SPT5
POP2
NAP1
GIM5
LEO1
YLR455W
DST1
KEM1
YKE2
RSC6
SPT4
NPL3
PFD1
CDC73
CCR4
VPS15
FKH2
ASF1
POP2
Gene
Z_Shift_K_Gem
Z_Im_K_Gem
Z_Shift_K_Cyt
Z_Im_K_Cyt
Z_Shift_L_Gem
Z_Im_L_Gem
Z_Shift_L_Cyt
Z_Im_L_Cyt

n of dipeptide transport by regulation of transcription from RNA polymerase II promoter

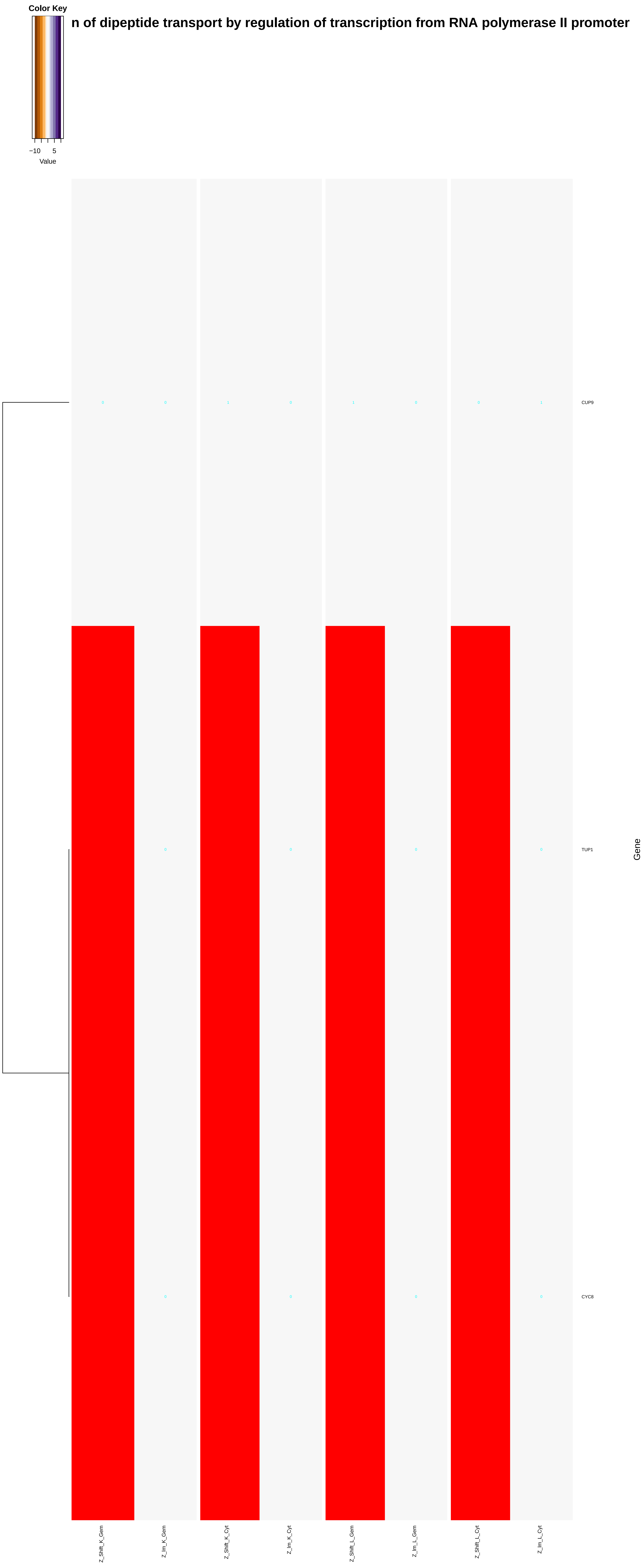

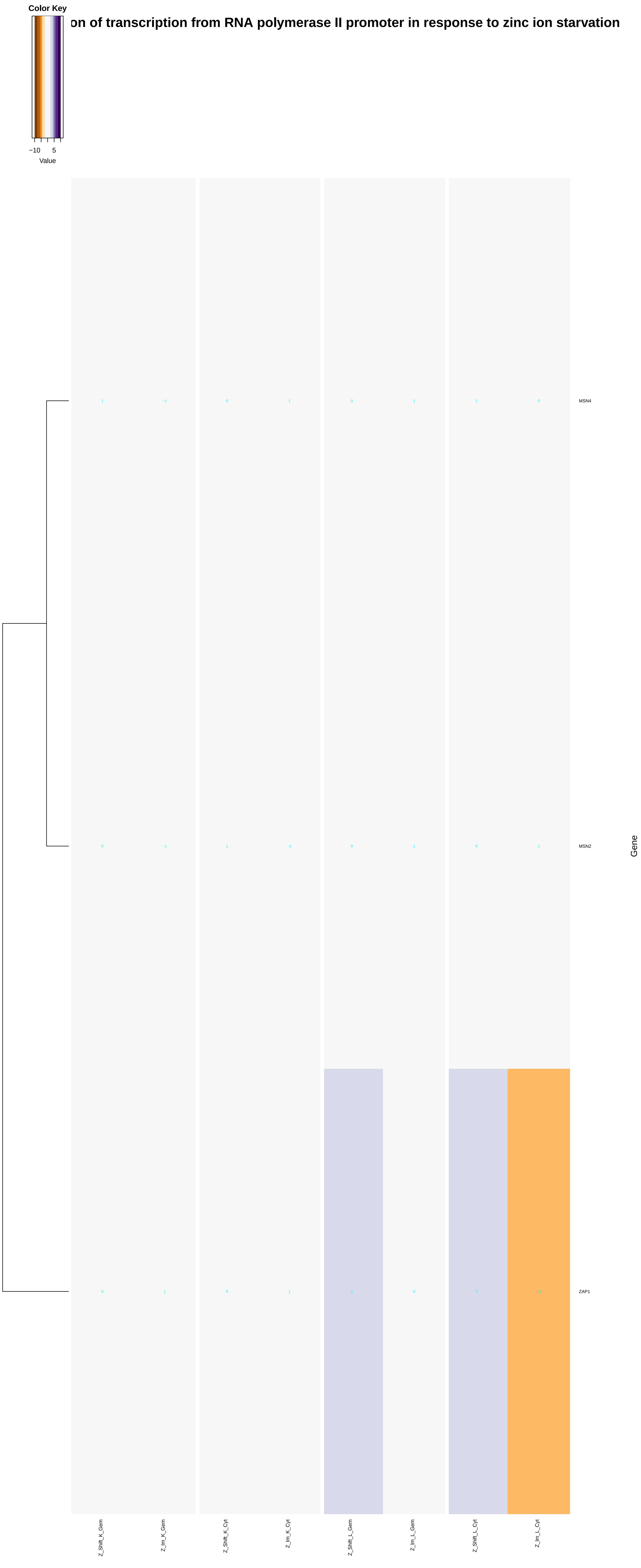

on of transcription from RNA polymerase II promoter in response to zinc ion starvation
Color Key
−10
5
Value
MSN4
MSN2
ZAP1
Gene
Z_Shift_K_Gem
Z_lm_K_Gem
Z_Shift_K_Cyt
Z_lm_K_Cyt
Z_Shift_L_Gem
Z_lm_L_Gem
Z_Shift_L_Cyt
Z_lm_L_Cyt

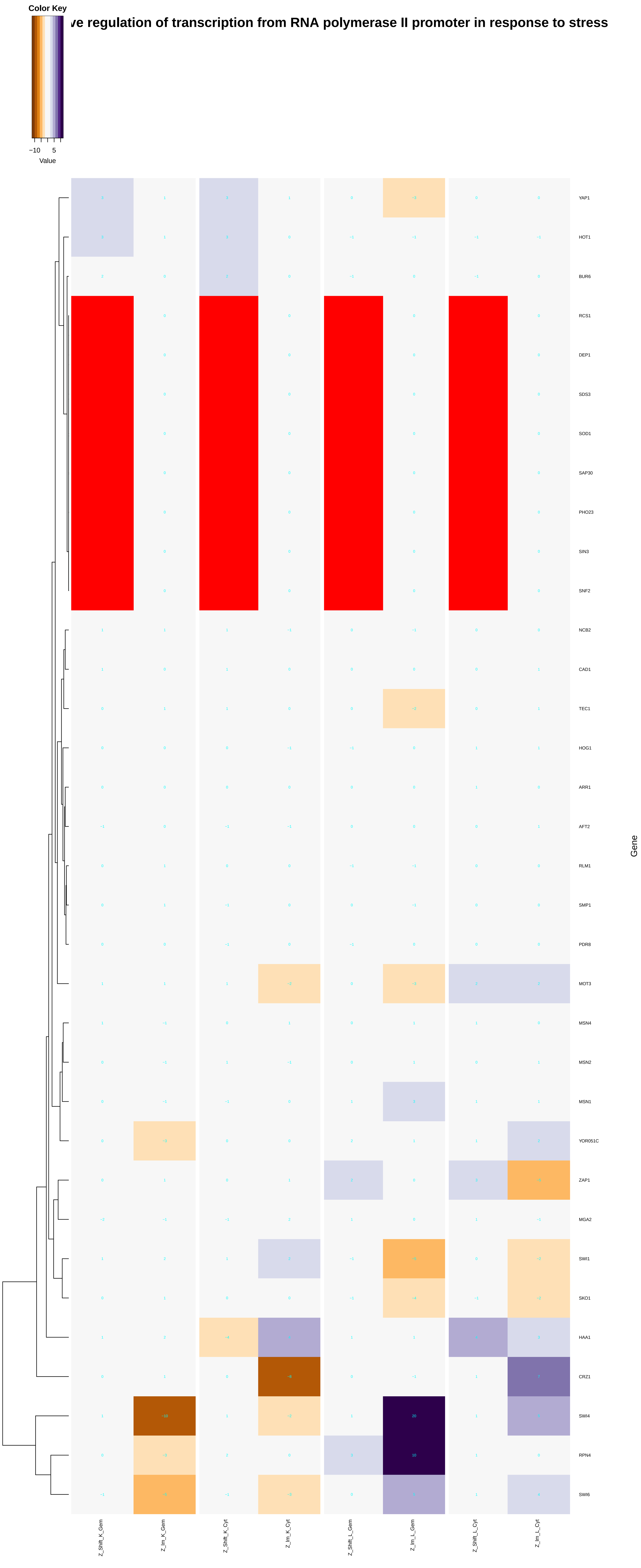
ve regulation of transcription from RNA polymerase II promoter in response to stress
Color Key
−10 5
Value
YAP1
HOT1
BUR6
RCS1
DEP1
SDS3
SOD1
SAP30
PHO23
SIN3
SNF2
NCB2
CAD1
TEC1
HOG1
ARR1
AFT2
RLM1
SMP1
PDR8
MOT3
MSN4
MSN2
MSN1
YOR051C
ZAP1
MGA2
SWI1
SKO1
HAA1
CRZ1
SWI4
RPN4
SWI6
Gene
Z_Shift_K_Gem
Z_lm_K_Gem
Z_Shift_K_Cyt
Z_lm_K_Cyt
Z_Shift_L_Gem
Z_lm_L_Gem
Z_Shift_L_Cyt
Z_lm_L_Cyt

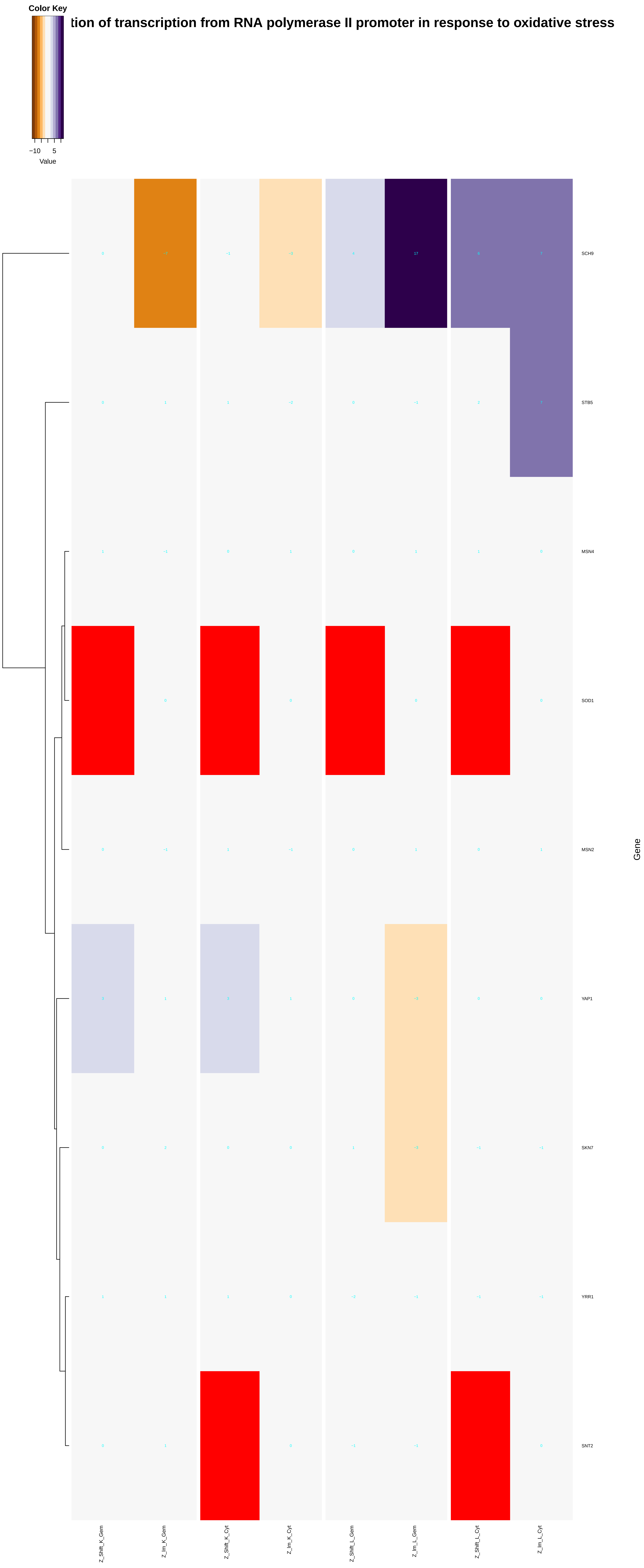

Color Key
−10
5
Value
tion of transcription from RNA polymerase II promoter in response to oxidative stress
SCH9
STB5
MSN4
SOD1
MSN2
YAP1
SKN7
YRR1
SNT2
Gene
Z_Shift_K_Gem
Z_Im_K_Gem
Z_Shift_K_Cyt
Z_Im_K_Cyt
Z_Shift_L_Gem
Z_Im_L_Gem
Z_Shift_L_Cyt
Z_Im_L_Cyt

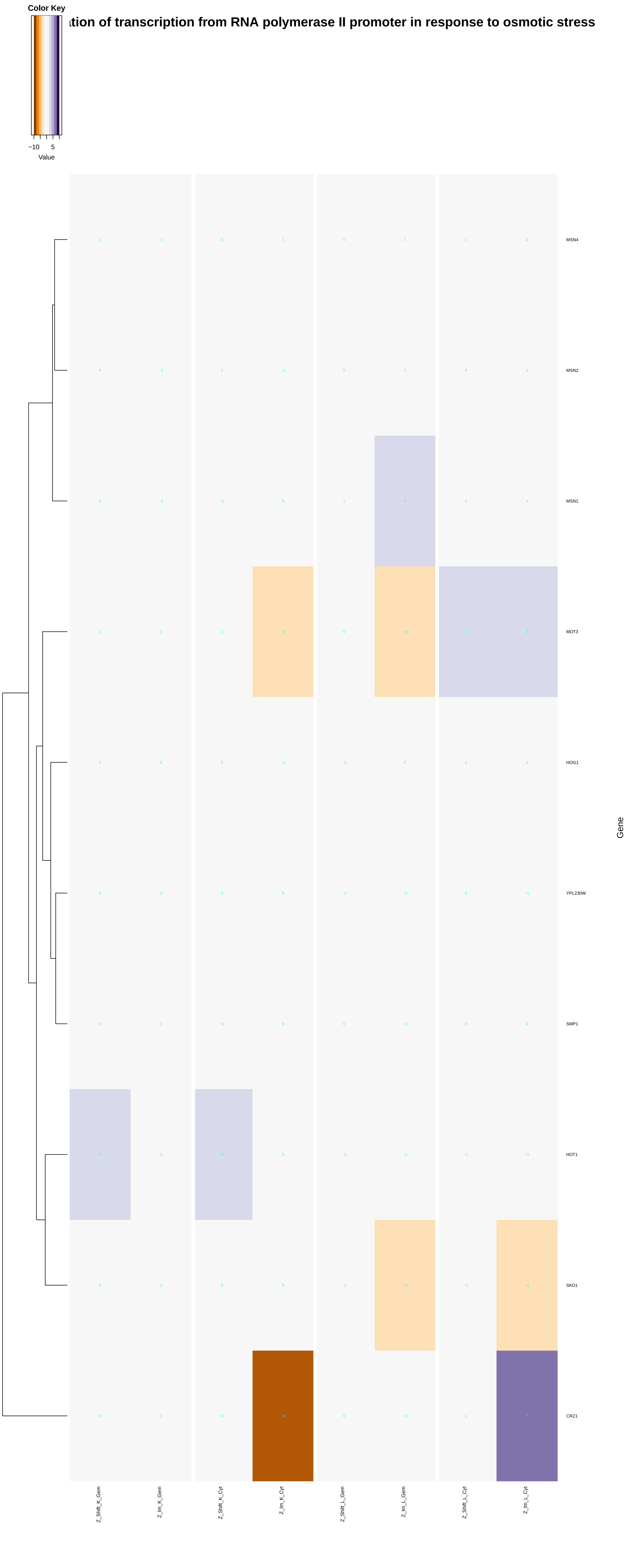
Color Key
−10
5
Value
ıtion of transcription from RNA polymerase II promoter in response to osmotic stress
MSN4
MSN2
MSN1
MOT3
HOG1
YPL230W
SMP1
HOT1
SKO1
CRZ1
Gene
Z_Shift_K_Gem
Z_Im_K_Gem
Z_Shift_K_Cyt
Z_Im_K_Cyt
Z_Shift_L_Gem
Z_Im_L_Gem
Z_Shift_L_Cyt
Z_Im_L_Cyt

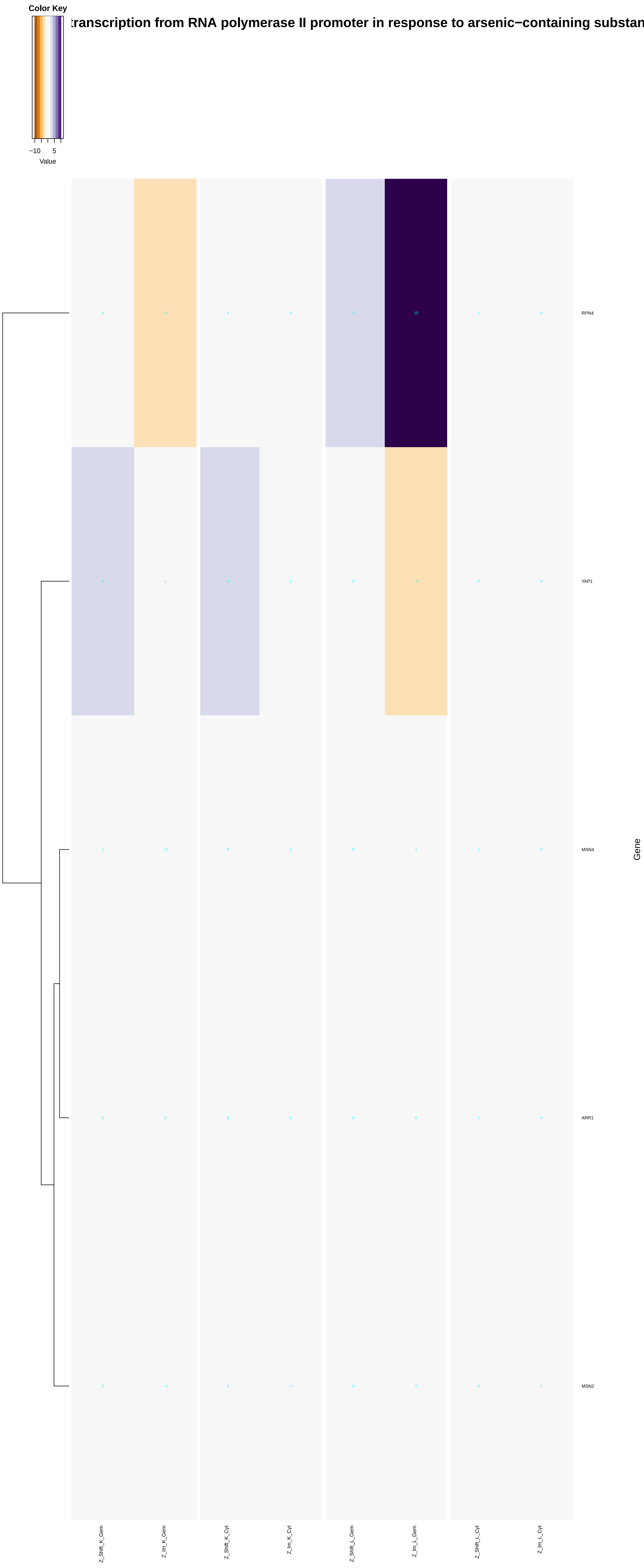
transcription from RNA polymerase II promoter in response to arsenic−containing substan
Color Key
−10
5
Value
RPN4
YAP1
MSN4
ARR1
MSN2
Gene
Z_Shift_K_Gem
Z_Im_K_Gem
Z_Shift_K_Cyt
Z_Im_K_Cyt
Z_Shift_L_Gem
Z_Im_L_Gem
Z_Shift_L_Cyt
Z_Im_L_Cyt

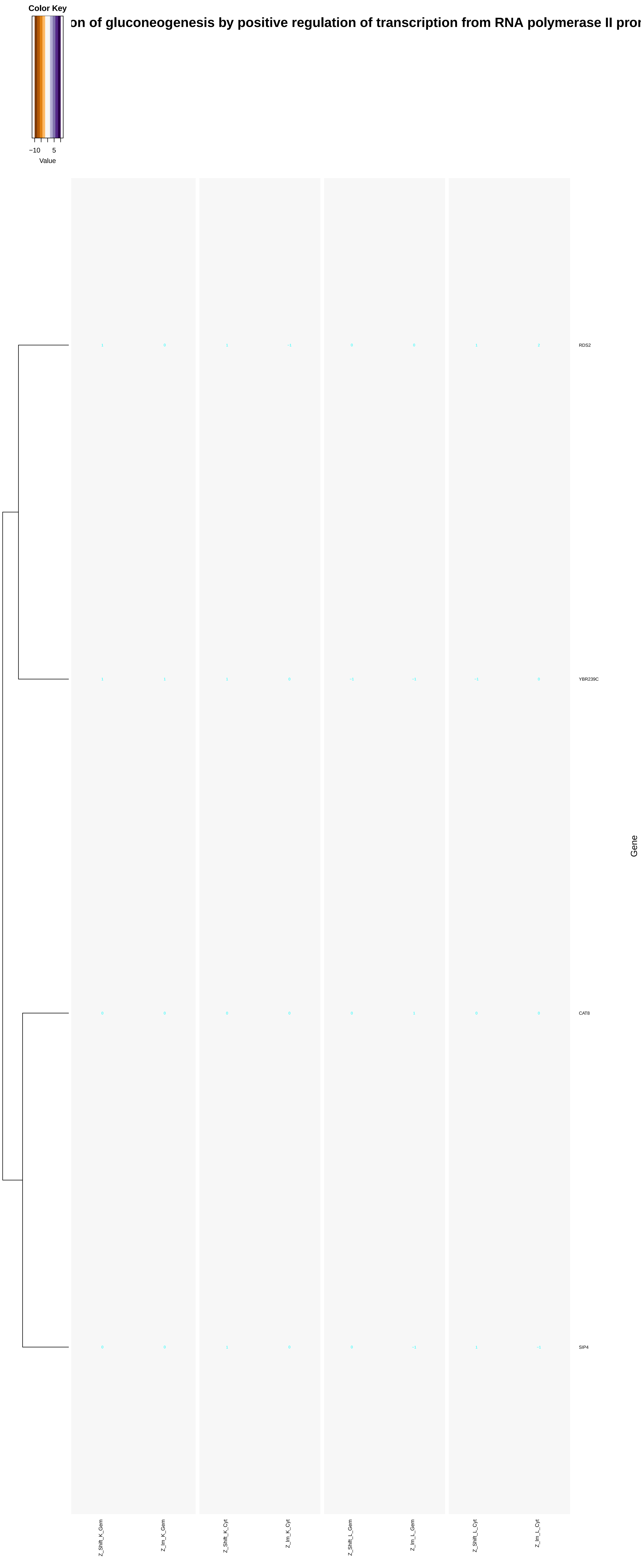

of gluconeogenesis by positive regulation of transcription from RNA polymerase II
Color Key
−10
5
Value
RDS2
YBR239C
CAT8
SIP4
Gene
Z_Shift_K_Gem
Z_Im_K_Gem
Z_Shift_K_Cyt
Z_Im_K_Cyt
Z_Shift_L_Gem
Z_Im_L_Gem
Z_Shift_L_Cyt
Z_Im_L_Cyt

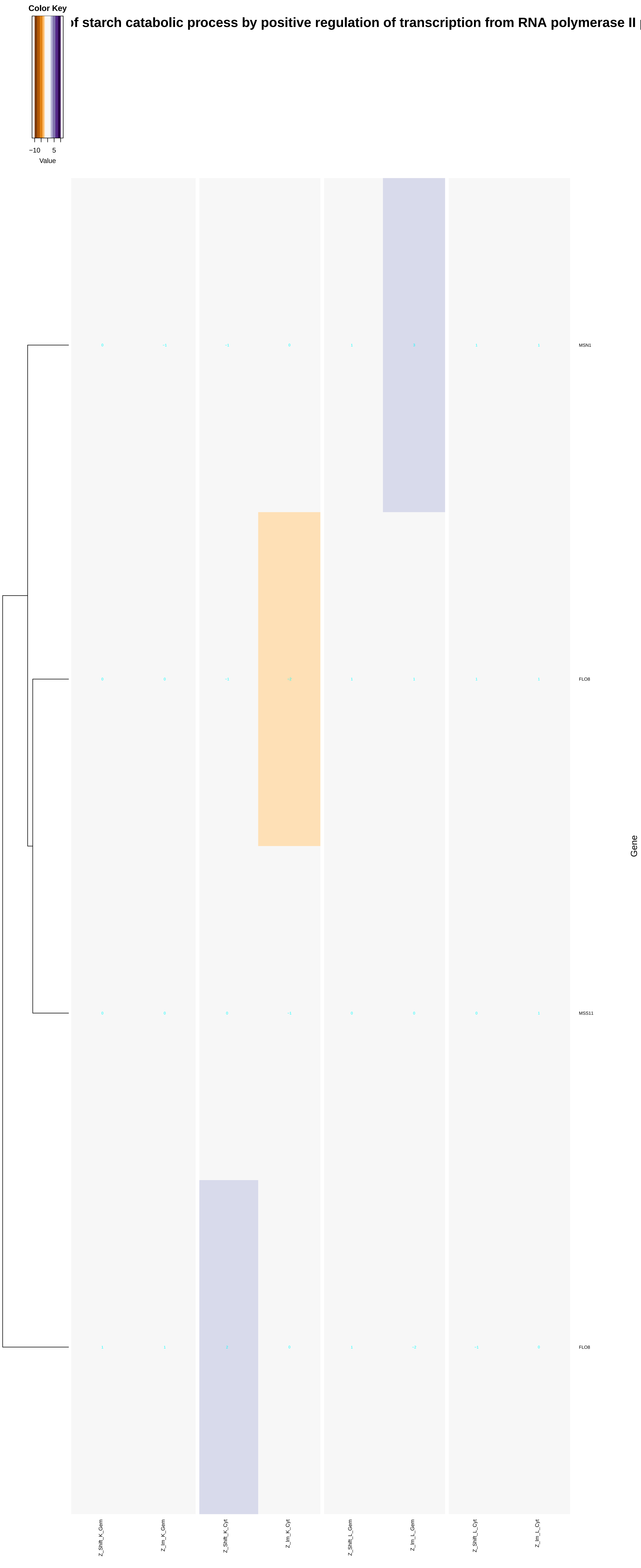
of starch catabolic process by positive regulation of transcription from RNA polymerase II p
Color Key
−10
5
Value
MSN1
FLO8
MSS11
FLO8
Gene
Z_Shift_K_Gem
Z_Im_K_Gem
Z_Shift_K_Cyt
Z_Im_K_Cyt
Z_Shift_L_Gem
Z_Im_L_Gem
Z_Shift_L_Cyt
Z_Im_L_Cyt

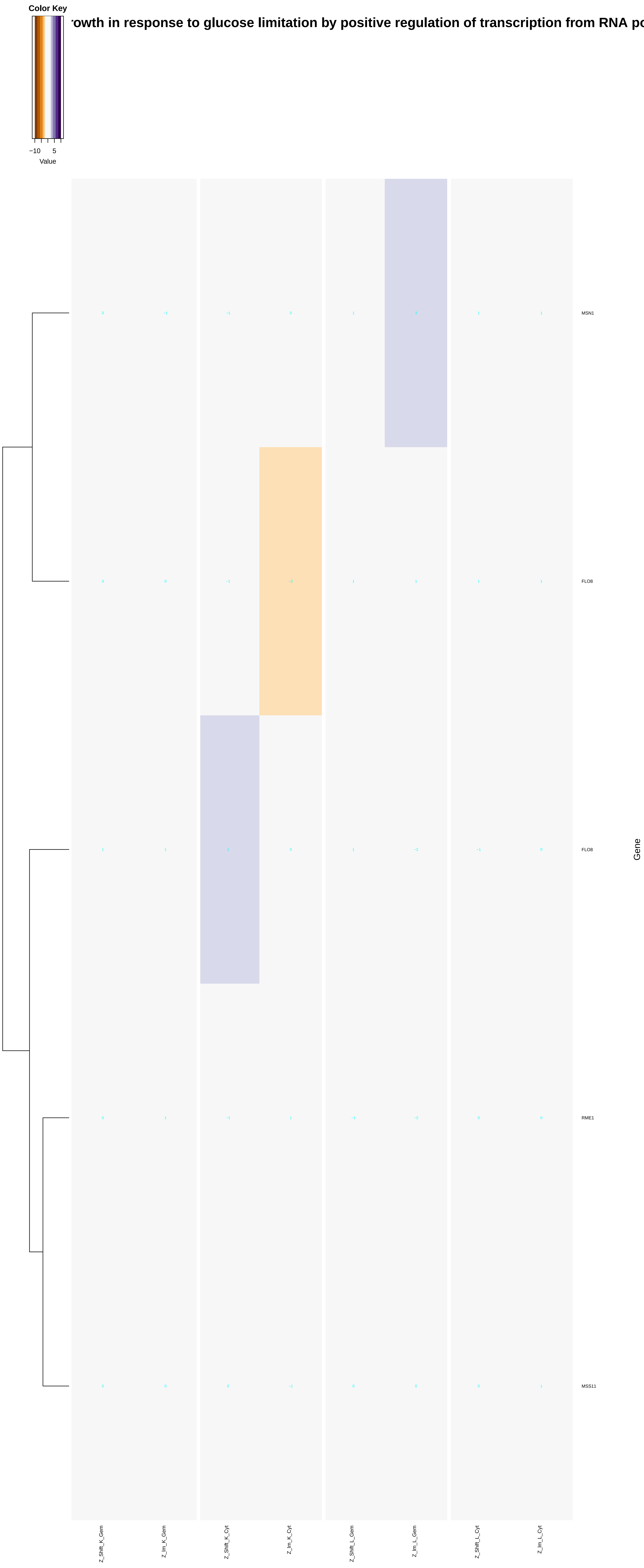
owth in response to glucose limitation by positive regulation of transcription from RNA po
Color Key
−10
5
Value
MSN1
FLO8
FLO8
RME1
MSS11
Gene
Z_Shift_K_Gem
Z_Im_K_Gem
Z_Shift_K_Cyt
Z_Im_K_Cyt
Z_Shift_L_Gem
Z_Im_L_Gem
Z_Shift_L_Cyt
Z_Im_L_Cyt

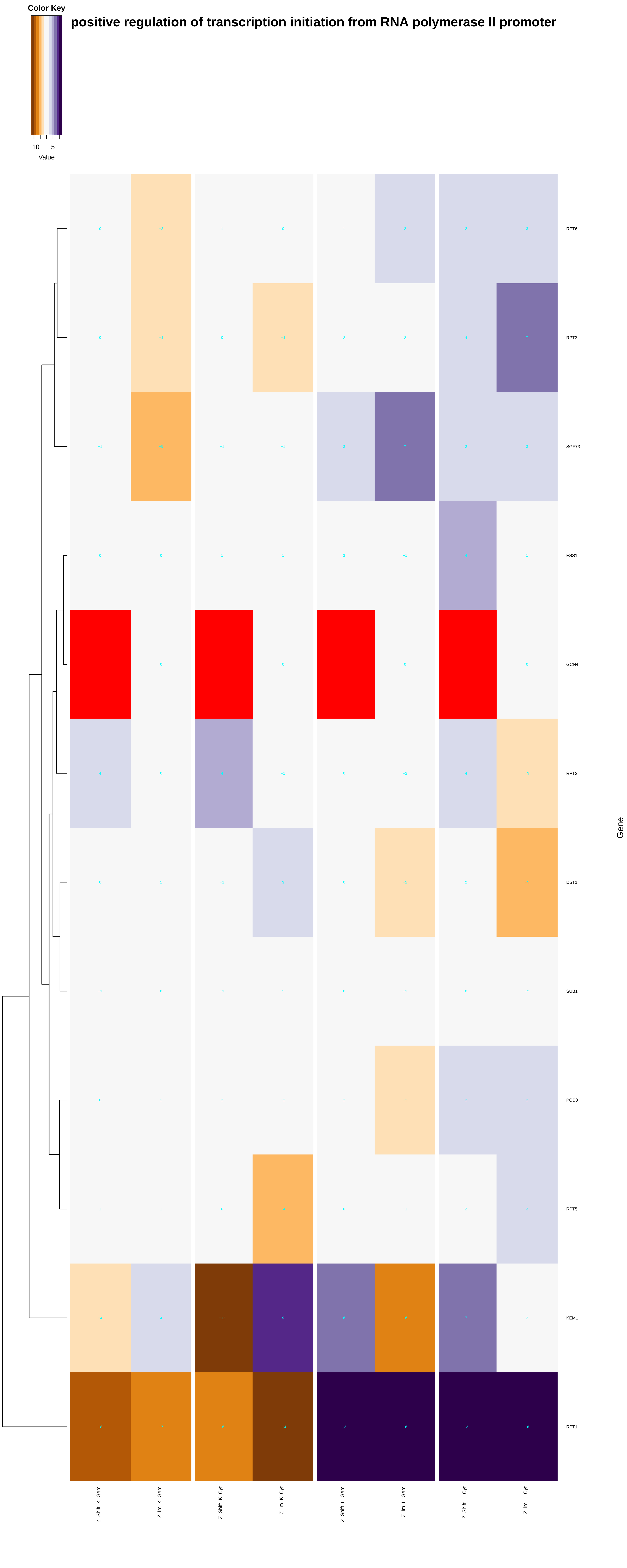
Color Key
−10
5
Value
positive regulation of transcription initiation from RNA polymerase II promoter
RPT6
RPT3
SGF73
ESS1
GCN4
RPT2
DST1
SUB1
POB3
RPT5
KEM1
RPT1
Gene
Z_Shift_K_Gem
Z_Im_K_Gem
Z_Shift_K_Cyt
Z_Im_K_Cyt
Z_Shift_L_Gem
Z_Im_L_Gem
Z_Shift_L_Cyt
Z_Im_L_Cyt

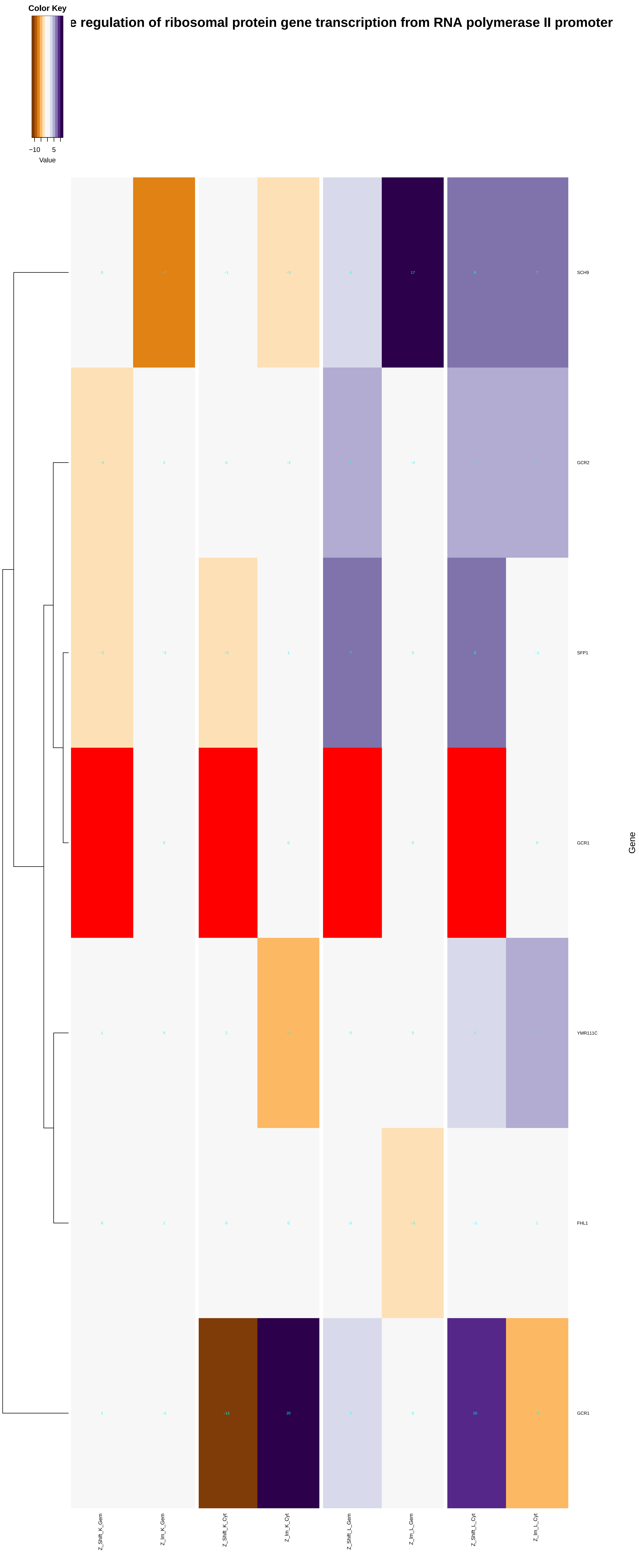
e regulation of ribosomal protein gene transcription from RNA polymerase II promoter
Color Key
−10
5
Value
SCH9
GCR2
SFP1
GCR1
YMR111C
FHL1
GCR1
Gene
Z_Shift_K_Gem
Z_Im_K_Gem
Z_Shift_K_Cyt
Z_Im_K_Cyt
Z_Shift_L_Gem
Z_Im_L_Gem
Z_Shift_L_Cyt
Z_Im_L_Cyt

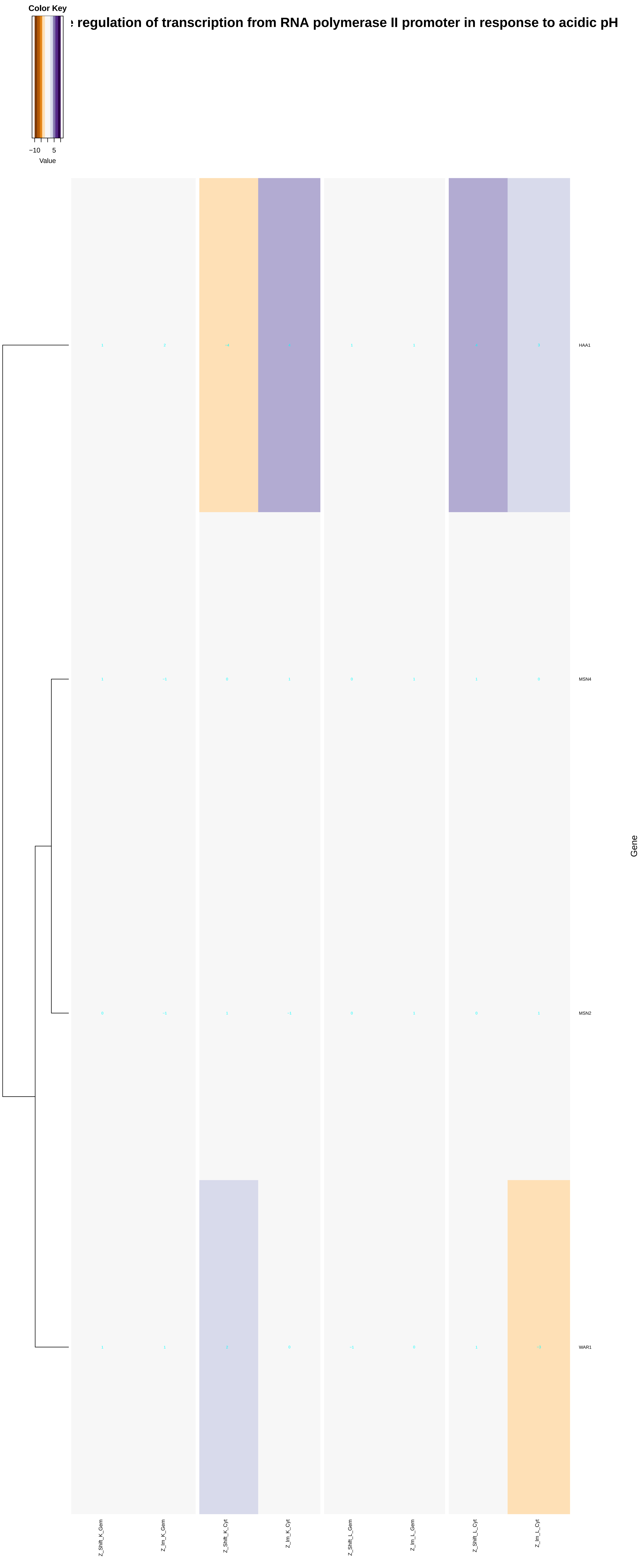

Color Key
−10
5
Value
e regulation of transcription from RNA polymerase II promoter in response to acidic pH
HAA1
MSN4
MSN2
WAR1
Gene
Z_Shift_K_Gem
Z_Im_K_Gem
Z_Shift_K_Cyt
Z_Im_K_Cyt
Z_Shift_L_Gem
Z_Im_L_Gem
Z_Shift_L_Cyt
Z_Im_L_Cyt

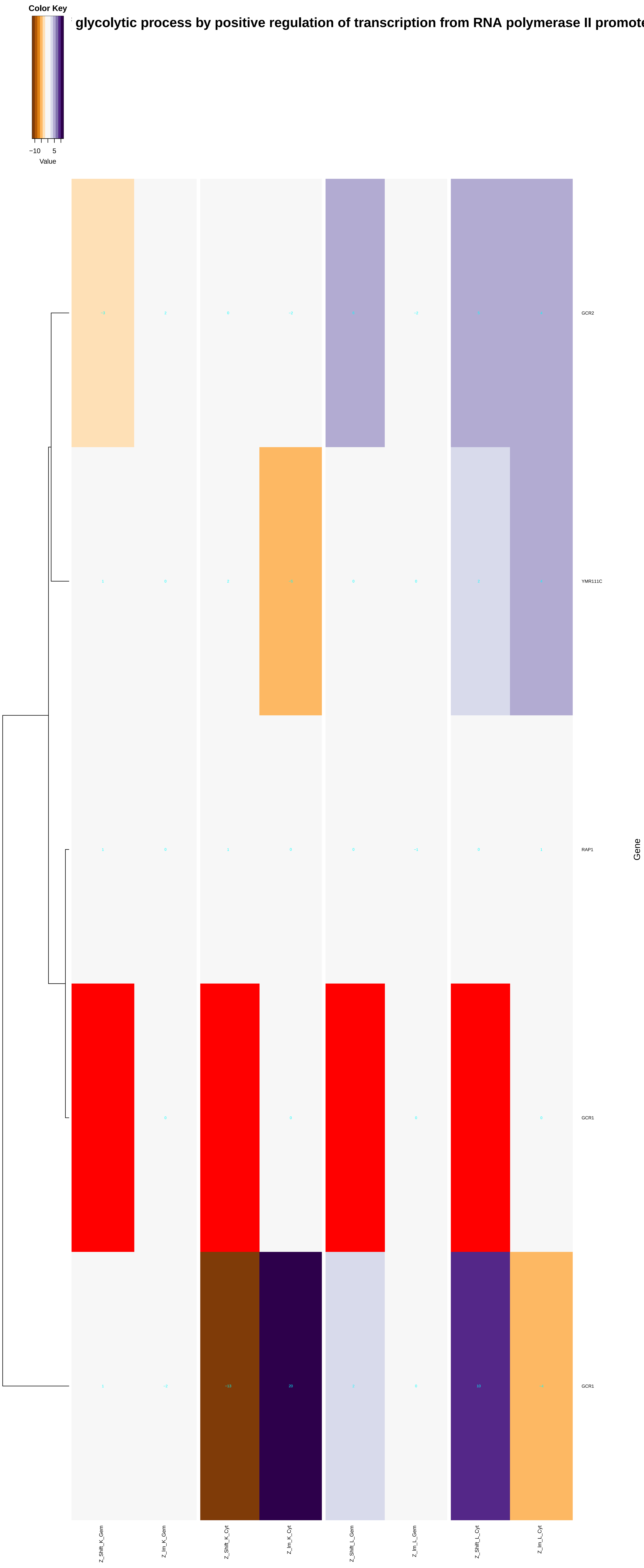
Color Key
−10
5
Value
glycolytic process by positive regulation of transcription from RNA polymerase II promote
GCR2
YMR111C
RAP1
GCR1
GCR1
Gene
Z_Shift_K_Gem
Z_Im_K_Gem
Z_Shift_K_Cyt
Z_Im_K_Cyt
Z_Shift_L_Gem
Z_Im_L_Gem
Z_Shift_L_Cyt
Z_Im_L_Cyt

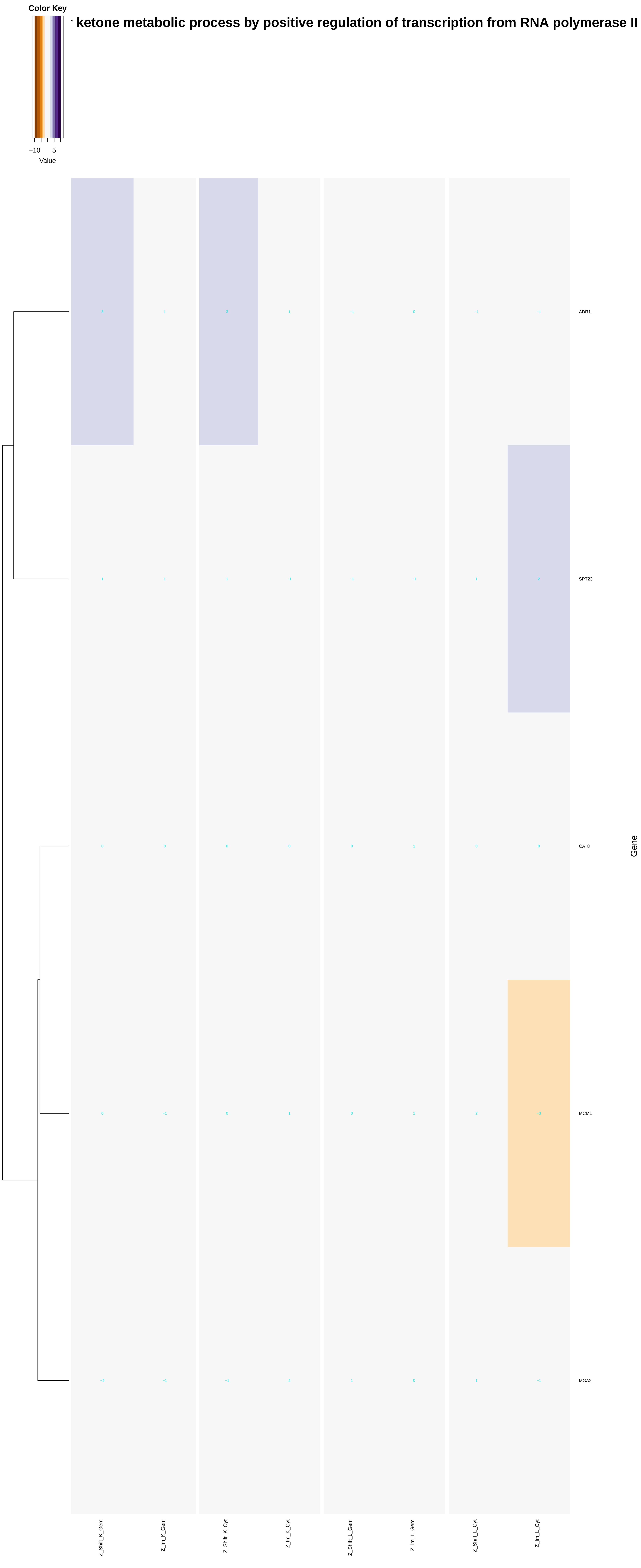

Color Key
−10
5
Value
ketone metabolic process by positive regulation of transcription from RNA polymerase II
ADR1
SPT23
CAT8
MCM1
MGA2
Gene
Z_Shift_K_Gem
Z_Im_K_Gem
Z_Shift_K_Cyt
Z_Im_K_Cyt
Z_Shift_L_Gem
Z_Im_L_Gem
Z_Shift_L_Cyt
Z_Im_L_Cyt

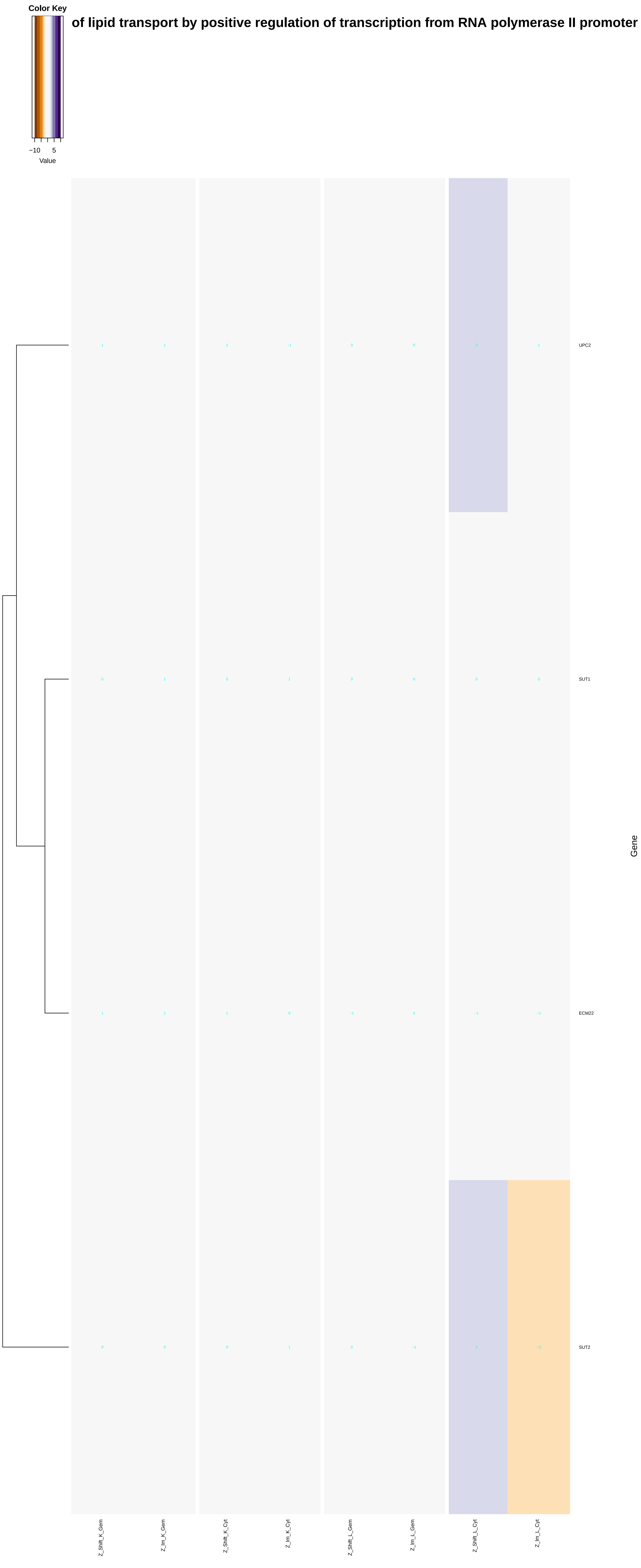
of lipid transport by positive regulation of transcription from RNA polymerase II promoter
Color Key
−10
5
Value
UPC2
SUT1
ECM22
SUT2
Gene
Z_Shift_K_Gem
Z_Im_K_Gem
Z_Shift_K_Cyt
Z_Im_K_Cyt
Z_Shift_L_Gem
Z_Im_L_Gem
Z_Shift_L_Cyt
Z_Im_L_Cyt

ɔsitive regulation of transcription involved in G2/M transition of mitotic cell cycle

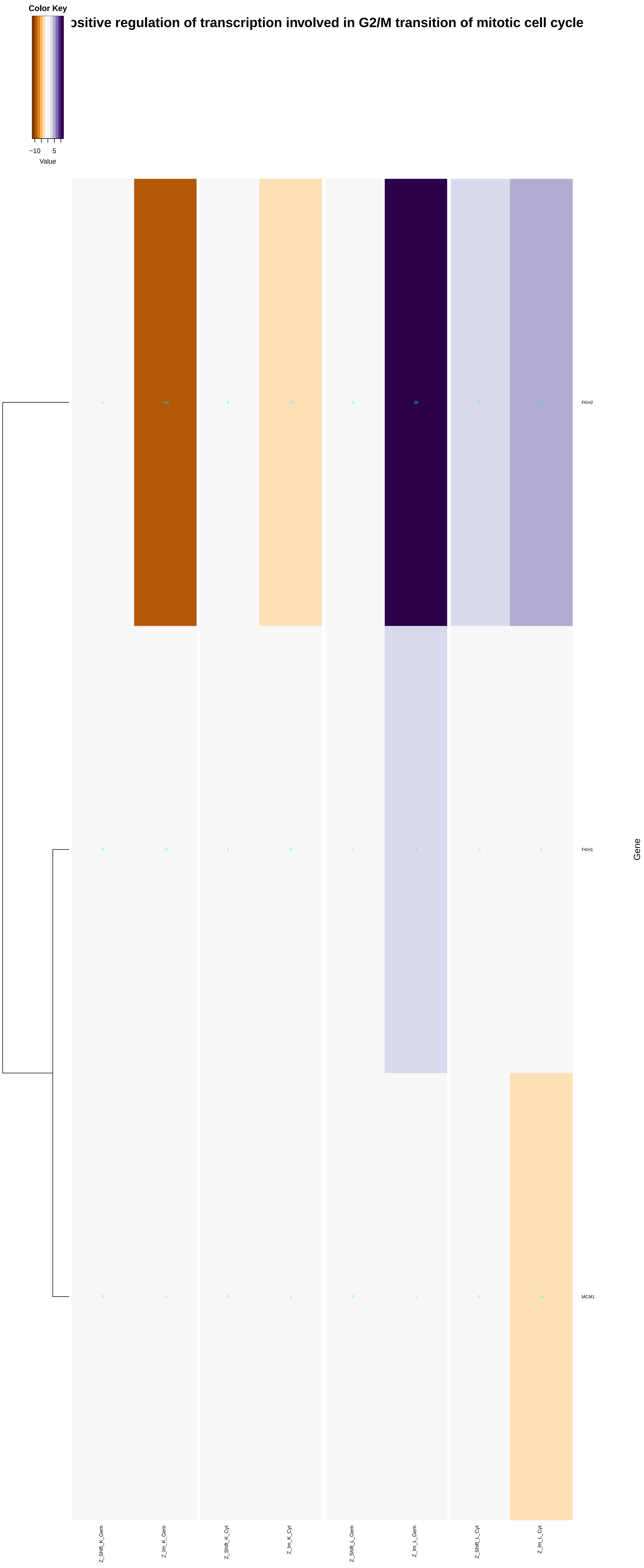

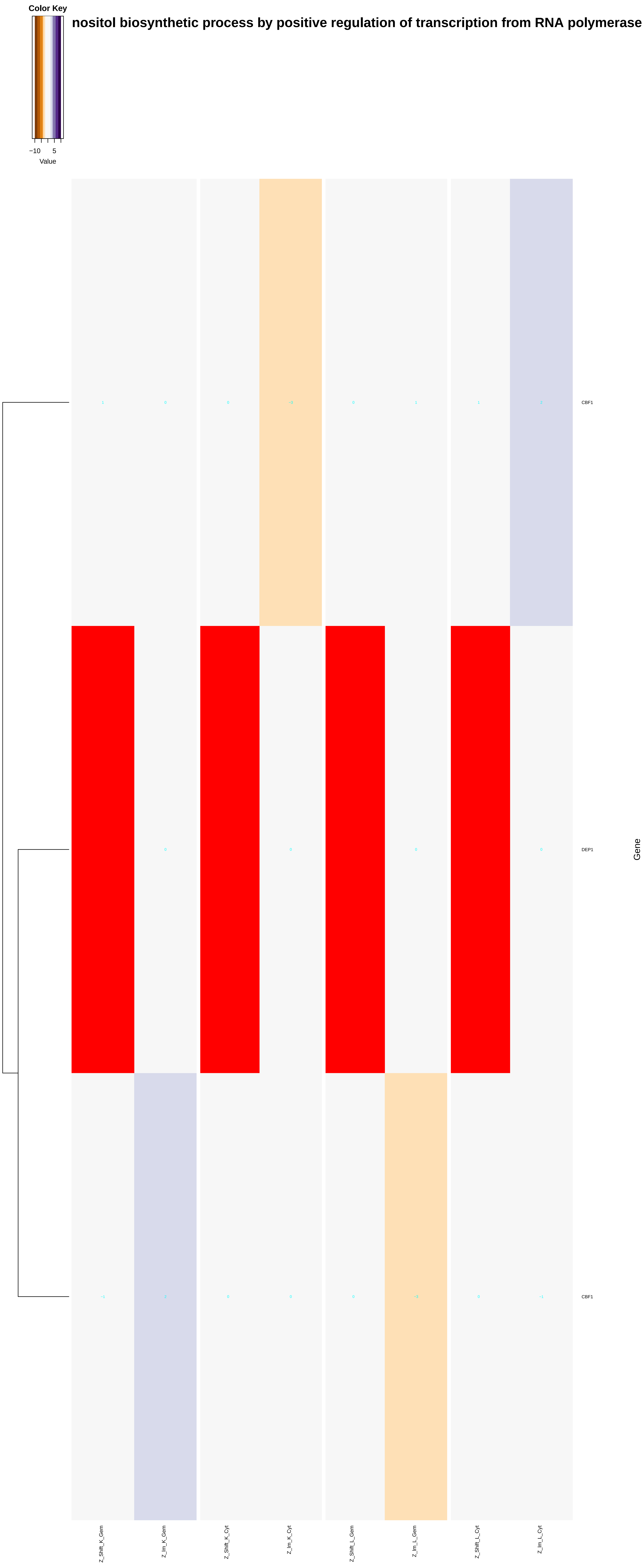
Color Key
−10
5
Value
nositol biosynthetic process by positive regulation of transcription from RNA polymerase
CBF1
DEP1
CBF1
Gene
Z_Shift_K_Gem
Z_Im_K_Gem
Z_Shift_K_Cyt
Z_Im_K_Cyt
Z_Shift_L_Gem
Z_Im_L_Gem
Z_Shift_L_Cyt
Z_Im_L_Cyt

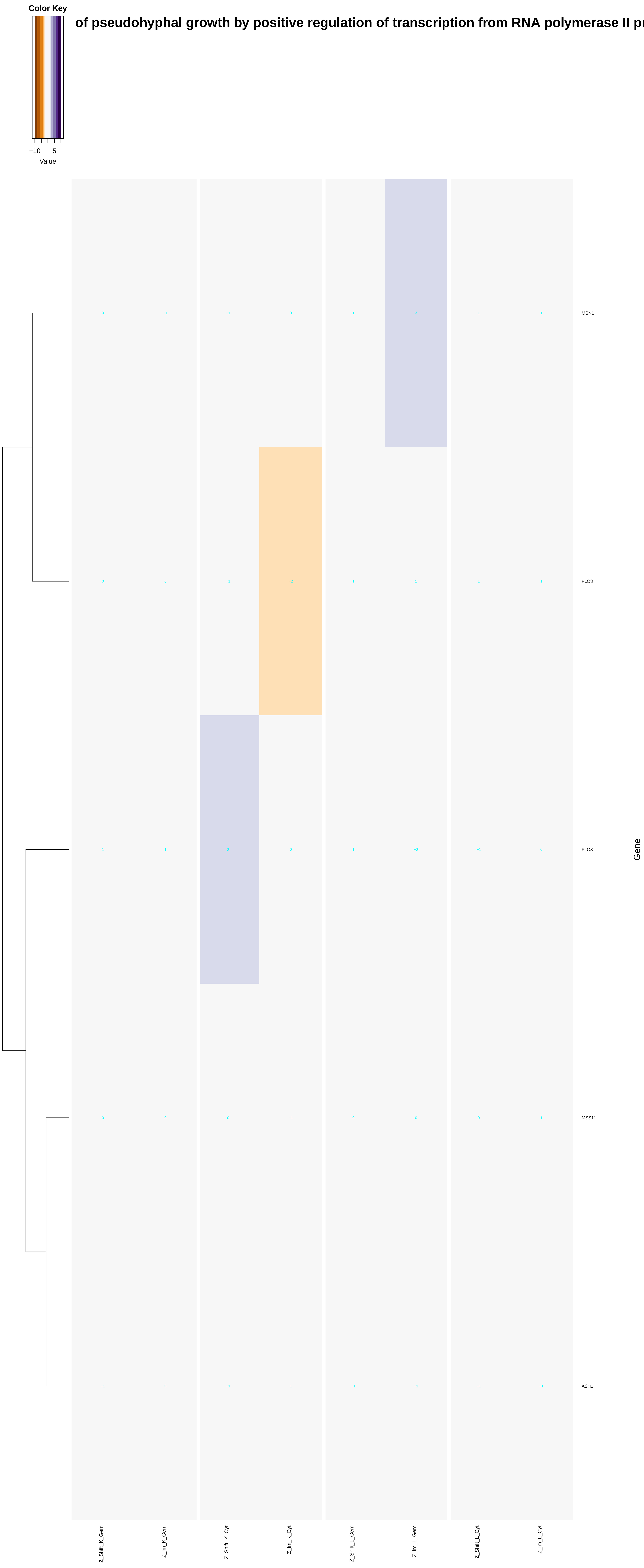

Color Key
−10 5
Value
of pseudohyphal growth by positive regulation of transcription from RNA polymerase II pr
MSN1
FLO8
FLO8
MSS11
ASH1
Gene
Z_Shift_K_Gem
Z_Im_K_Gem
Z_Shift_K_Cyt
Z_Im_K_Cyt
Z_Shift_L_Gem
Z_Im_L_Gem
Z_Shift_L_Cyt
Z_Im_L_Cyt

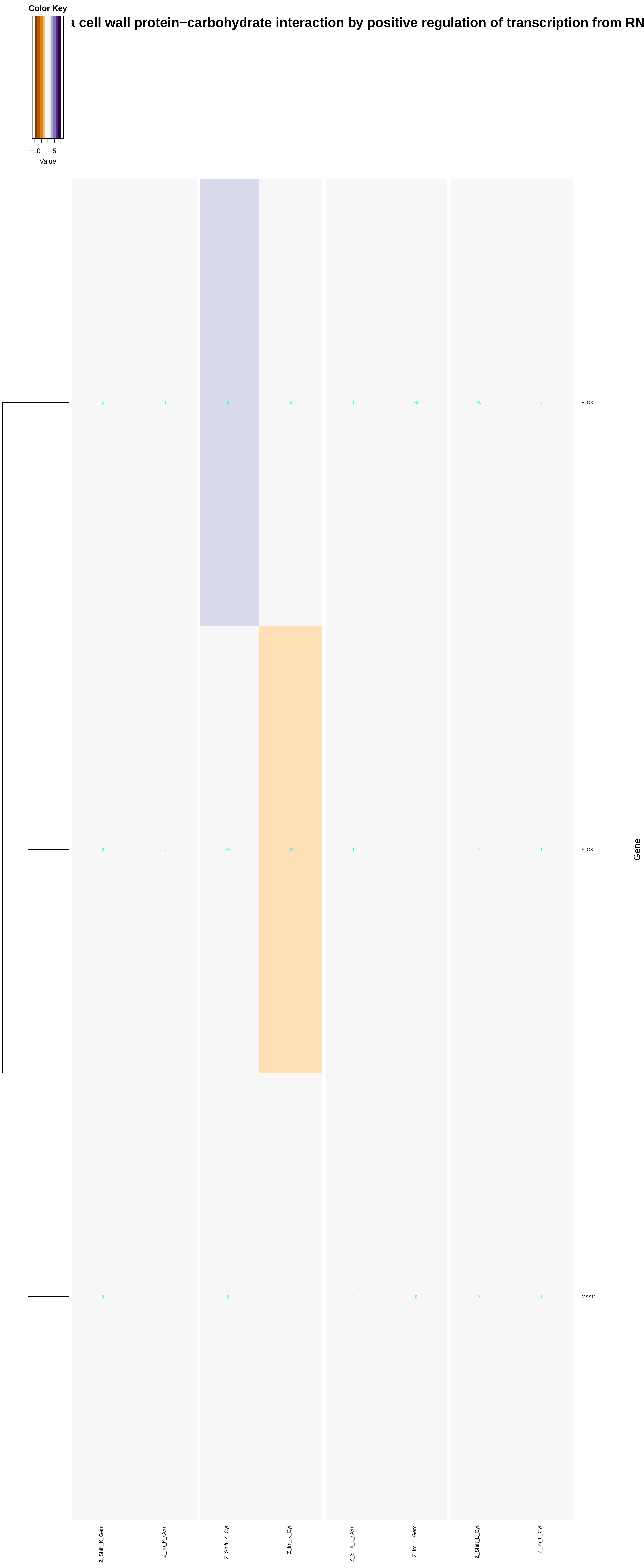
a cell wall protein−carbohydrate interaction by positive regulation of transcription from RN
Color Key
−10
5
Value
FLO8
FLO8
MSS11
Gene
Z_Shift_K_Gem
Z_Im_K_Gem
Z_Shift_K_Cyt
Z_Im_K_Cyt
Z_Shift_L_Gem
Z_Im_L_Gem
Z_Shift_L_Cyt
Z_Im_L_Cyt

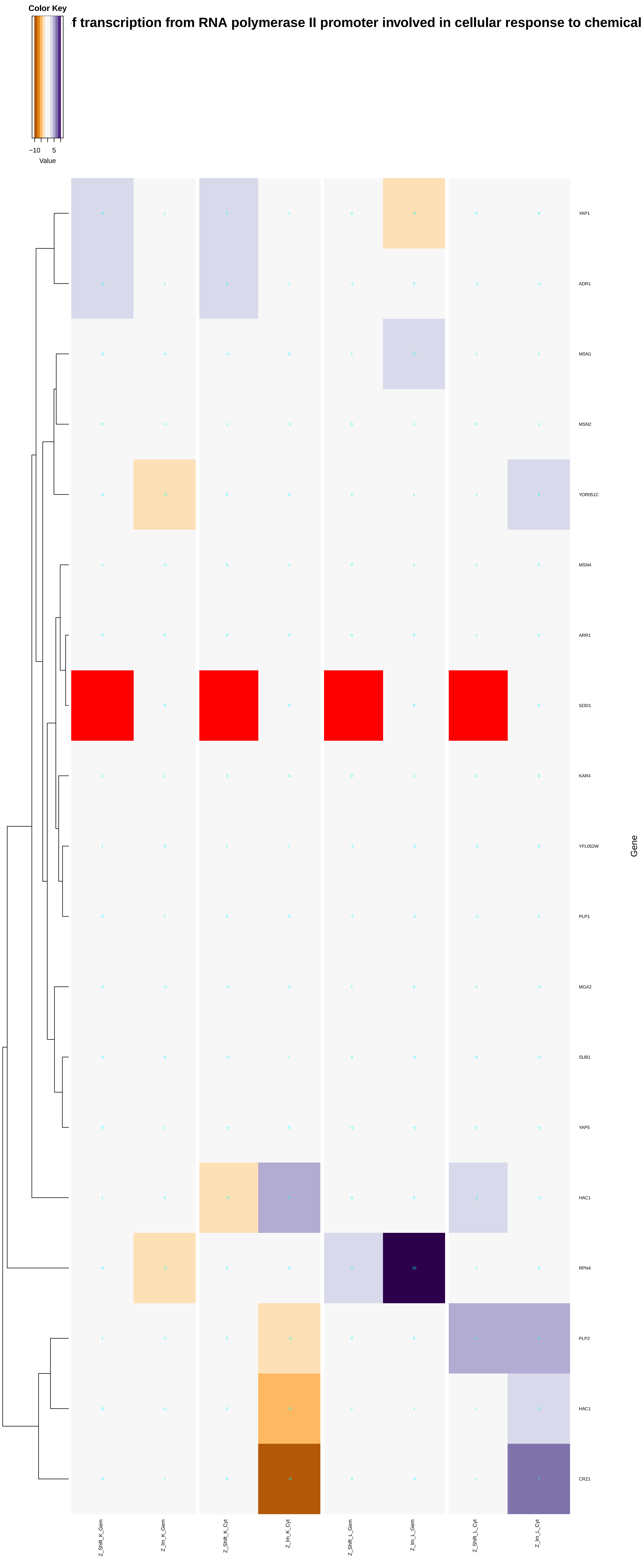
Color Key
−10
5
Value
f transcription from RNA polymerase II promoter involved in cellular response to chemical
YAP1
ADR1
MSN1
MSN2
YOR051C
MSN4
ARR1
SOD1
KAR4
YFL052W
PLP1
MGA2
SUB1
YAP5
HAC1
RPN4
PLP2
HAC1
CRZ1
Gene
Z_Shift_K_Gem
Z_Im_K_Gem
Z_Shift_K_Cyt
Z_Im_K_Cyt
Z_Shift_L_Gem
Z_Im_L_Gem
Z_Shift_L_Cyt
Z_Im_L_Cyt

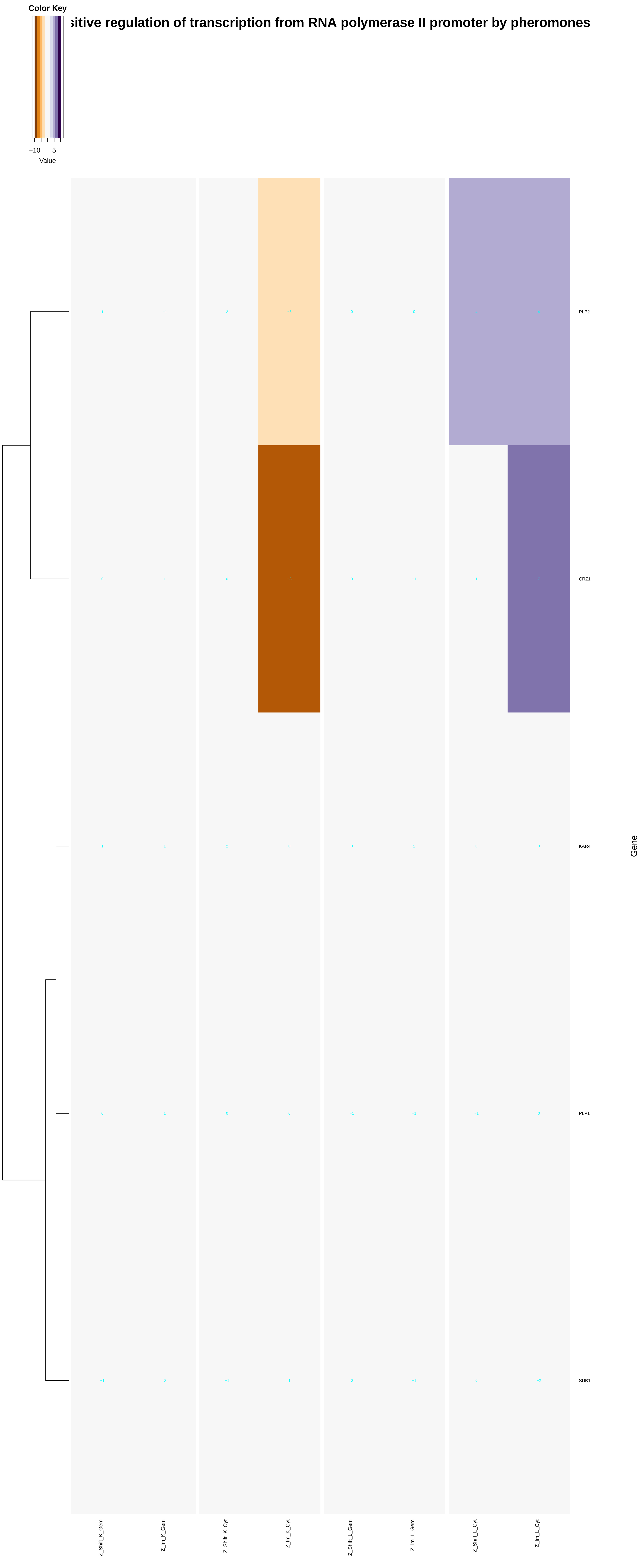

Color Key
−10
5
Value
sitive regulation of transcription from RNA polymerase II promoter by pheromones
PLP2
CRZ1
KAR4
PLP1
SUB1
Gene
Z_Shift_K_Gem
Z_Im_K_Gem
Z_Shift_K_Cyt
Z_Im_K_Cyt
Z_Shift_L_Gem
Z_Im_L_Gem
Z_Shift_L_Cyt
Z_Im_L_Cyt

regulation of transcriptional start site selection at RNA polymerase II promoter

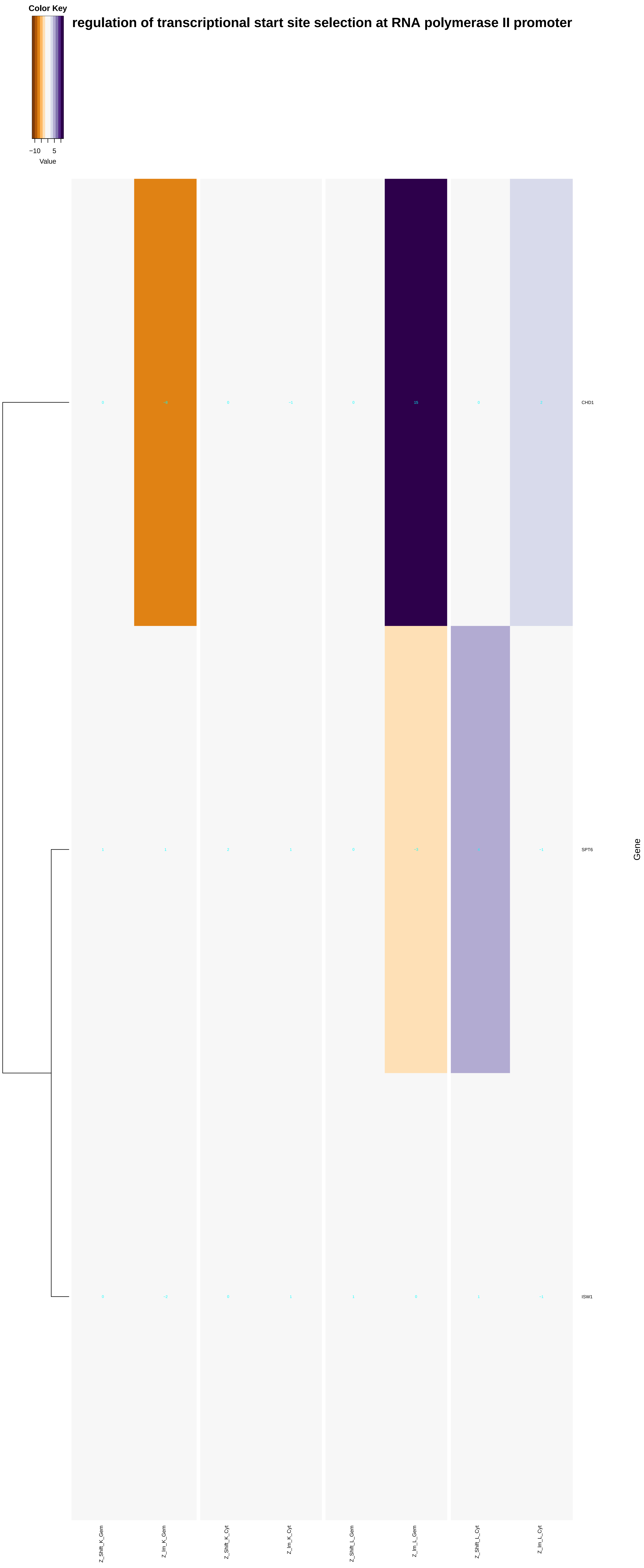

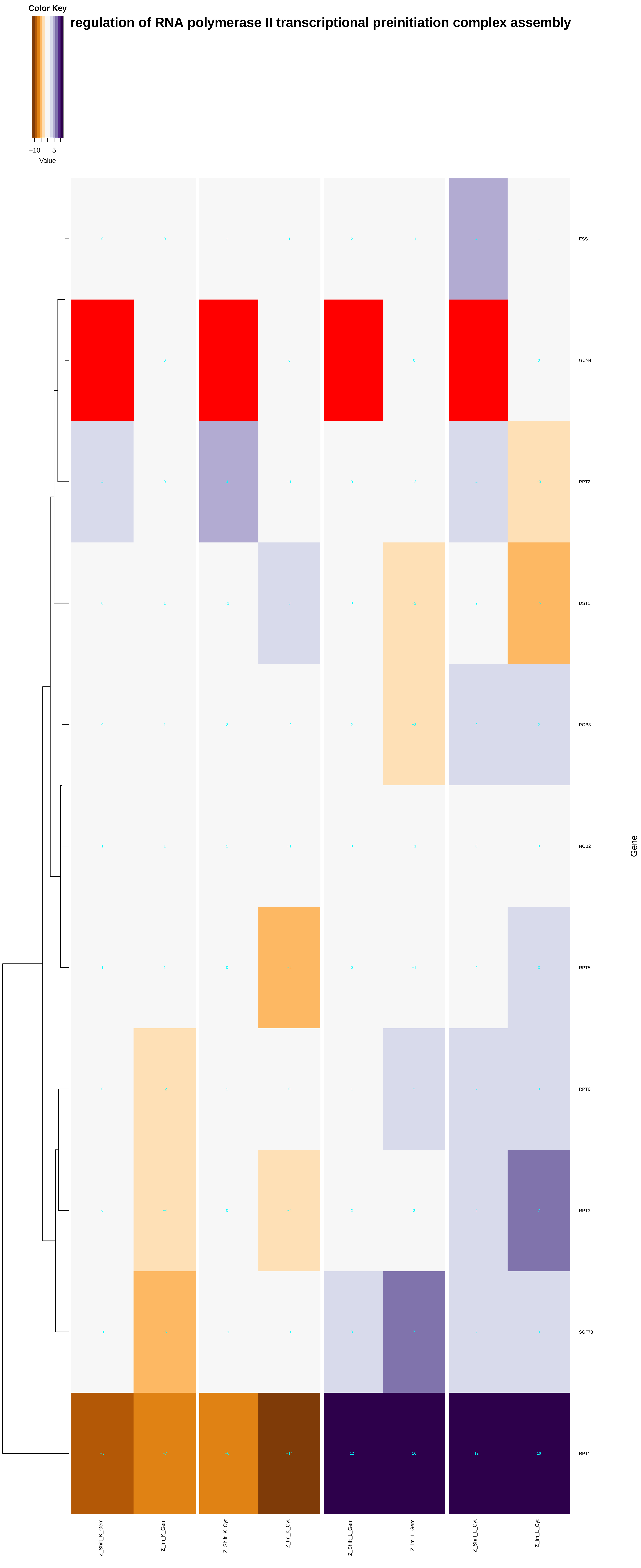
Color Key
−10
5
Value
regulation of RNA polymerase II transcriptional preinitiation complex assembly
ESS1
GCN4
RPT2
DST1
POB3
NCB2
RPT5
RPT6
RPT3
SGF73
RPT1
Gene
Z_Shift_K_Gem
Z_Im_K_Gem
Z_Shift_K_Cyt
Z_Im_K_Cyt
Z_Shift_L_Gem
Z_Im_L_Gem
Z_Shift_L_Cyt
Z_Im_L_Cyt

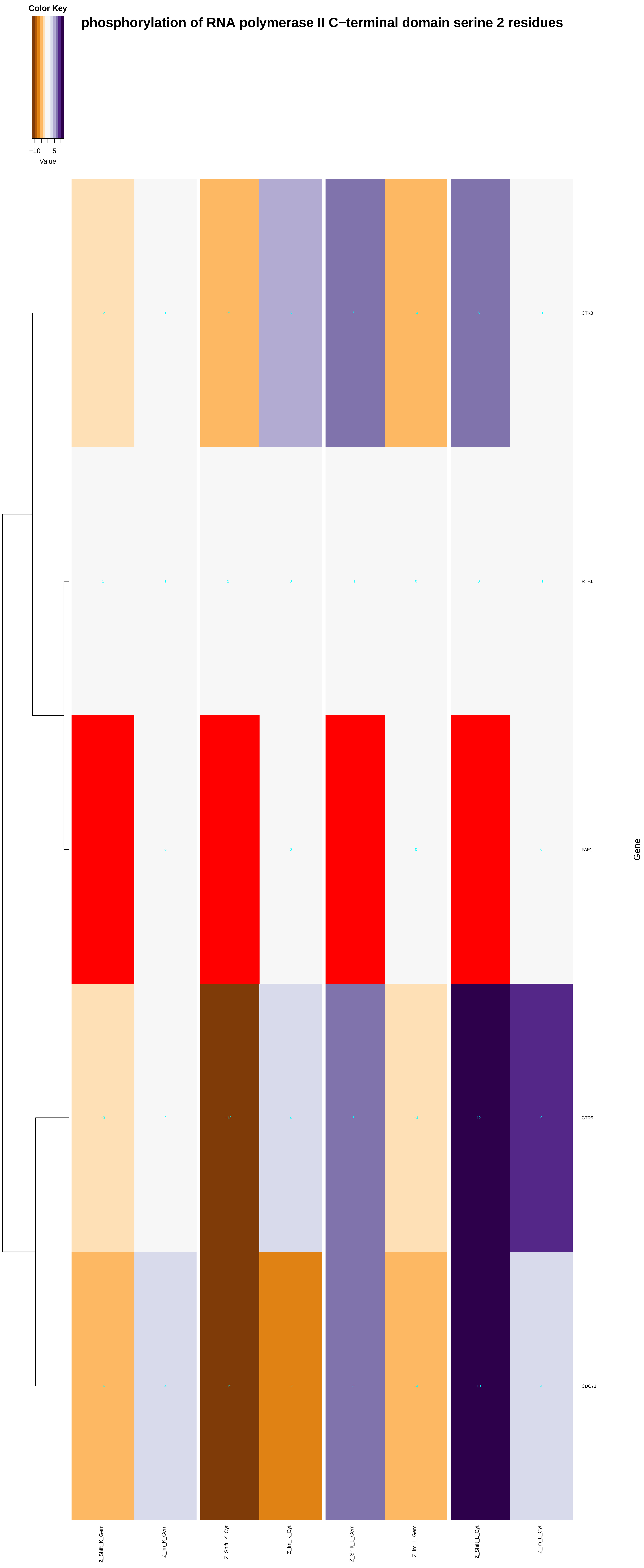
phosphorylation of RNA polymerase II C−terminal domain serine 2 residues
Color Key
−10
5
Value
CTK3
RTF1
PAF1
CTR9
CDC73
Gene
Z_Shift_K_Gem
Z_Im_K_Gem
Z_Shift_K_Cyt
Z_Im_K_Cyt
Z_Shift_L_Gem
Z_Im_L_Gem
Z_Shift_L_Cyt
Z_Im_L_Cyt

atty acid biosynthetic process by regulation of transcription from RNA polymerase II prom

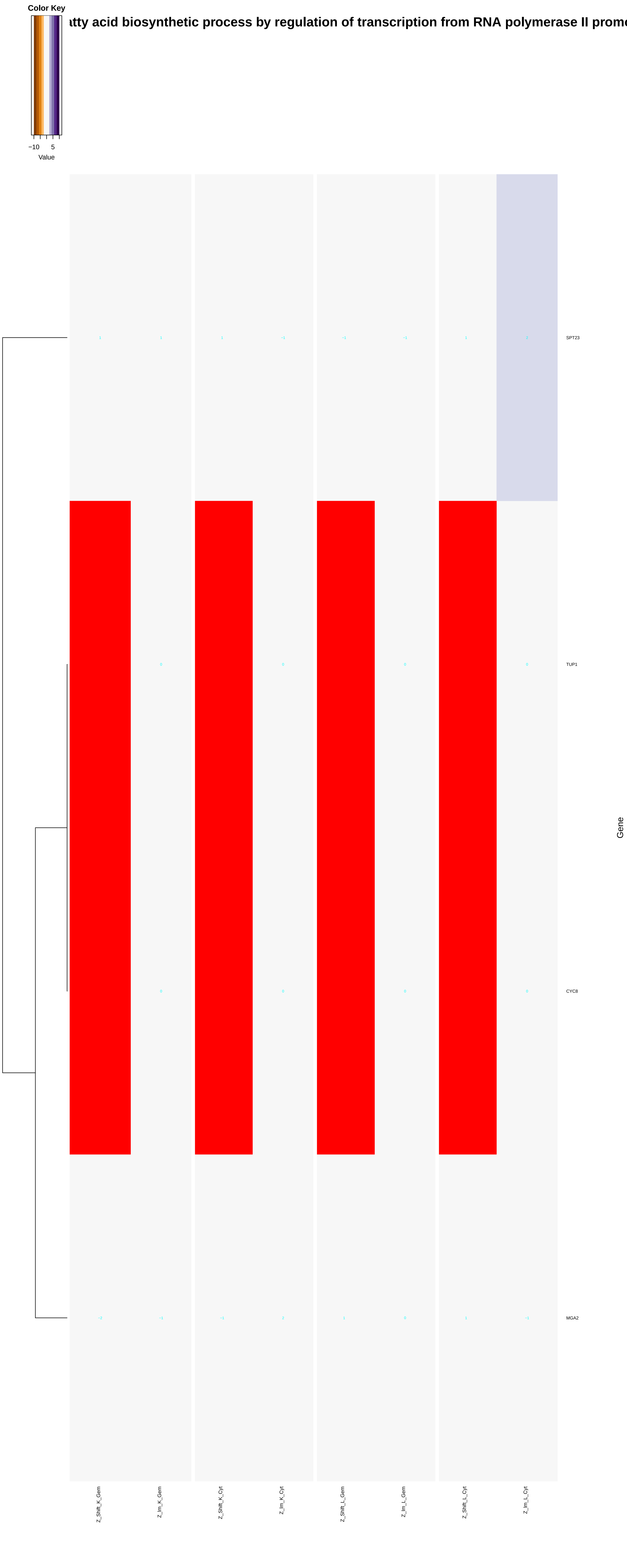

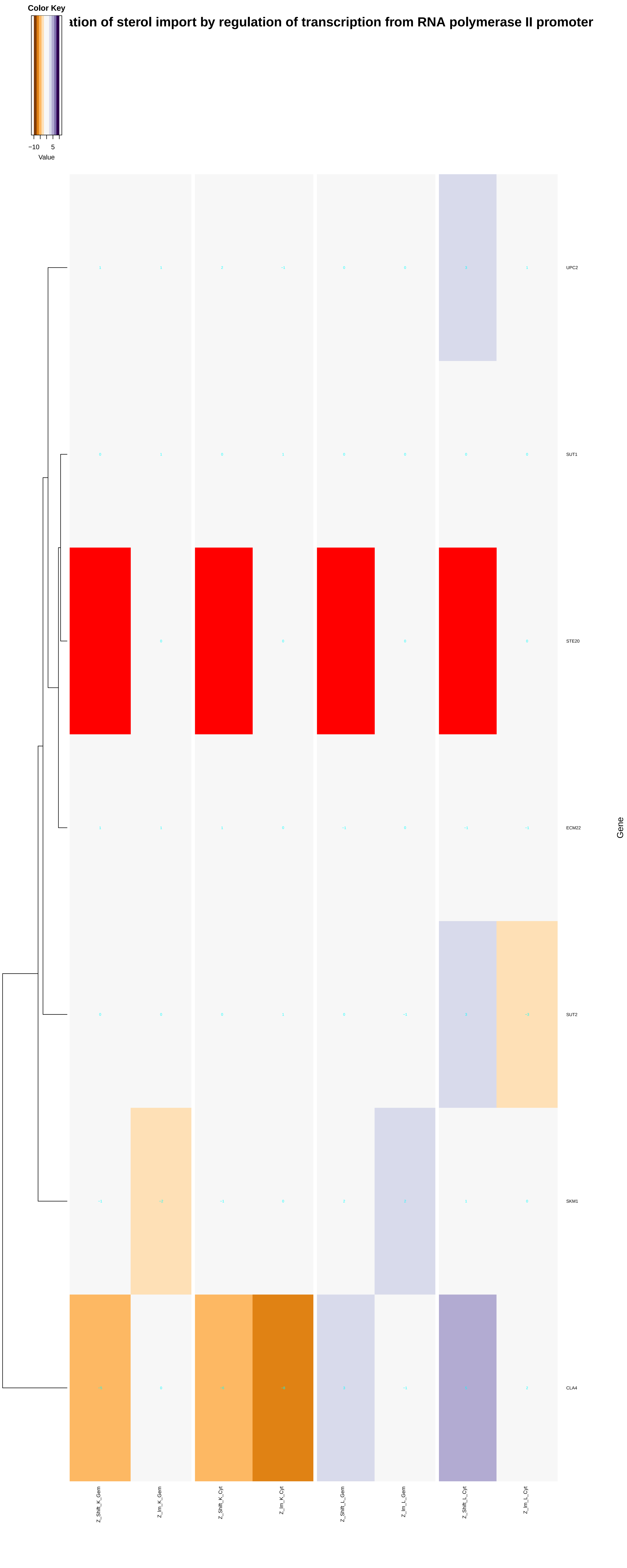
ation of sterol import by regulation of transcription from RNA polymerase II promoter
Color Key
−10
5
Value
UPC2
SUT1
STE20
ECM22
SUT2
SKM1
CLA4
Gene
Z_Shift_K_Gem
Z_Im_K_Gem
Z_Shift_K_Cyt
Z_Im_K_Cyt
Z_Shift_L_Gem
Z_Im_L_Gem
Z_Shift_L_Cyt
Z_Im_L_Cyt

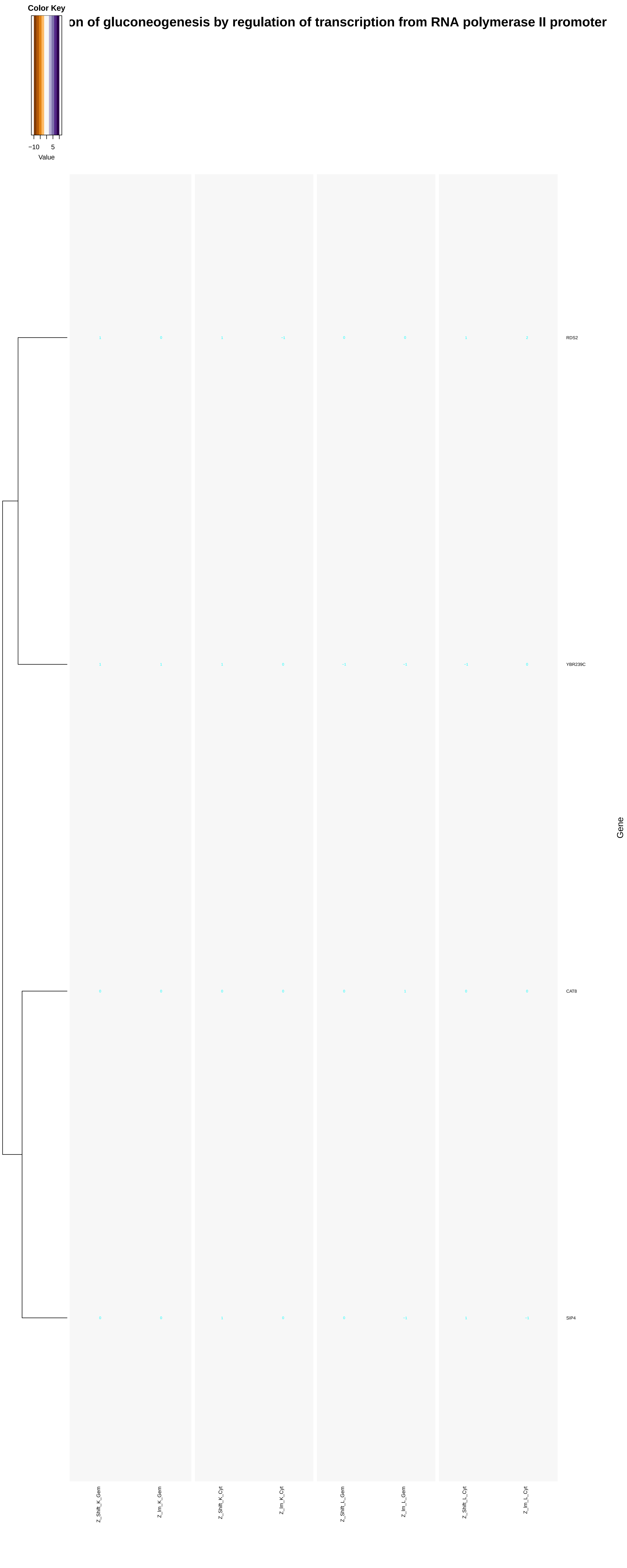

Color Key
−10
5
Value
on of gluconeogenesis by regulation of transcription from RNA polymerase II promoter
1 0 1 −1 0 0 1 2
RDS2
1 1 1 0 −1 −1 −1 0
YBR239C
0 0 0 0 0 1 0 0
CAT8
0 0 1 0 0 −1 1 −1
SIP4
Gene
Z_Shift_K_Gem
Z_Im_K_Gem
Z_Shift_K_Cyt
Z_Im_K_Cyt
Z_Shift_L_Gem
Z_Im_L_Gem
Z_Shift_L_Cyt
Z_Im_L_Cyt

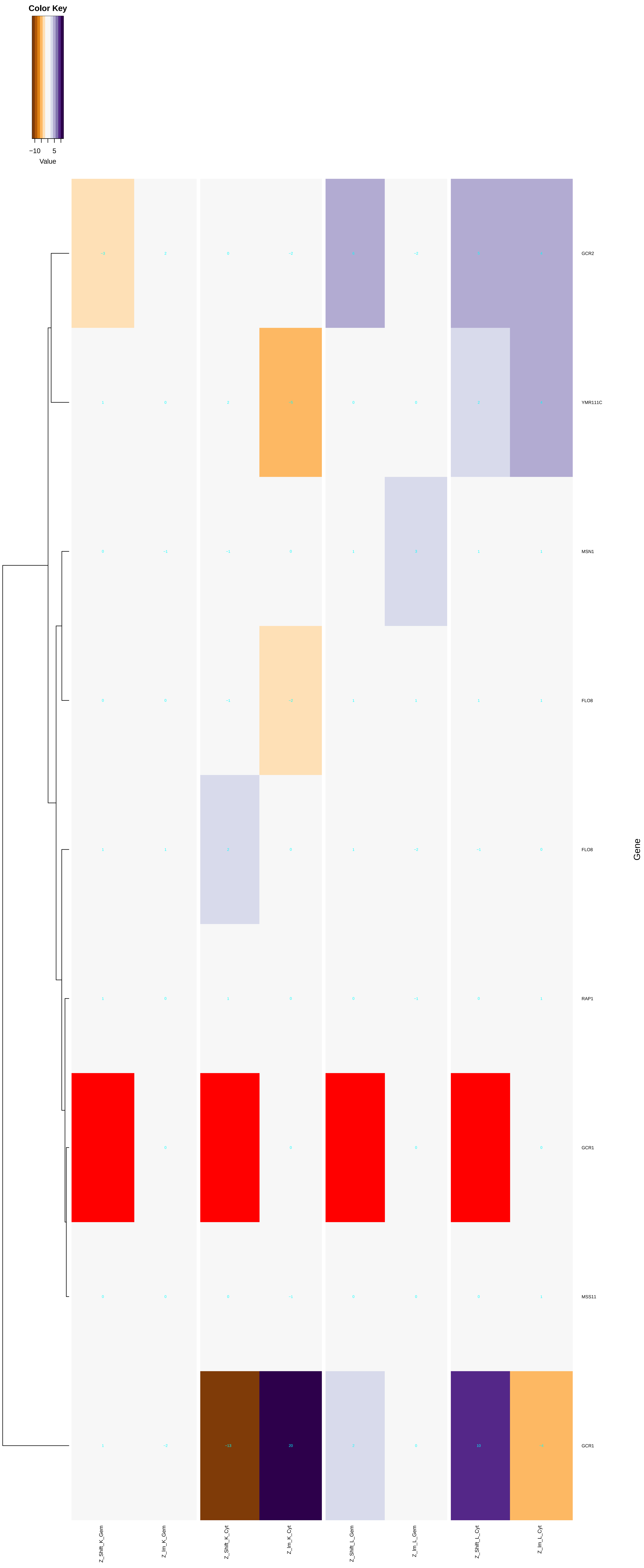

Color Key
−10
5
Value
GCR2
YMR111C
MSN1
FLO8
FLO8
RAP1
GCR1
MSS11
GCR1
Gene
Z_Shift_K_Gem
Z_Im_K_Gem
Z_Shift_K_Cyt
Z_Im_K_Cyt
Z_Shift_L_Gem
Z_Im_L_Gem
Z_Shift_L_Cyt
Z_Im_L_Cyt

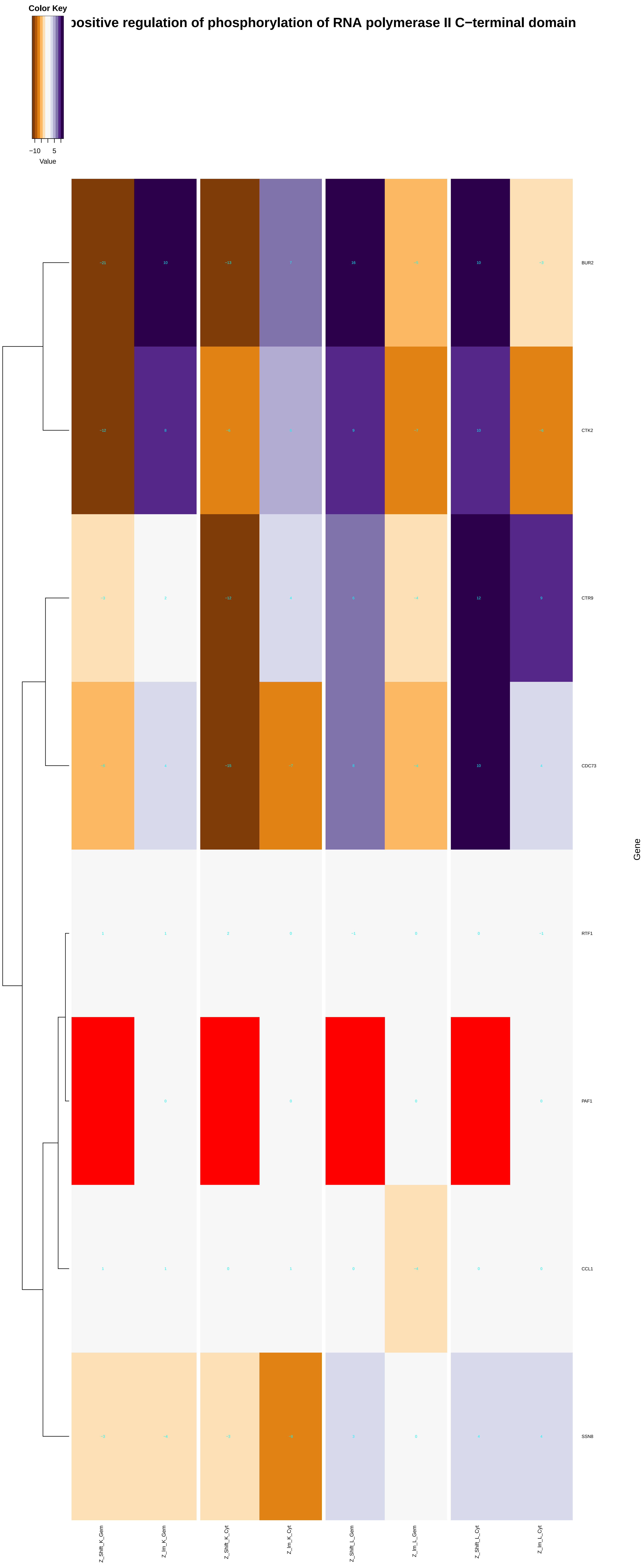
Color Key
−10 5
Value
positive regulation of phosphorylation of RNA polymerase II C−terminal domain
BUR2
CTK2
CTR9
CDC73
RTF1
PAF1
CCL1
SSN8
Gene
Z_Shift_K_Gem
Z_Im_K_Gem
Z_Shift_K_Cyt
Z_Im_K_Cyt
Z_Shift_L_Gem
Z_Im_L_Gem
Z_Shift_L_Cyt
Z_Im_L_Cyt

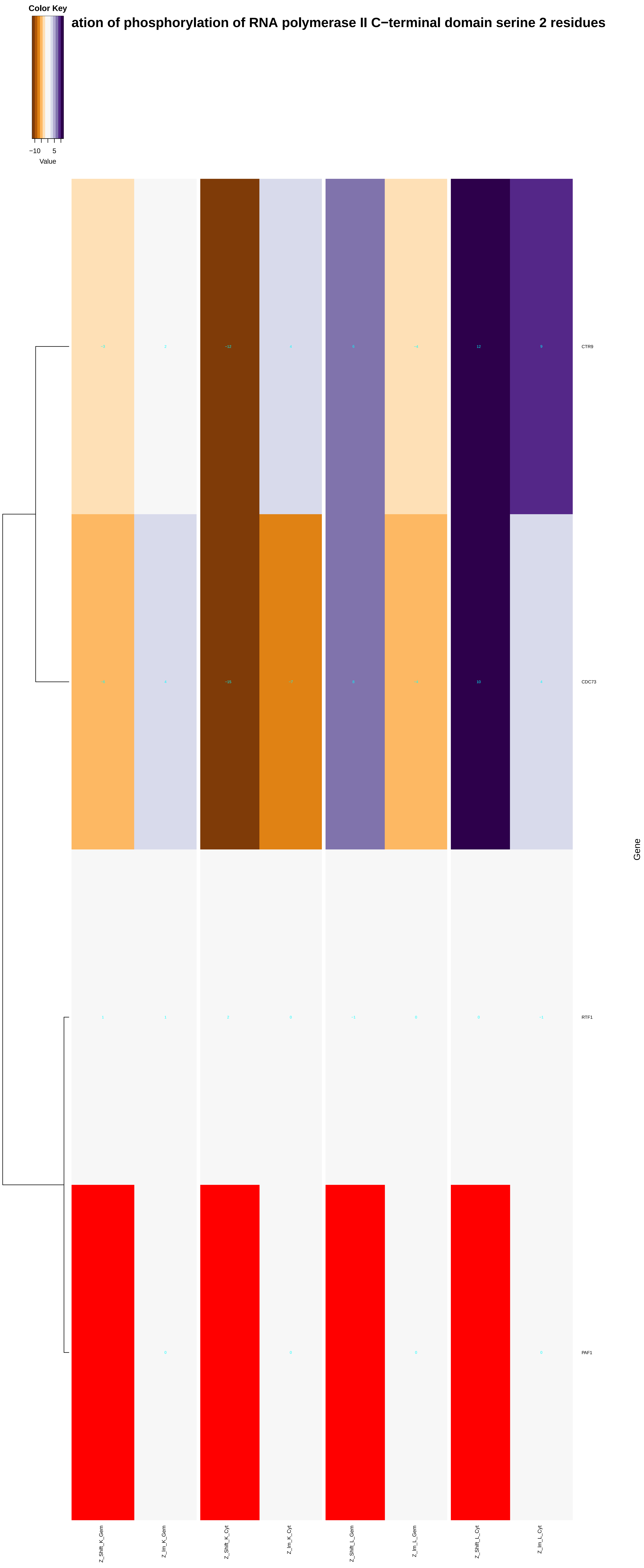
ation of phosphorylation of RNA polymerase II C−terminal domain serine 2 residues
Color Key
−10
5
Value
CTR9
CDC73
RTF1
PAF1
Gene
Z_Shift_K_Gem
Z_Im_K_Gem
Z_Shift_K_Cyt
Z_Im_K_Cyt
Z_Shift_L_Gem
Z_Im_L_Gem
Z_Shift_L_Cyt
Z_Im_L_Cyt

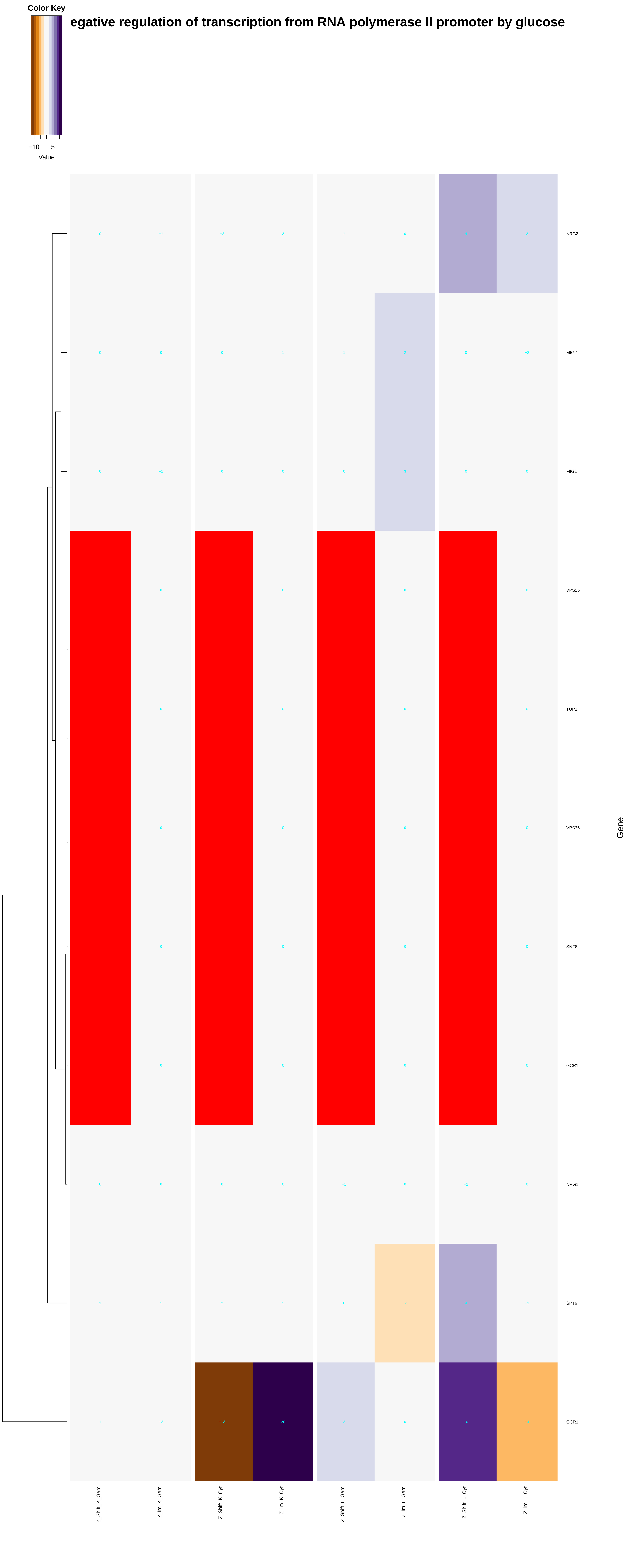
egative regulation of transcription from RNA polymerase II promoter by glucose
Color Key
−10
5
Value
NRG2
MIG2
MIG1
VPS25
TUP1
VPS36
SNF8
GCR1
NRG1
SPT6
GCR1
Gene
Z_Shift_K_Gem
Z_Im_K_Gem
Z_Shift_K_Cyt
Z_Im_K_Cyt
Z_Shift_L_Gem
Z_Im_L_Gem
Z_Shift_L_Cyt
Z_Im_L_Cyt

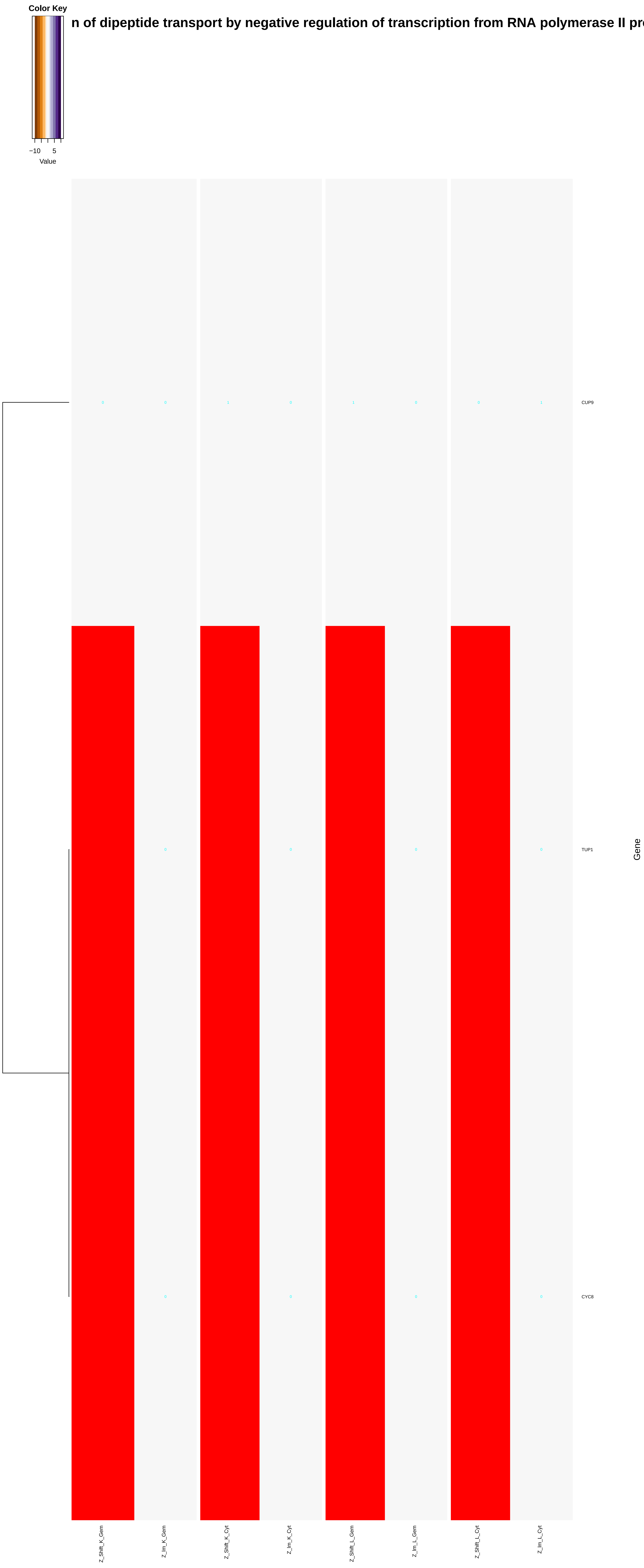
Color Key
−10 5
Value
n of dipeptide transport by negative regulation of transcription from RNA polymerase II pro
CUP9
TUP1
CYC8
Gene
Z_Shift_K_Gem
Z_Im_K_Gem
Z_Shift_K_Cyt
Z_Im_K_Cyt
Z_Shift_L_Gem
Z_Im_L_Gem
Z_Shift_L_Cyt
Z_Im_L_Cyt

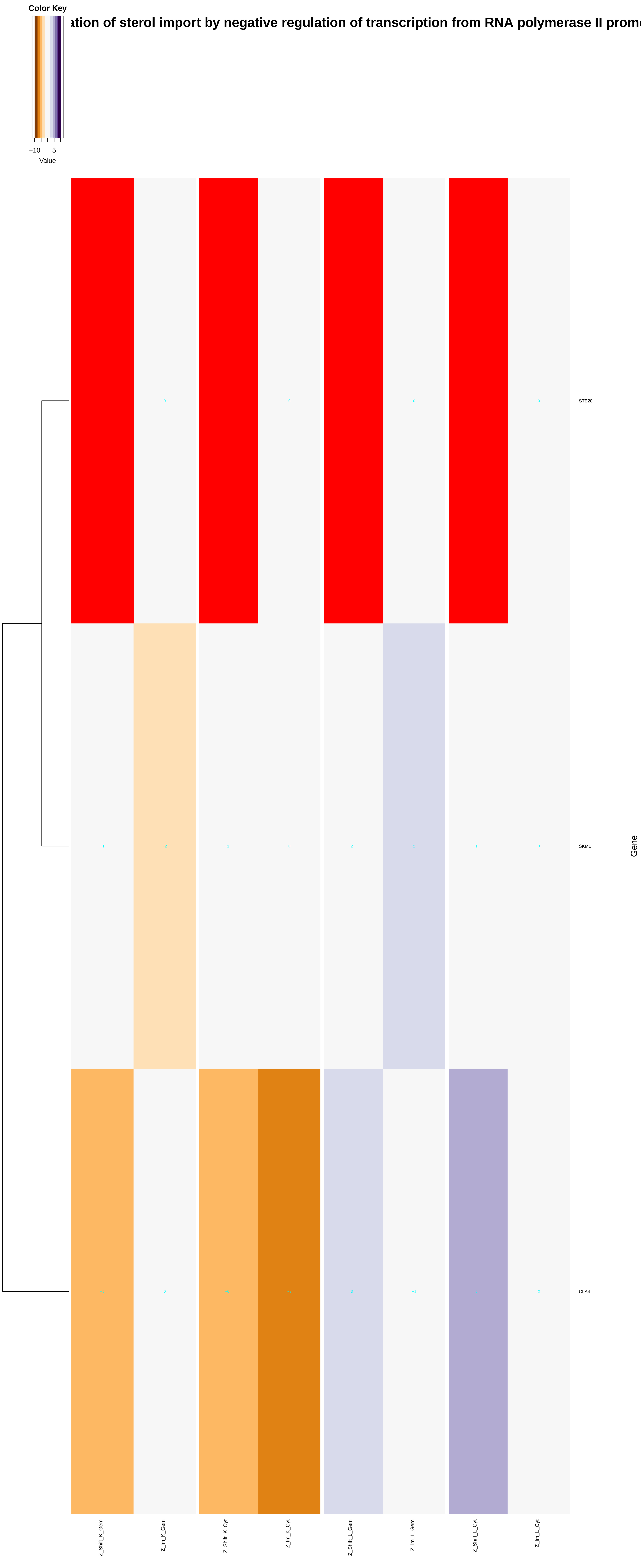

ation of sterol import by negative regulation of transcription from RNA polymerase II prom
Color Key
−10
5
Value
STE20
SKM1
CLA4
Gene
Z_Shift_K_Gem
Z_Im_K_Gem
Z_Shift_K_Cyt
Z_Im_K_Cyt
Z_Shift_L_Gem
Z_Im_L_Gem
Z_Shift_L_Cyt
Z_Im_L_Cyt

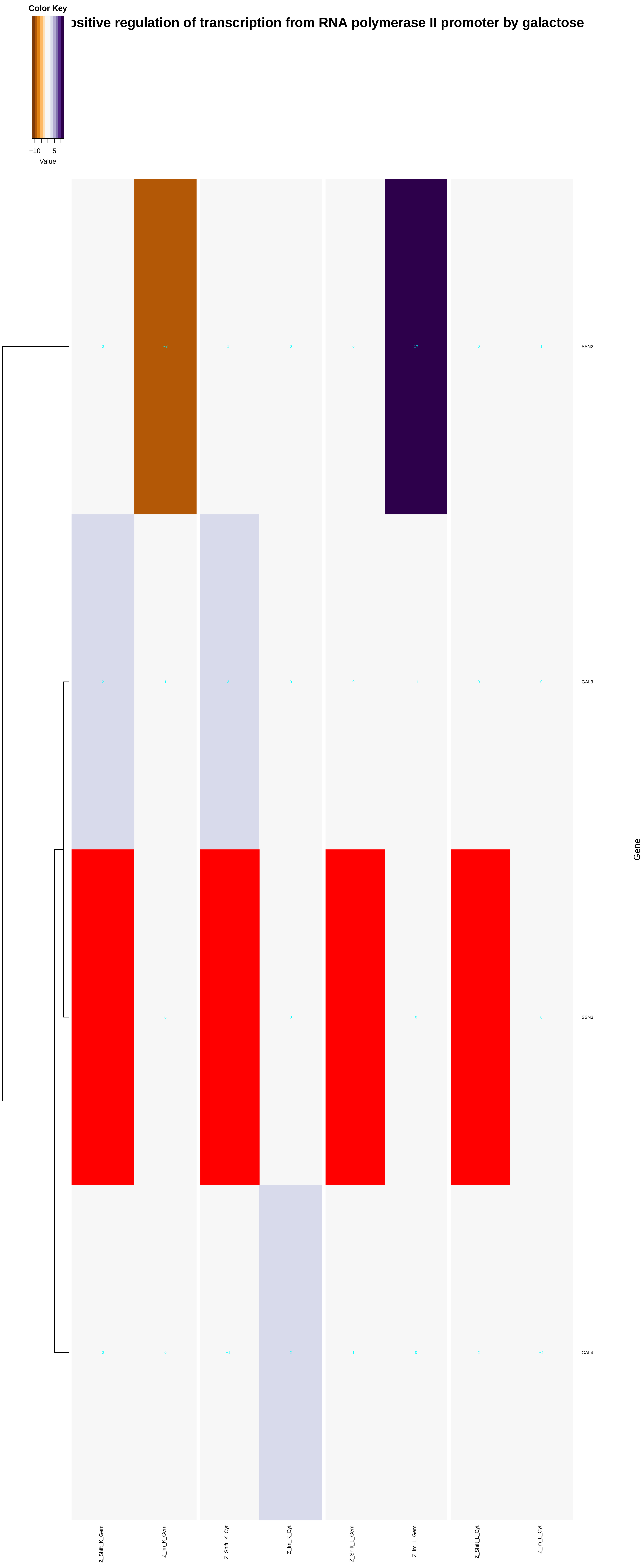
Color Key
−10
5
Value
ositive regulation of transcription from RNA polymerase II promoter by galactose
SSN2
GAL3
SSN3
GAL4
Gene
Z_Shift_K_Gem
Z_Im_K_Gem
Z_Shift_K_Cyt
Z_Im_K_Cyt
Z_Shift_L_Gem
Z_Im_L_Gem
Z_Shift_L_Cyt
Z_Im_L_Cyt

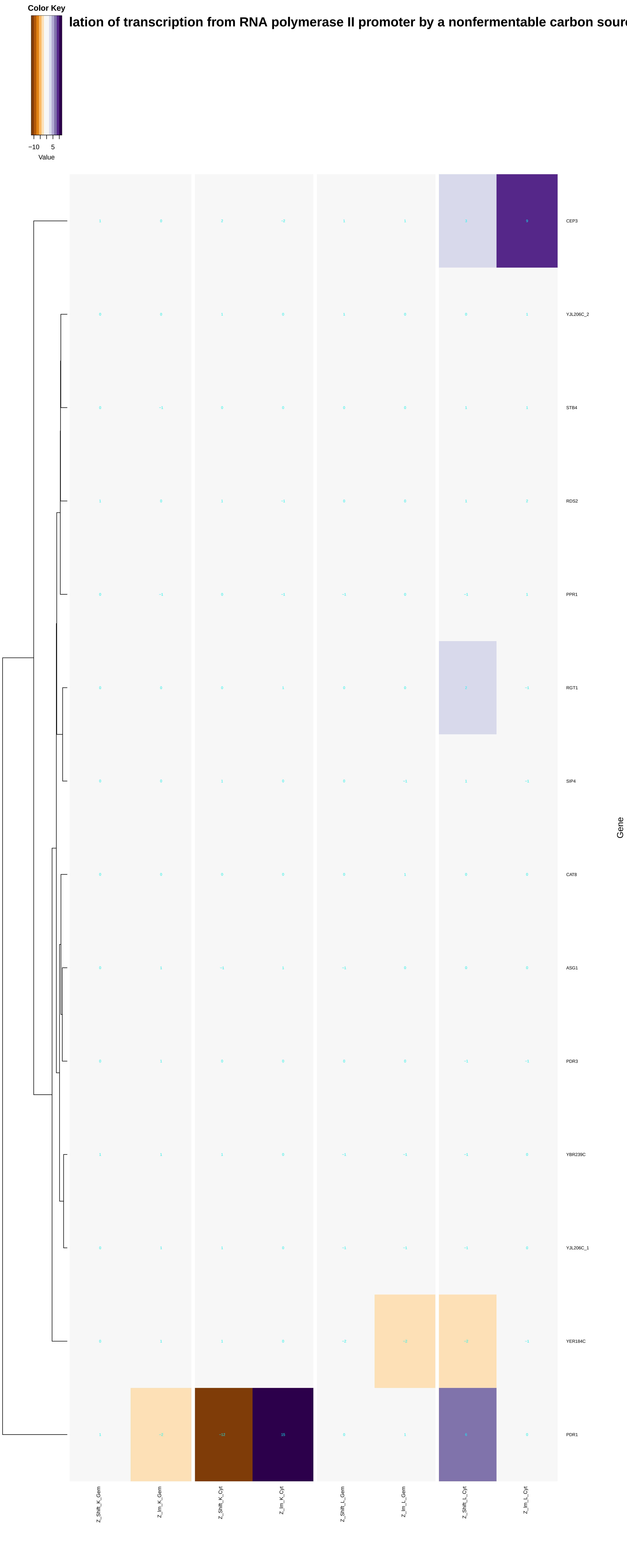
lation of transcription from RNA polymerase II promoter by a nonfermentable carbon sourc
Color Key
−10
5
Value
CEP3
YJL206C_2
STB4
RDS2
PPR1
RGT1
SIP4
CAT8
ASG1
PDR3
YBR239C
YJL206C_1
YER184C
PDR1
Gene
Z_Shift_K_Gem
Z_Im_K_Gem
Z_Shift_K_Cyt
Z_Im_K_Cyt
Z_Shift_L_Gem
Z_Im_L_Gem
Z_Shift_L_Cyt
Z_Im_L_Cyt

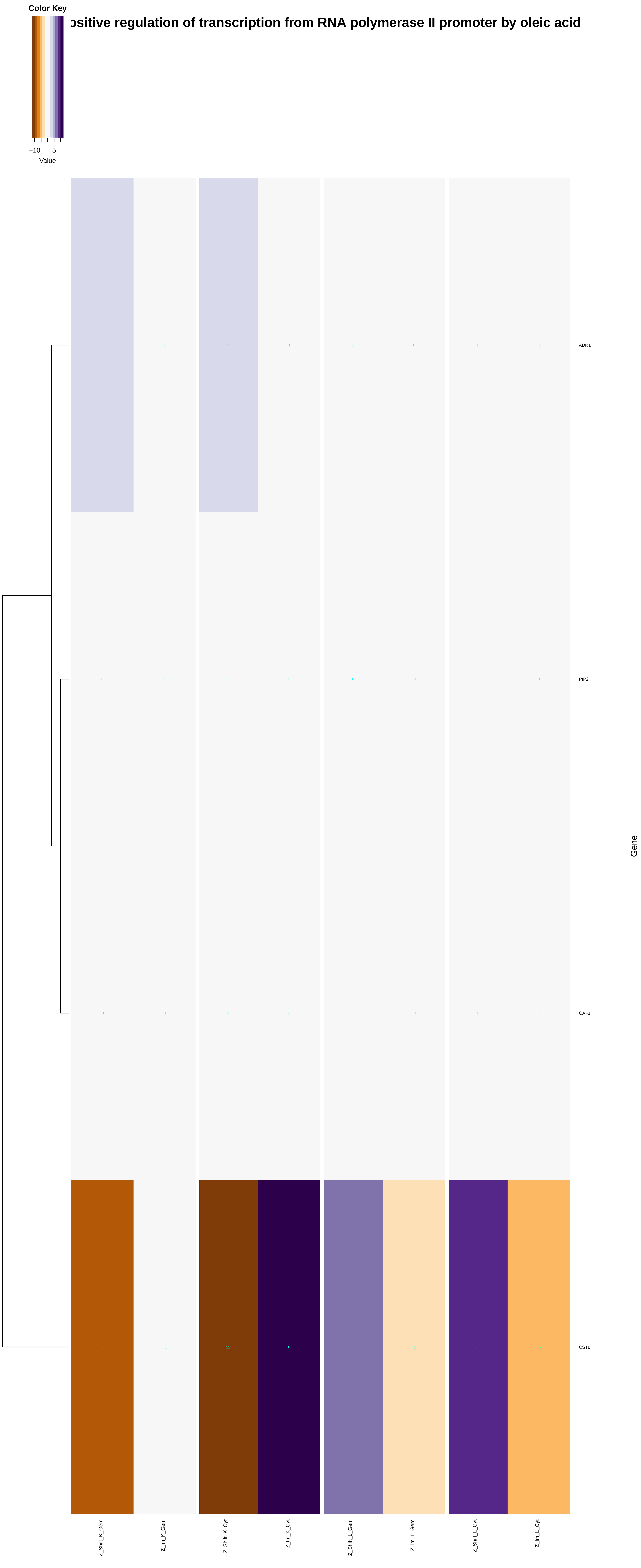
ositive regulation of transcription from RNA polymerase II promoter by oleic acid
Color Key
−10
5
Value
ADR1
PIP2
OAF1
CST6
Gene
Z_Shift_K_Gem
Z_Im_K_Gem
Z_Shift_K_Cyt
Z_Im_K_Cyt
Z_Shift_L_Gem
Z_Im_L_Gem
Z_Shift_L_Cyt
Z_Im_L_Cyt

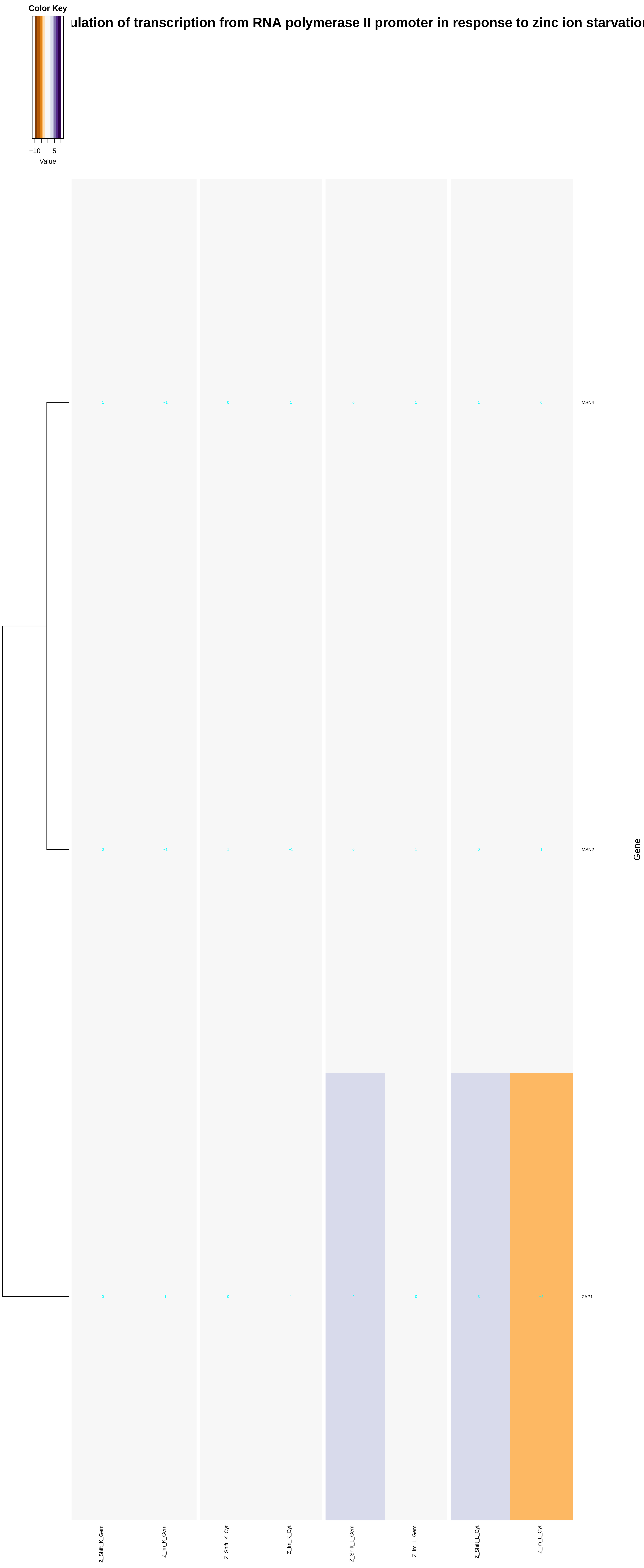

ulation of transcription from RNA polymerase II promoter in response to zinc ion starvatio
Color Key
−10
5
Value
MSN4
MSN2
ZAP1
Gene
Z_Shift_K_Gem
Z_Im_K_Gem
Z_Shift_K_Cyt
Z_Im_K_Cyt
Z_Shift_L_Gem
Z_Im_L_Gem
Z_Shift_L_Cyt
Z_Im_L_Cyt

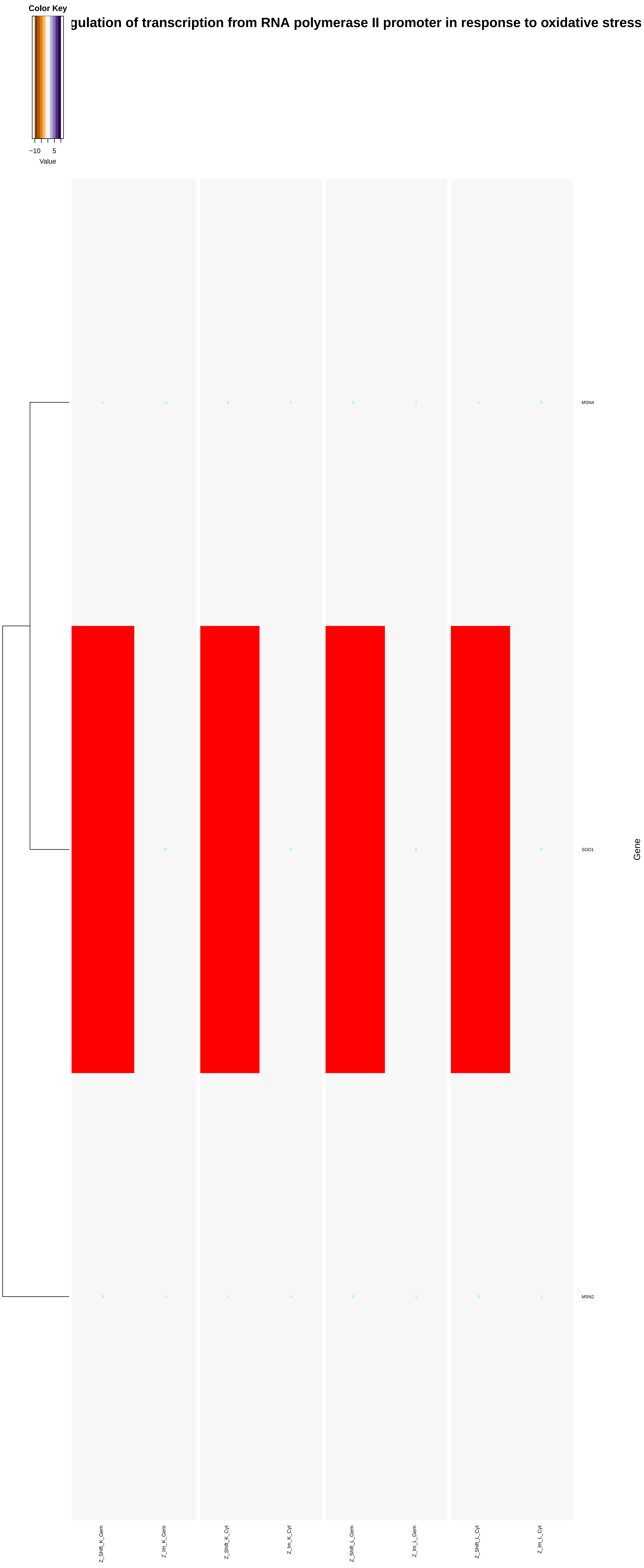
gulation of transcription from RNA polymerase II promoter in response to oxidative stress
Color Key
−10
5
Value
MSN4
SOD1
MSN2
Gene
Z_Shift_K_Gem
Z_Im_K_Gem
Z_Shift_K_Cyt
Z_Im_K_Cyt
Z_Shift_L_Gem
Z_Im_L_Gem
Z_Shift_L_Cyt
Z_Im_L_Cyt

gulation of transcription from RNA polymerase II promoter in response to osmotic stress

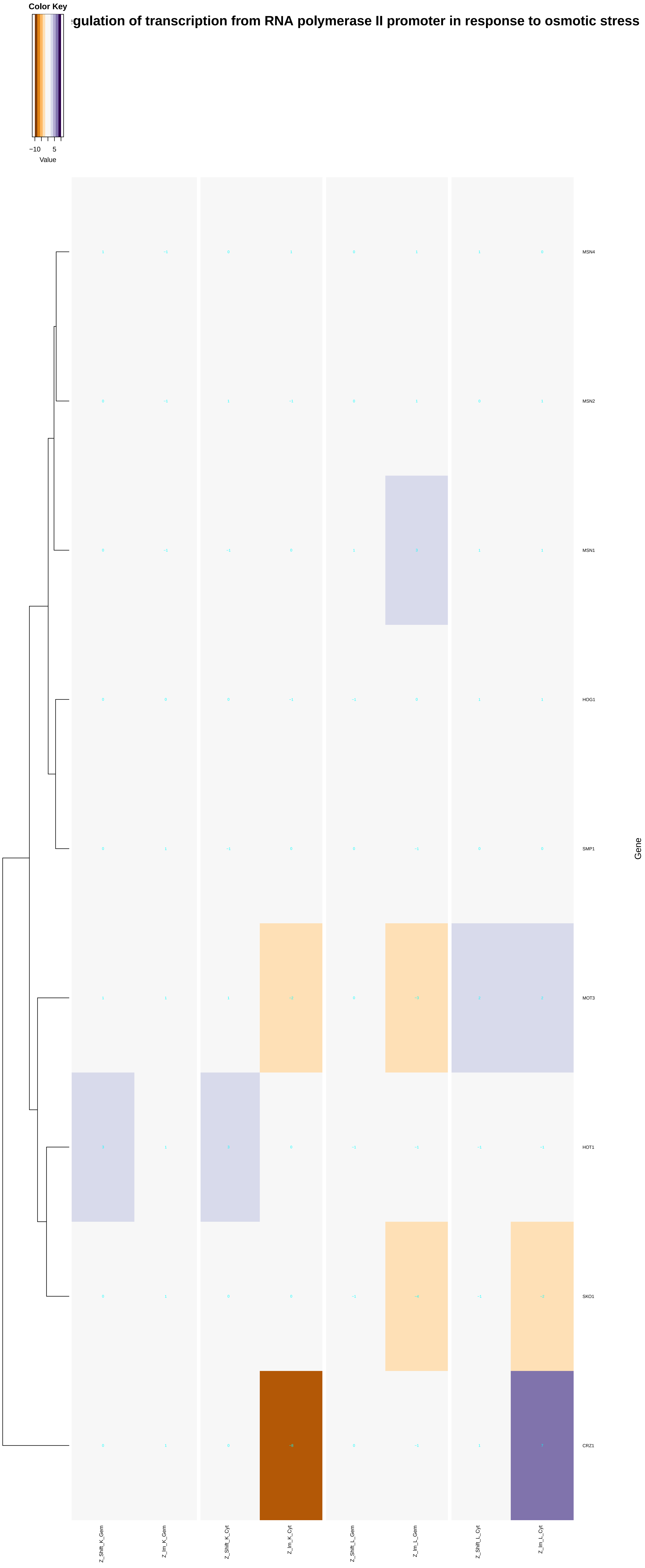

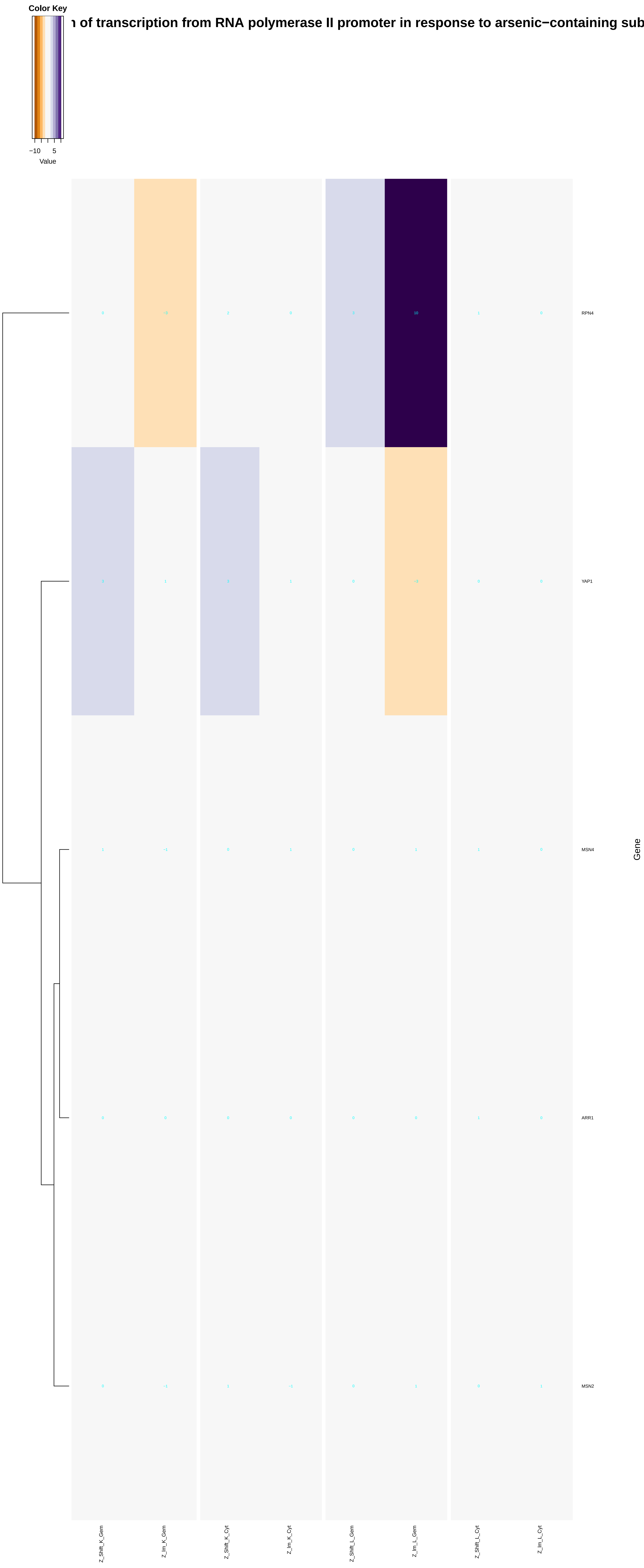

n of transcription from RNA polymerase II promoter in response to arsenic−containing sub
Color Key
−10
5
Value
RPN4
YAP1
MSN4
ARR1
MSN2
Gene
Z_Shift_K_Gem
Z_Im_K_Gem
Z_Shift_K_Cyt
Z_Im_K_Cyt
Z_Shift_L_Gem
Z_Im_L_Gem
Z_Shift_L_Cyt
Z_Im_L_Cyt

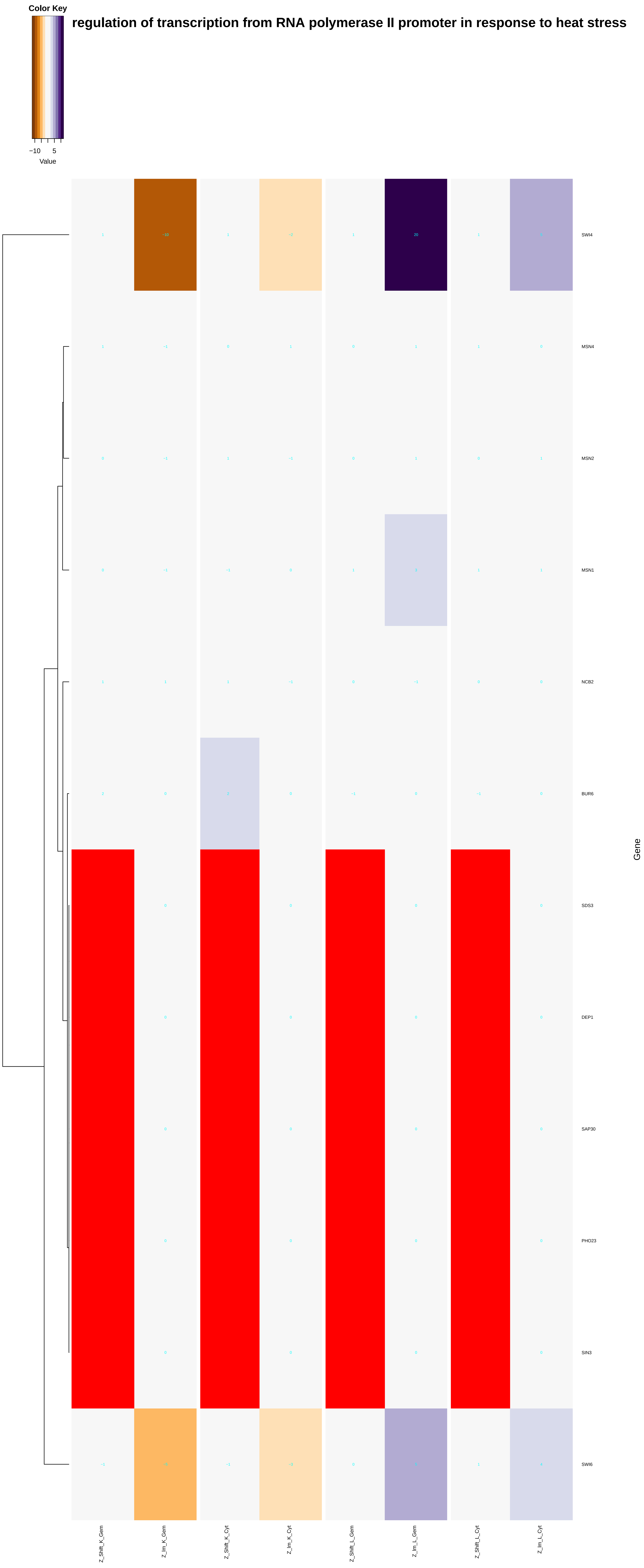
Color Key
−10
5
Value
regulation of transcription from RNA polymerase II promoter in response to heat stress
SWI4
MSN4
MSN2
MSN1
NCB2
BUR6
SDS3
DEP1
SAP30
PHO23
SIN3
SWI6
Gene
Z_Shift_K_Gem
Z_Im_K_Gem
Z_Shift_K_Cyt
Z_Im_K_Cyt
Z_Shift_L_Gem
Z_Im_L_Gem
Z_Shift_L_Cyt
Z_Im_L_Cyt

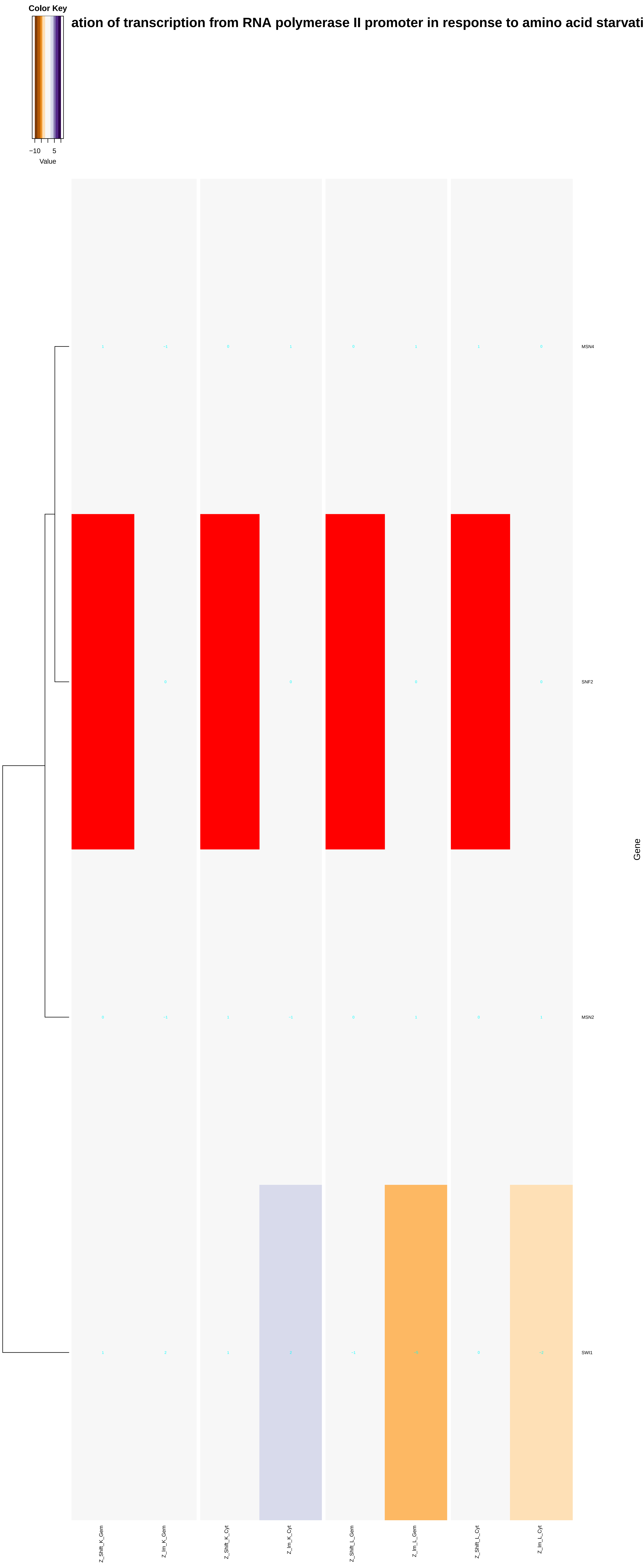

Color Key
−10
5
Value
ation of transcription from RNA polymerase II promoter in response to amino acid starvati
MSN4
SNF2
MSN2
SWI1
Gene
Z_Shift_K_Gem
Z_Im_K_Gem
Z_Shift_K_Cyt
Z_Im_K_Cyt
Z_Shift_L_Gem
Z_Im_L_Gem
Z_Shift_L_Cyt
Z_Im_L_Cyt

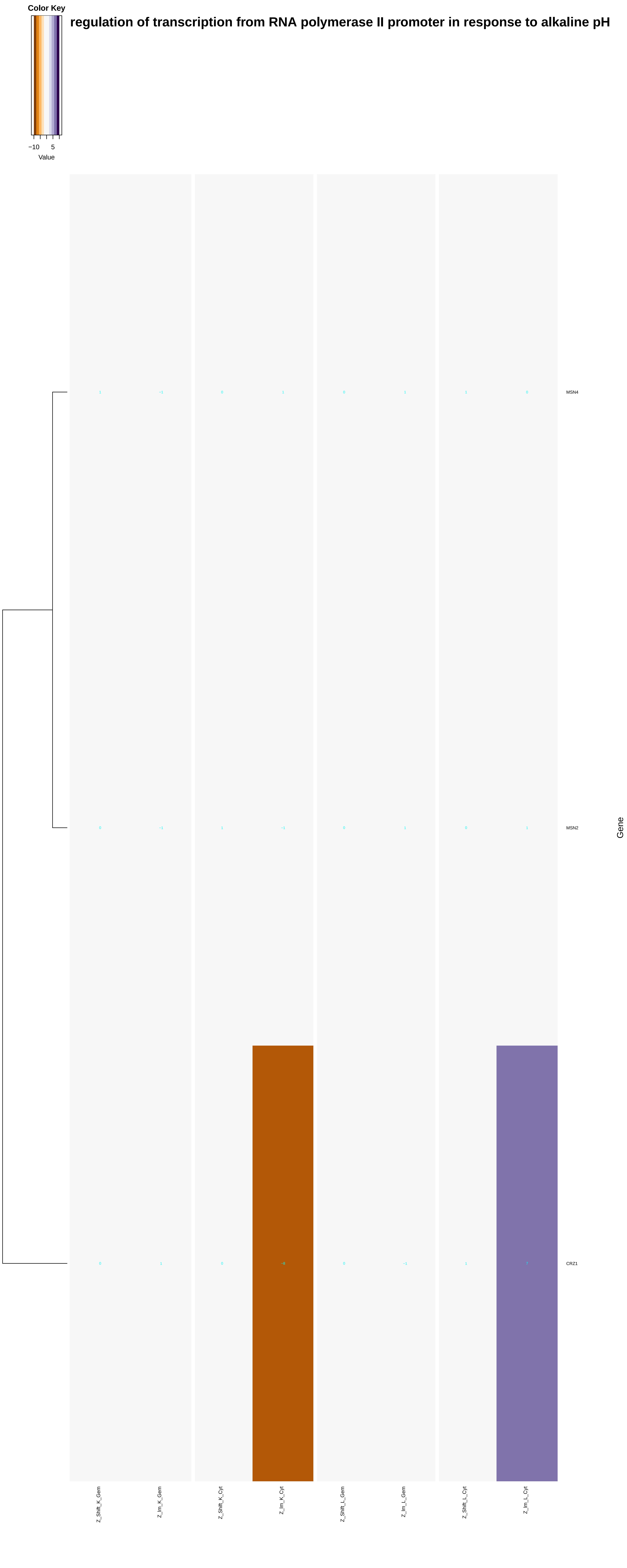
Color Key
−10
5
Value
regulation of transcription from RNA polymerase II promoter in response to alkaline pH
MSN4
MSN2
CRZ1
Gene
Z_Shift_K_Gem
Z_Im_K_Gem
Z_Shift_K_Cyt
Z_Im_K_Cyt
Z_Shift_L_Gem
Z_Im_L_Gem
Z_Shift_L_Cyt
Z_Im_L_Cyt

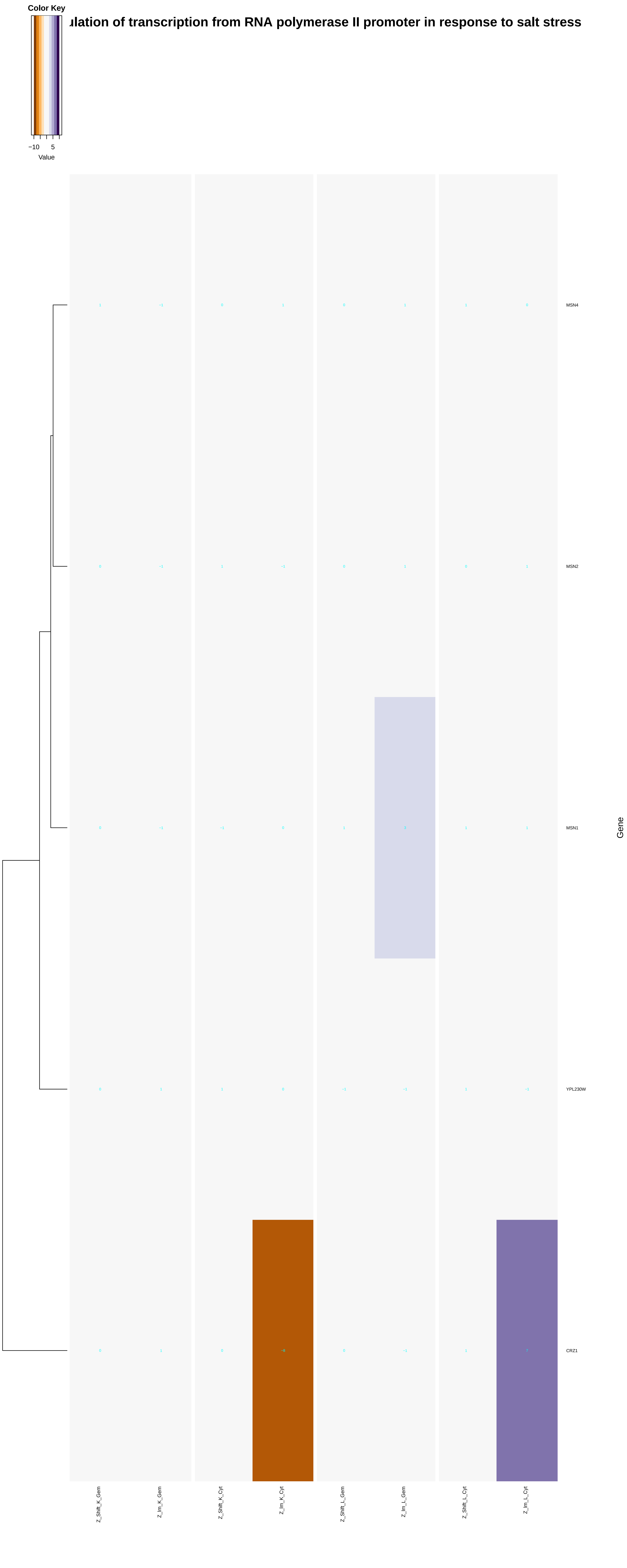
Color Key
−10
5
Value
ulation of transcription from RNA polymerase II promoter in response to salt stress
MSN4
MSN2
MSN1
YPL230W
CRZ1
Gene
Z_Shift_K_Gem
Z_Im_K_Gem
Z_Shift_K_Cyt
Z_Im_K_Cyt
Z_Shift_L_Gem
Z_Im_L_Gem
Z_Shift_L_Cyt
Z_Im_L_Cyt

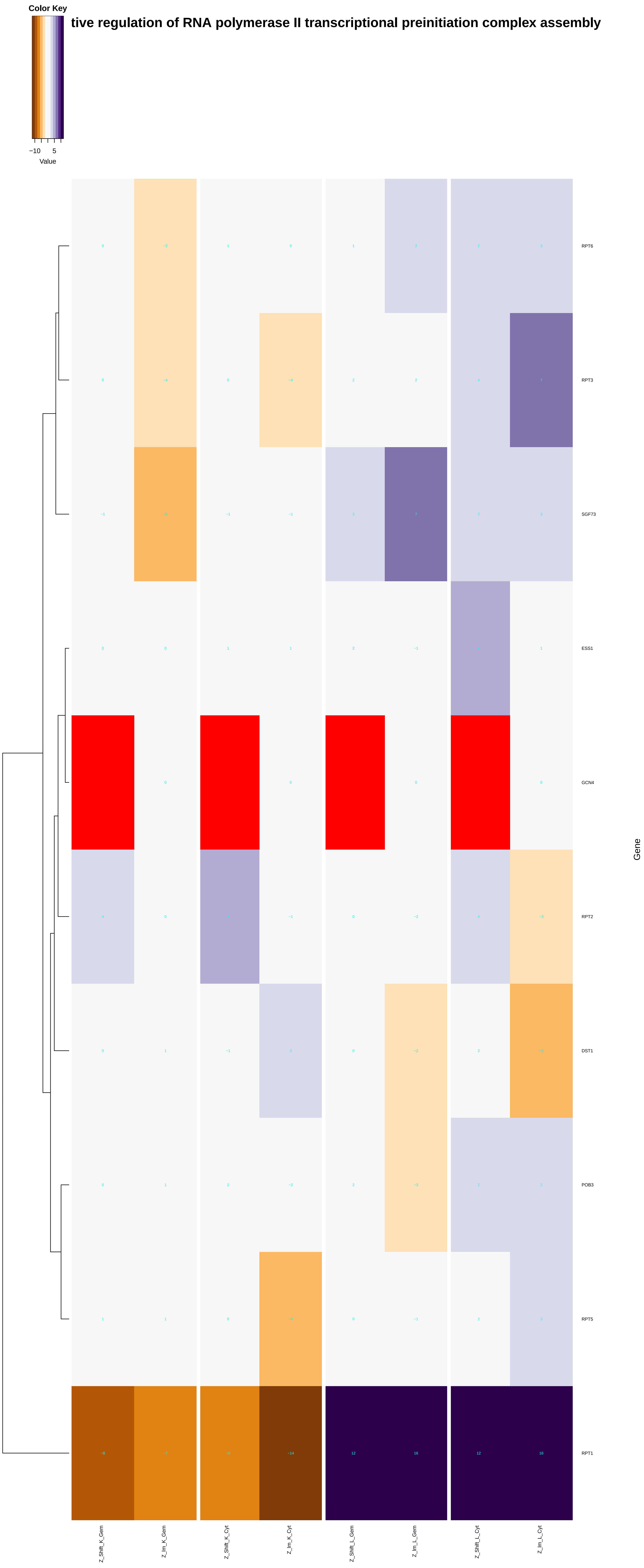
Color Key
−10
5
Value
tive regulation of RNA polymerase II transcriptional preinitiation complex assembly
RPT6
RPT3
SGF73
ESS1
GCN4
RPT2
DST1
POB3
RPT5
RPT1
Gene
Z_Shift_K_Gem
Z_Im_K_Gem
Z_Shift_K_Cyt
Z_Im_K_Cyt
Z_Shift_L_Gem
Z_Im_L_Gem
Z_Shift_L_Cyt
Z_Im_L_Cyt

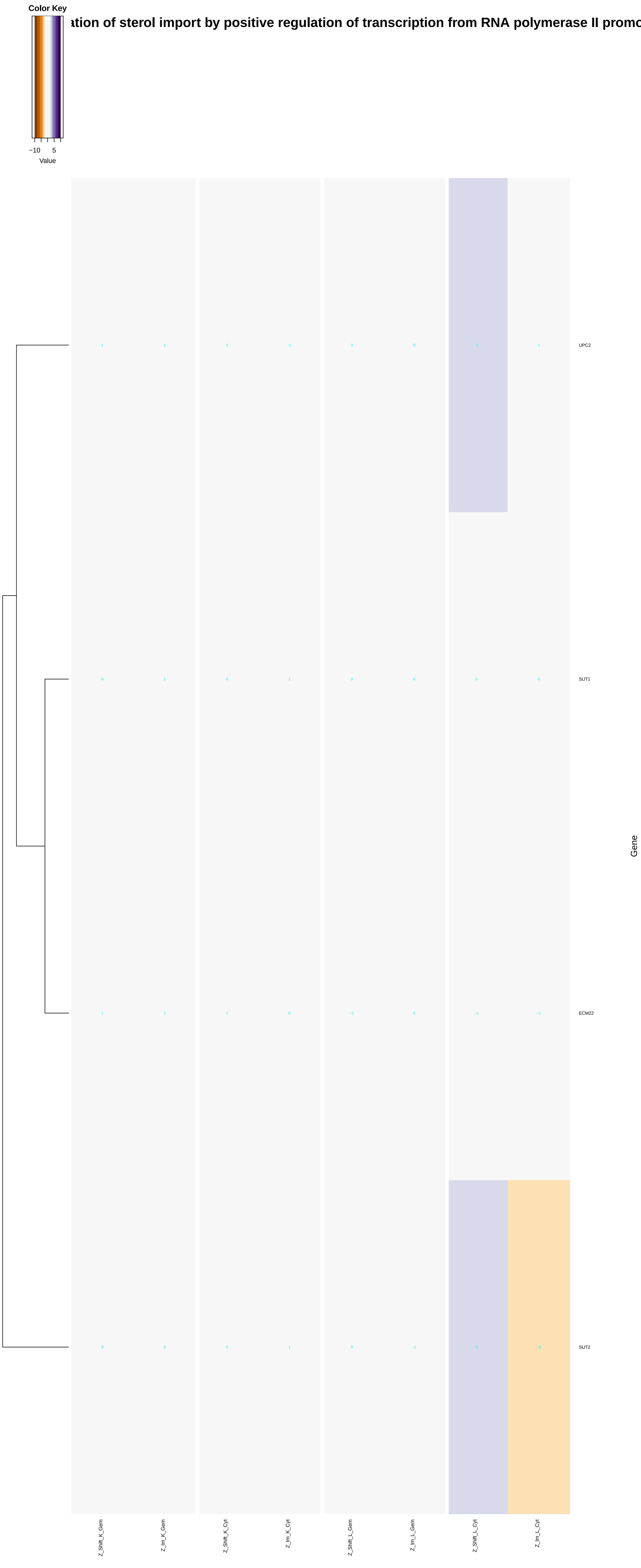
ation of sterol import by positive regulation of transcription from RNA polymerase II promo
Color Key
−10
5
Value
UPC2
SUT1
ECM22
SUT2
Gene
Z_Shift_K_Gem
Z_Im_K_Gem
Z_Shift_K_Cyt
Z_Im_K_Cyt
Z_Shift_L_Gem
Z_Im_L_Gem
Z_Shift_L_Cyt
Z_Im_L_Cyt

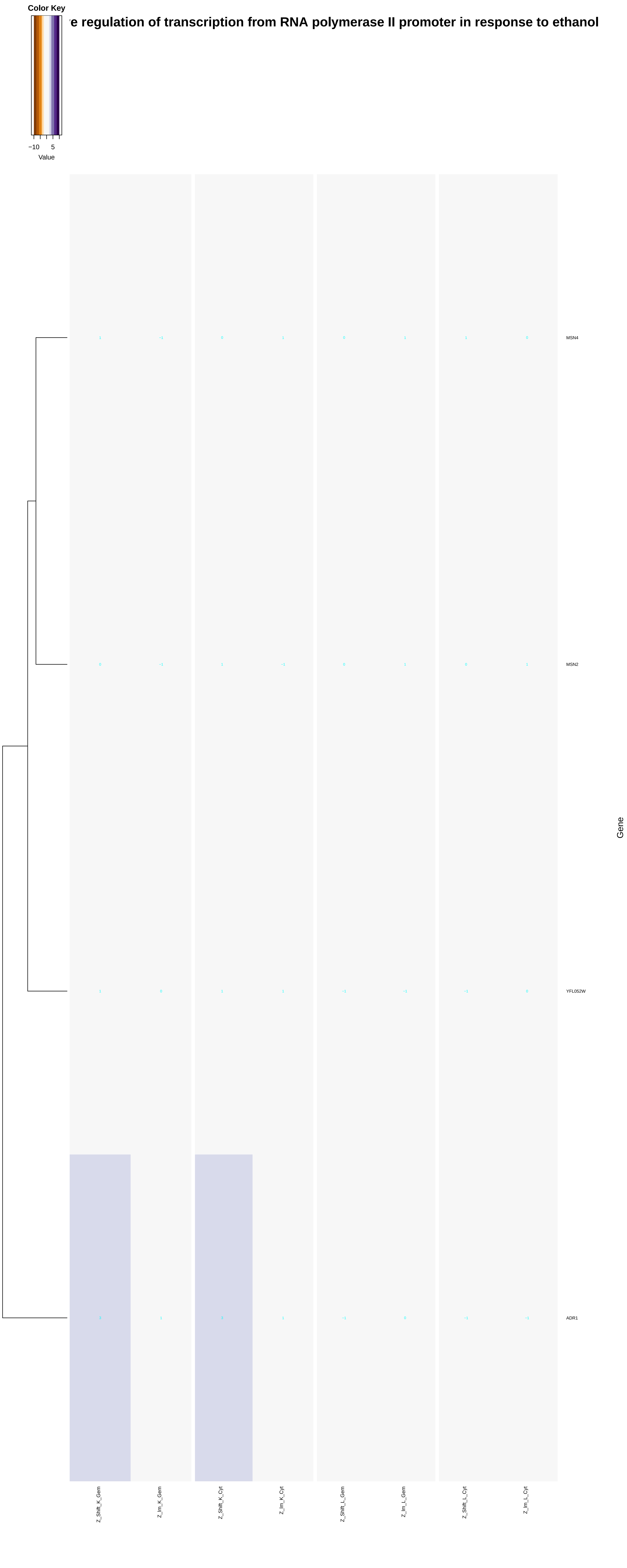

Color Key
−10
5
Value
e regulation of transcription from RNA polymerase II promoter in response to ethanol
MSN4
MSN2
YFL052W
ADR1
Gene
Z_Shift_K_Gem
Z_lm_K_Gem
Z_Shift_K_Cyt
Z_lm_K_Cyt
Z_Shift_L_Gem
Z_lm_L_Gem
Z_Shift_L_Cyt
Z_lm_L_Cyt

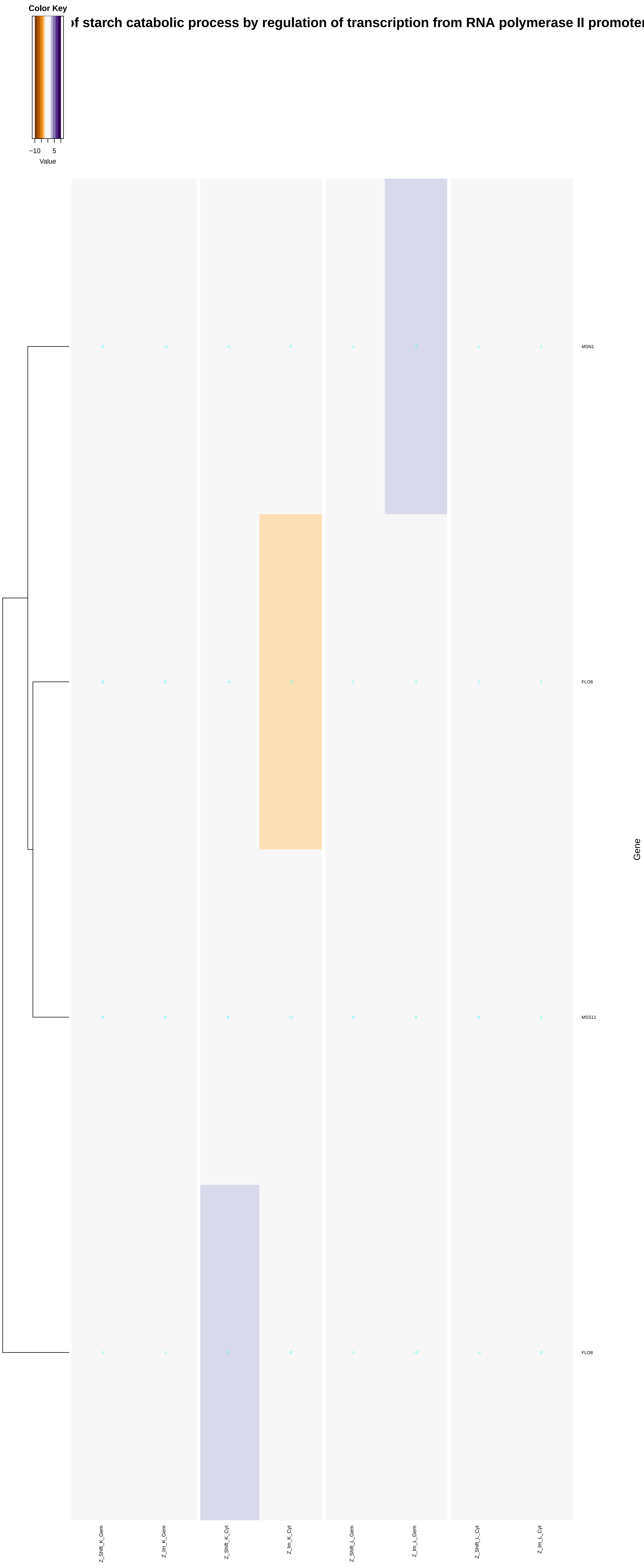

of starch catabolic process by regulation of transcription from RNA polymerase II promoter
Color Key
−10
5
Value
MSN1
FLO8
MSS11
FLO8
Gene
Z_Shift_K_Gem
Z_Im_K_Gem
Z_Shift_K_Cyt
Z_Im_K_Cyt
Z_Shift_L_Gem
Z_Im_L_Gem
Z_Shift_L_Cyt
Z_Im_L_Cyt

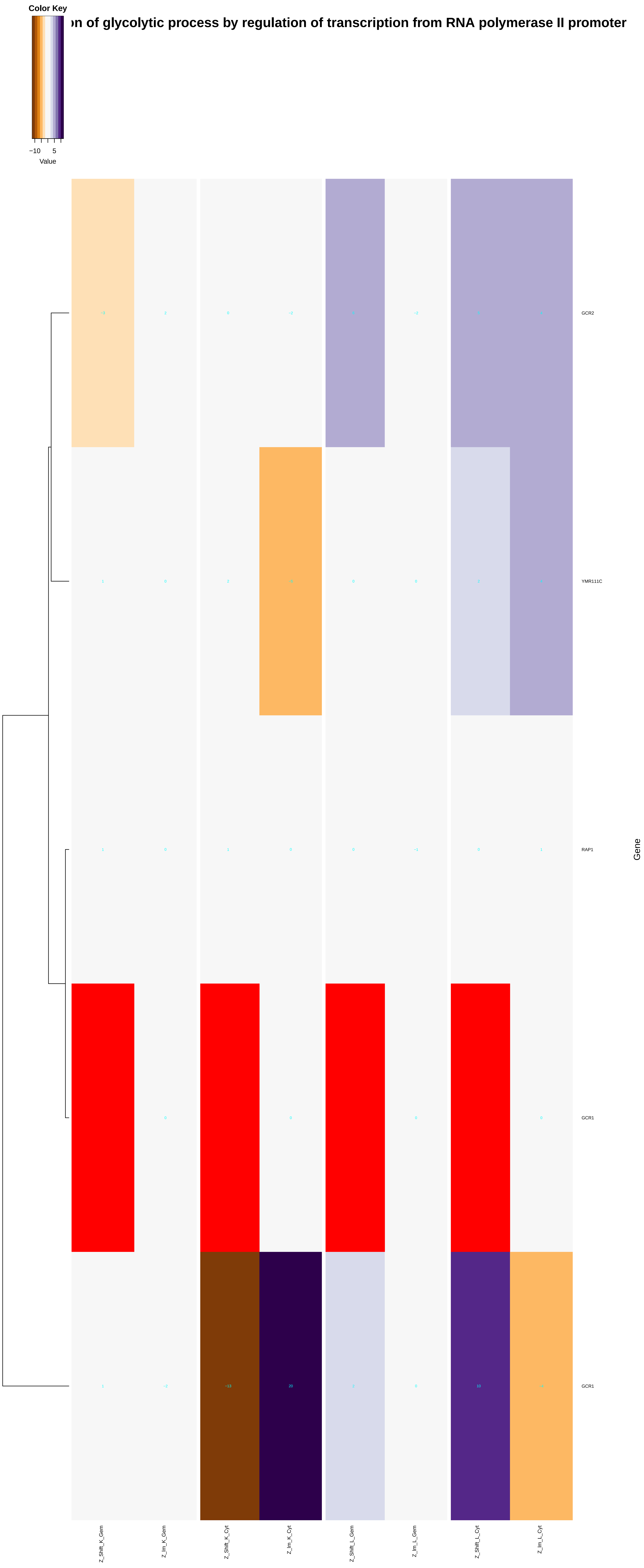
on of glycolytic process by regulation of transcription from RNA polymerase II promoter
Color Key
−10
5
Value
GCR2
YMR111C
RAP1
GCR1
GCR1
Gene
Z_Shift_K_Gem
Z_Im_K_Gem
Z_Shift_K_Cyt
Z_Im_K_Cyt
Z_Shift_L_Gem
Z_Im_L_Gem
Z_Shift_L_Cyt
Z_Im_L_Cyt

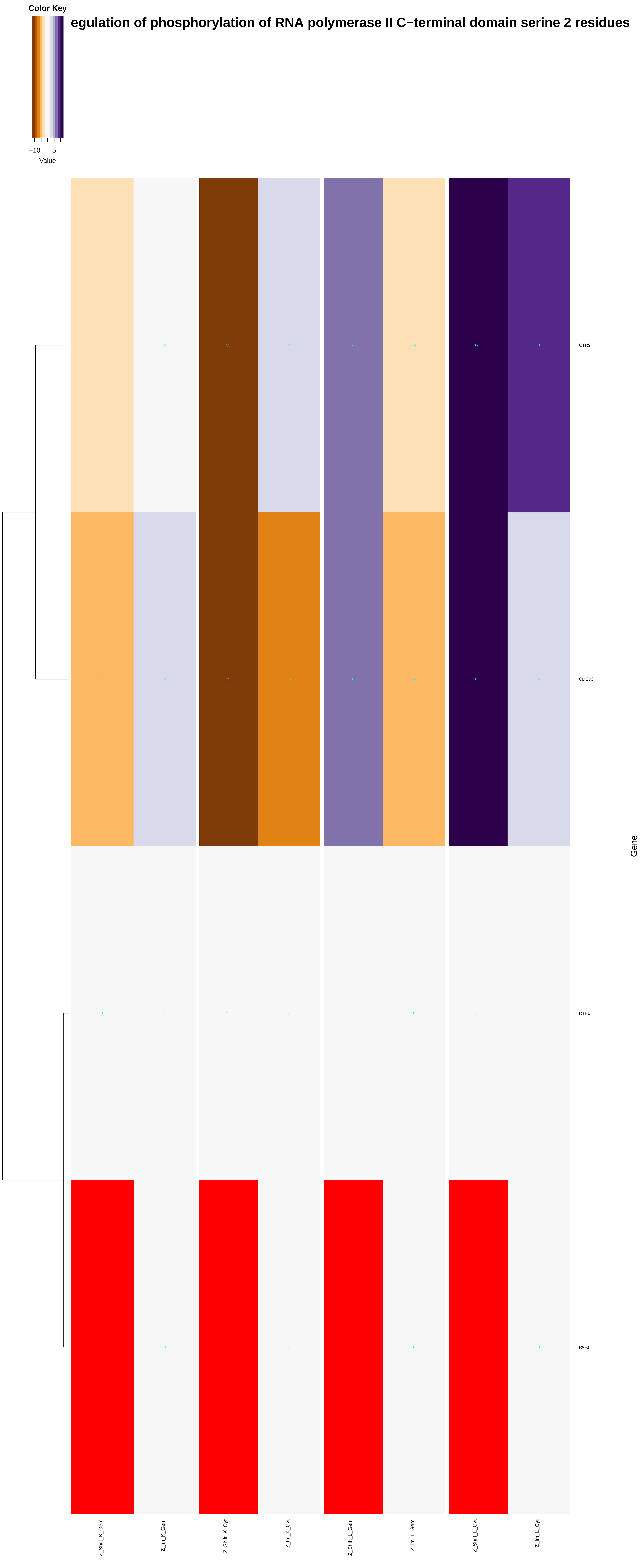
Color Key
−10
5
Value
egulation of phosphorylation of RNA polymerase II C−terminal domain serine 2 residues
CTR9
CDC73
RTF1
PAF1
Gene
Z_Shift_K_Gem
Z_Im_K_Gem
Z_Shift_K_Cyt
Z_Im_K_Cyt
Z_Shift_L_Gem
Z_Im_L_Gem
Z_Shift_L_Cyt
Z_Im_L_Cyt

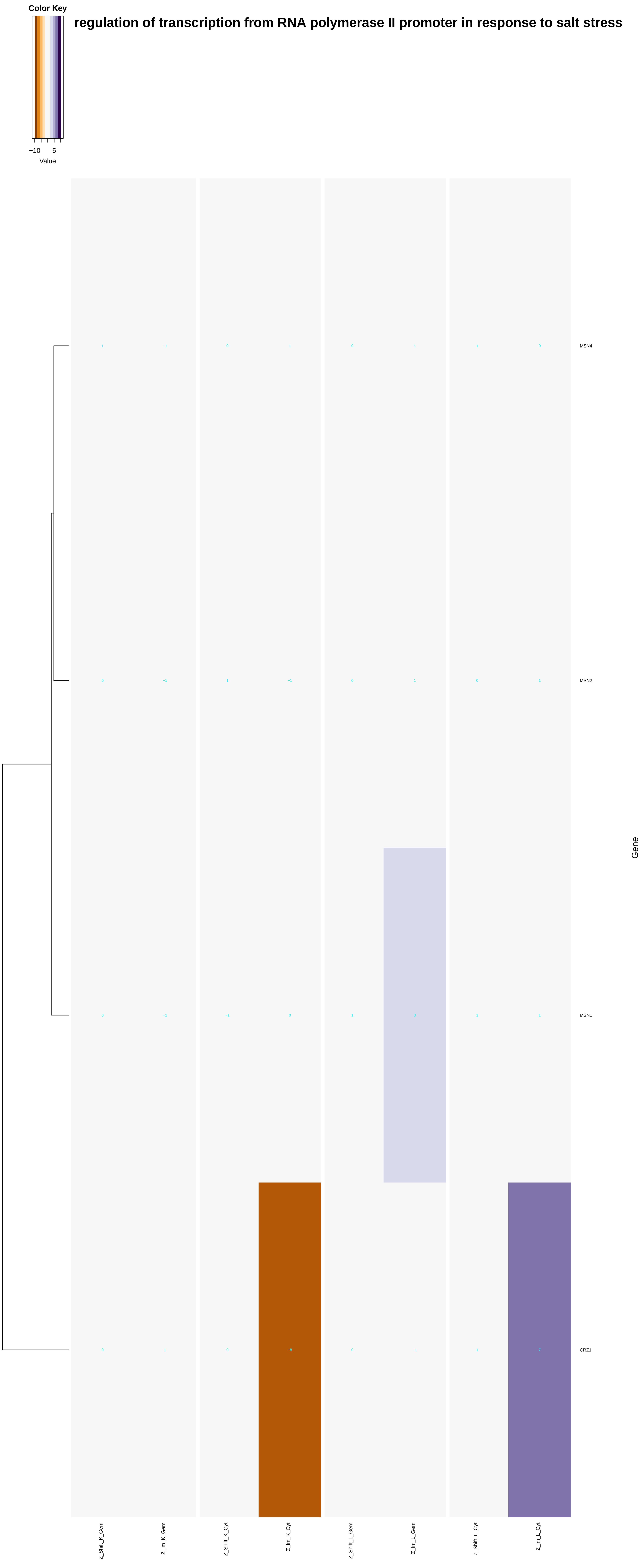

Color Key
−10
5
Value
regulation of transcription from RNA polymerase II promoter in response to salt stress
1
−1
0
1
0
1
1
0
MSN4
0
−1
1
−1
0
1
0
1
MSN2
0
−1
−1
0
1
3
1
1
MSN1
0
1
0
−8
0
−1
1
7
CRZ1
Gene
Z_Shift_K_Gem
Z_Im_K_Gem
Z_Shift_K_Cyt
Z_Im_K_Cyt
Z_Shift_L_Gem
Z_Im_L_Gem
Z_Shift_L_Cyt
Z_Im_L_Cyt

of starch catabolic process by positive regulation of transcription from RNA polymerase II p

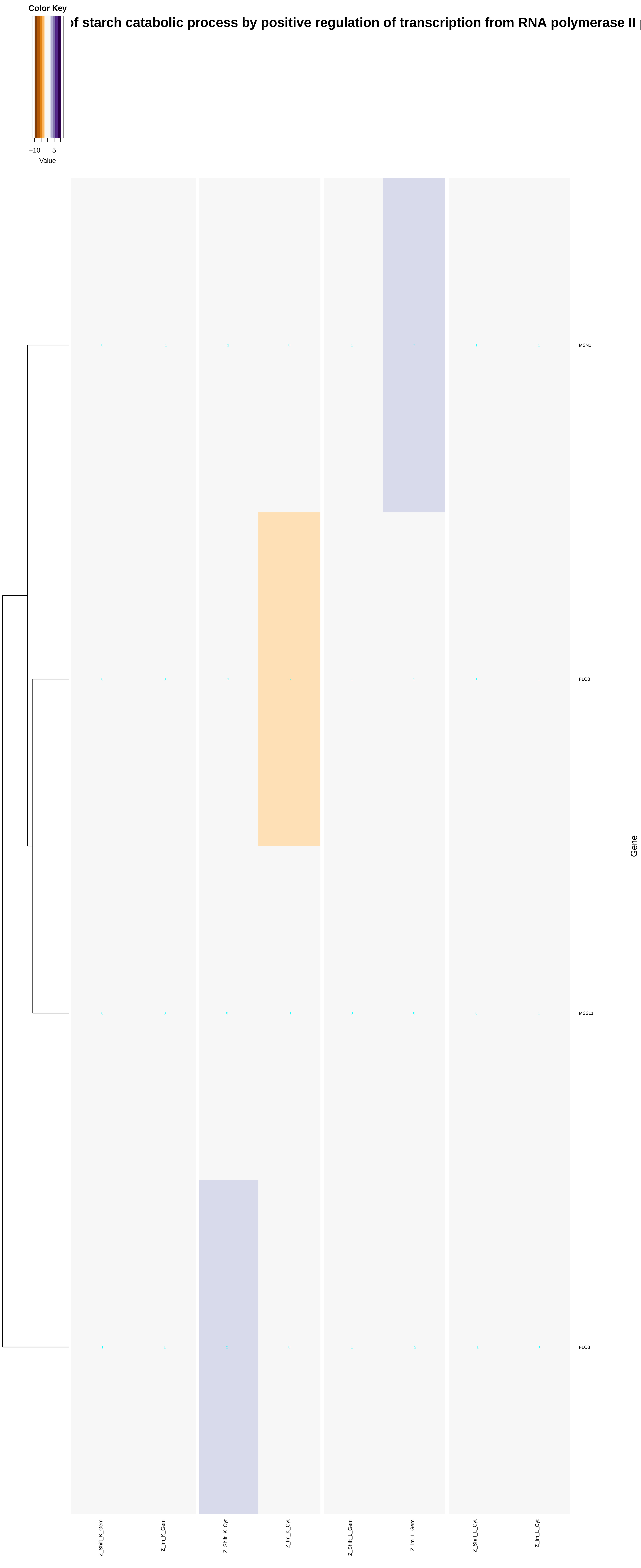

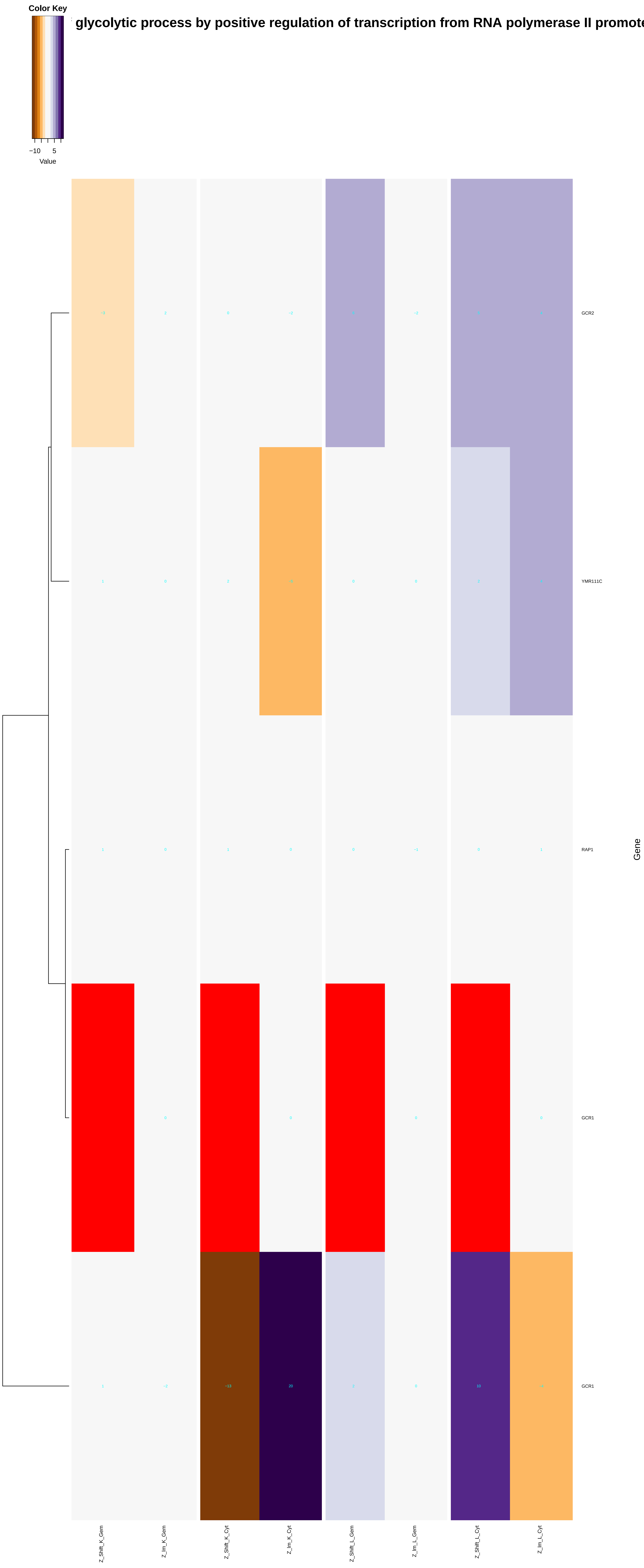
Color Key
−10
5
Value
glycolytic process by positive regulation of transcription from RNA polymerase II promote
GCR2
YMR111C
RAP1
GCR1
GCR1
Gene
Z_Shift_K_Gem
Z_Im_K_Gem
Z_Shift_K_Cyt
Z_Im_K_Cyt
Z_Shift_L_Gem
Z_Im_L_Gem
Z_Shift_L_Cyt
Z_Im_L_Cyt

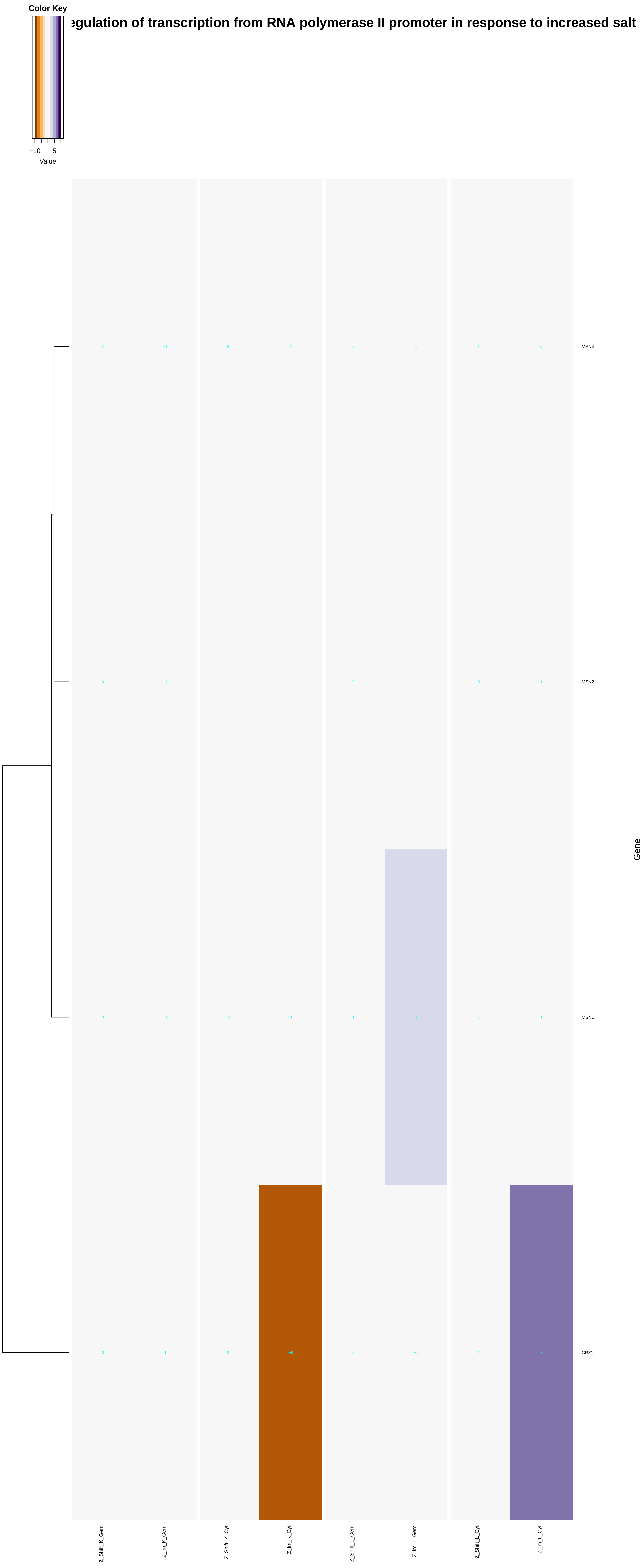

Color Key
−10
5
Value
egulation of transcription from RNA polymerase II promoter in response to increased salt
MSN4
MSN2
MSN1
CRZ1
Gene
Z_Shift_K_Gem
Z_Im_K_Gem
Z_Shift_K_Cyt
Z_Im_K_Cyt
Z_Shift_L_Gem
Z_Im_L_Gem
Z_Shift_L_Cyt
Z_Im_L_Cyt
